# Social learning dynamically shapes moral decision-making by biasing subjective valuation

**DOI:** 10.1371/journal.pbio.3003889

**Published:** 2026-07-10

**Authors:** Julien Benistant, Valentin Guigon, Alain Nicolas, Edmund Derrington, Jean-Claude Dreher

**Affiliations:** 1 CNRS, Institut des Sciences Cognitives Marc Jeannerod, Bron, France; 2 Université Lyon 1 Claude Bernard, Lyon, France; 3 Genopsy, CH Vinatier, Bron, France; Oxford University, UNITED KINGDOM OF GREAT BRITAIN AND NORTHERN IRELAND

## Abstract

Observing immoral behavior increases one’s dishonesty by social influence and learning processes. The neurocomputational mechanisms underlying such moral contagion remain unclear. We tested different mechanistic hypotheses to account for moral contagion. We used model-based fMRI and a new cheating game in which participants were sequentially placed in honest and dishonest social norm contexts. Participants’ cheating behavior increased in the dishonest norm context but was unchanged in the honest one. The best model to account for behavior indicated that participants’ valuation was dynamically biased by learning that others had cheated. At the group level, this valuation bias was not encoded by any specific brain region. Instead, this neural signal depended on individual differences in conformity, and engaged the bilateral lateral prefrontal cortex. During learning, simulation of others’ cheating behavior was encoded in the posterior superior temporal sulcus. Together, these findings provide a mechanistic understanding of how learning about others’ dishonesty biases individuals’ valuation of cheating but does not alter one’s established preferences. Observing others’ immoral behavior can increase one’s own dishonesty, but the neurocomputational mechanisms driving this moral contagion remain unclear. This study shows that cheating increases in dishonest contexts via learned valuation biases involving lateral prefrontal cortex and posterior superior temporal sulcus encoding others’ cheating behavior.

## Abstract 2

Observing others’ immoral behavior can increase one’s own dishonesty, but the neurocomputational mechanisms driving this moral contagion remain unclear. This study shows that cheating increases in dishonest contexts via learned valuation biases involving lateral prefrontal cortex and posterior superior temporal sulcus encoding others’ cheating behavior.

## Introduction

Dishonest behavior, such as cheating, tax evasion, and corruption is pervasive in modern societies. Dishonest behavior can be modified through exposure to others’ immoral behavior [1,2]. For example, observing others behave dishonestly increases one’s likelihood to cheat [2]. Such influence of others’ behavior on our own choices is not limited to dishonesty but can also be observed in domains such as risk-related decision-making [3–8] as well as pro-social and anti-social behavior [9–12]. Why and how observing others’ dishonest behavior modifies our own moral behavior in non-strategic settings remains an enduring puzzle. A growing literature has highlighted the importance of neurocomputational approaches for understanding how social information, moral preferences and value computations interact to shape human behavior. In particular, recent work has implicated specific brain regions in moral decision-making and described the underlying neural computations [13–17]. Yet, little is known about the neurocomputational mechanisms underlying how social influence modifies moral decisions.

It remains unclear whether social influence shapes individual beliefs, because behavior reflecting social norm compliance is not a perfect proxy for private beliefs. To date, two mechanisms have been proposed to explain how social influence affects one’s choices. The first, called valuation bias, proposes that individuals assign value to social information, which then influences their personal valuation process. This means that simply being exposed to others’ choices alters how individuals perceive the value of the options at stake and potentially influences their own choice [3,4,18,19]. The second mechanism, referred to as preference shift, proposes that exposure to others’ choices can more fundamentally modify an individual’s own preferences to align with the choices of others. This mechanism implies a more profound change in personal preferences and assumes that individuals become more similar to others in their social environment [5–8]. The two models differ in their assumptions with regard to how the decision-making process is affected. According to the valuation bias model, individuals place value on social information, such as others’ choices, and this additional value biases individuals toward others’ choices [3,4,18]. Conversely, in the preference shift model, individuals’ preferences themselves are changed. According to this latter model, individual preferences can be defined as the weights placed on the different components of a choice, such as monetary earnings, consequences for others or the morality of the action. Thus, individual preferences weight each component of the choice when computing decision value [18]. In this case, it is not the value of social information that leads to social influence but rather a convergence of individuals’ own preferences toward those of others [5–8].

One important limitation of these two previous accounts of social influence is that they do not consider the dynamic aspect of learning. For example, in a new social environment with specific norms (e.g., when one arrives in a new company), one usually lacks knowledge about others’ behavioral tendencies, including whether they lean toward honesty or dishonesty. Consequently, in such new environments, learning through repeated observation of others’ behavior becomes necessary to mitigate uncertainty about others’ true levels of (dis)honesty. Learning social norms in new contexts is particularly important regarding dishonesty because its concealed nature makes accurate observation challenging. Therefore, it is likely that a social learning component might modulate either the valuation bias or the preference shift to adjust how such mechanisms are weighted as the individual learns about others’ honesty levels.

**Fig 1:**
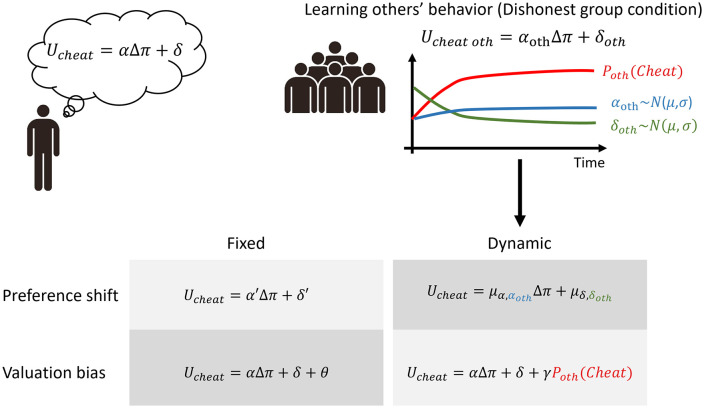
Graphical representation of the different computational models accounting for social influence. This figure represents the four different mechanisms of social influence that we considered. We varied whether social influence was due to a shift in one’s preferences (top row of the table) or a bias in the valuation process (bottom row). We then varied whether the process was fixed (left column) or dynamic because it depends on one’s learning about the behavior of others over time (right column) (schematically represented by the top figure, right column). For each model, a formal description is provided based on a utility function (*U*_cheat_) that represents the relative value of cheating for the individual, with two free parameters *α* and *δ*. *α* represents the individual’s sensitivity to monetary reward whereas *δ* represents the moral cost incurred when cheating. According to the Valuation bias Fixed model, the parameter *θ* represents the extent of the bias in the participants’ valuation of cheating. According to the Dynamic version, the parameter *γ* represents the level of conformity of the participant to others’ learned behavior (represented by $P_{Oth(Cheat)}$). Finally, in the Preference shift models, social influence is represented either by a fixed change in participants’ preferences *α* and *δ* (Fixed) or by a dynamic evolution of their preferences based on the learned preferences of others (Dynamic).

Here, we tested different theoretical accounts of the mechanisms underlying how others’ behavior influences one’s own cheating behavior. We considered four social influence mechanisms: a fixed or dynamic valuation bias, and a fixed or dynamic change in individual preferences (Fig 1). First, we assessed which computational model best explains social influence in cheating behavior. Second, we uncovered where in the brain different computational signals of social influence are encoded when we are presented with the opportunity to cheat. Importantly, the different accounts noted above predict that different neural mechanisms underlie social influence on cheating behavior. According to the first two hypotheses (fixed valuation bias and fixed preference shift hypotheses), no behavioral or brain activity changes should occur over time, because they do not consider that learning about the behavior of others would dynamically affect the influence process. In contrast, the dynamic valuation bias and dynamic preference shift hypotheses do predict such changes.

One brain region that could support such dynamic changes in social influence is the dorsolateral prefrontal cortex (dlPFC), as it represents others’ risk preferences during behavioral contagion [6]. Moreover, activity in the dlPFC is associated with integration of social norms and moral preferences in decision processes [16,20–22] and neuroimaging studies consistently implicate the dlPFC in dishonest choices [23–25]. Regions such as the right temporo-parietal junction (rTPJ) and the posterior superior temporal sulcus (pSTS) are also good candidates to support the dynamic integration of others’ behavior. Indeed, both the rTPJ and pSTS are central to tracking and integrating others’ preferences [26], and there is evidence that the rTPJ encodes others’ intentions necessary for social choices [27–30].

In addition to the tested mechanistic accounts, we also considered that inter-individual differences may shape how social information influences cheating behavior. Substantial variability exists in individuals’ tendency to conform to others and in their intrinsic honesty, and prior works show that such inter-individual differences systematically affect how social information is integrated into decision-making [6] and also influence how moral trade-offs are resolved [16]. We therefore further hypothesized that the dynamic social influence mechanisms captured by the tested computational models would vary across individuals as a function of their propensity to conform, and that their individual parameters would be linked to their brain activity during decision-making.

We used functional magnetic resonance imaging (fMRI) and a novel paradigm based on a cheating game. This game comprised two types of trials: (i) Solo trials, in which participants could lie about the outcome of a die roll to maximize their earnings, (ii) Predict trials, in which they predicted what another individual, randomly selected from a group of 10, reported in a previous experimental session. The purpose of the Solo trials was to assess the extent to which participants were prone to cheating. For the Predict trials, the goal was to expose participants to the behavior of others and allow them to learn the preferences of a group. Unbeknownst to the participants, the behavior of the group was simulated and controlled so that the participants faced either a dishonest or an honest group of players. With this manipulation, we assessed the effect of social influence in two different contexts. These two contexts have been shown to lead to different levels of conformity because antisocial and dishonest behavior appears to be more contagious than pro-social behavior [11,31,32]. The experiment was divided into three blocks. The first was composed of Solo trials and allowed us to estimate participants’ preferences in the absence of social influence. The second and third blocks consisted of interleaved Predict and Solo trials. In each of these two blocks, participants’ predictions concerned either an honest or a dishonest group of participants. This novel design gradually exposed participants to others’ behavior, allowing us to test the fixed *versus* dynamic accounts of valuation bias and preference shift mechanisms.

## Results

### Experimental design

**Fig 2:**
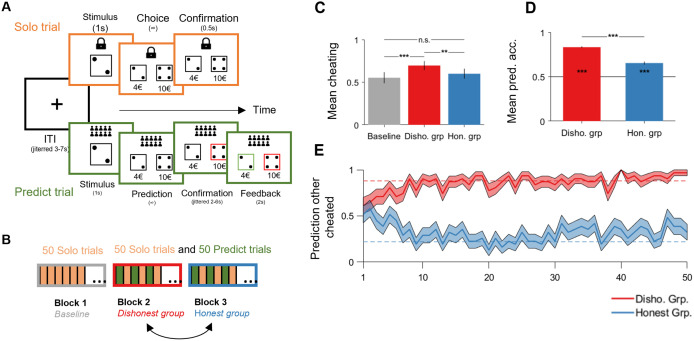
Experimental design and behavioral results. **A.** The experiment consisted of 2 types of trials. In the Solo trials, participants had to report the outcome of a die roll among two choices. An honest report was always less rewarded than a dishonest one, which made cheating the more profitable choice. The possible value of cheating on each trial varied, and ranged from €2 to €8 in increments of €2 between the honest (low payoff) and dishonest (high payoff) choices. The brute value of honest choices varied from 0 to 8 Euros and that for dishonest choices from 2 to 10 Euros. The Solo trials ended with a confirmation step in which the participants’ screen froze without confirmation of her choice, to reinforce the feeling that the decision was anonymous. In the Predict trials, participants had to predict whether another individual, randomly selected from a group of 10, cheated or reported honestly. Participants’ response was highlighted in red during a jittered confirmation step. Finally, participants received feedback about their prediction. The correct response was highlighted in green. **B.** Our experiment was divided into 3 blocks. The first one, the Baseline, consisted of 50 Solo trials and allowed us to assess the participants’ preferences. The two following blocks were a mixture of 50 Predict and 50 Solo trials that were interleaved. This structure allowed us to expose participants to others’ behavior and to assess the effect on participants’ decisions. In each of these two blocks, participants’ predictions concerned either an honest or a dishonest group of participants. The order of presentation of the Honest and Dishonest Groups was randomized between participants as either the second or third blocks. **C**. The mean proportion of cheating in the Dishonest Group condition was significantly higher than in the two other conditions. The statistical analysis was a pairwise comparison with Bonferroni correction extracted from a mixed-effect logistic regression (see model 1 in S2 Table for details). **D**. Mean prediction accuracy in both the Dishonest Group and the Honest Group conditions. The black line indicates chance level. Stars within bars indicate the results of a one-sided signed rank test comparing the prediction accuracy with the chance level. The other statistical analysis comes from a mixed-effect logistic regression (see model 2 in S2 Table for details). **E**. The mean of predictions that the other cheated over Predict trials for both the Dishonest and Honest Group conditions. The red (blue) dashed line corresponds to the mean proportion of cheating of the Dishonest (Honest) group members. In all figures, bars or shaded areas correspond to standard errors. *** *p* < 0.001, ** *p* < 0.01, * *p* < 0.05. The data underlying the figure can be found in the Figures folder of the OSF repository.

We scanned 32 participants using fMRI while they played the cheating game, which included two types of trials: Solo and Predict (Fig 2A). Solo trials required participants to observe a die roll result (‘stimulus’). Then, participants were presented with two dice (‘choices’), one presenting the original die roll result and the other showing an inaccurate die roll. Each die was associated with a different payoff. Notably, the accurate die always offered a lower payoff than the inaccurate one, which presented participants with a moral dilemma between honesty and maximization of their earnings. To modulate the difficulty of these dilemmas, the payoffs varied across trials. Participants had as much time as they wanted to choose which die roll to report. Once their choice was made, the screen froze for 500 ms (‘confirmation’). No visual feedback indicating their choice was provided to reinforce the feeling that the decision was made privately (see Methods for details).

The “Predict” trials mirrored the Solo trials but required participants to predict whether another individual would report their die roll accurately or not under similar circumstances. Participants were informed that, on each trial, an individual was randomly selected from a pool of 10 anonymous people who had previously completed the task. In reality, the group’s behavior was computer-simulated, which allowed controlled manipulation of their cheating behavior. Participants’ predictions were highlighted in red during a jittered ‘confirmation’ stage. Finally, at the end of each Predict trial, feedback was provided to the participant by highlighting the correct response in green. Accurate predictions were rewarded, whereas inaccurate predictions were not (see Methods for details).

The experiment comprised three blocks: a Baseline block of 50 Solo trials to discern individual preferences in the absence of social influence, and two subsequent blocks, each consisting of 50 Solo and 50 Predict trials presented in an interleaved manner (Fig 2B). This sequential arrangement aimed to gradually expose participants to others’ behavior, to test how learning about the others’ (dis)honesty influenced participants’ own decisions in the Solo trials. In these two blocks one block featured a dishonest group (Dishonest Group condition), whereas the other featured an honest group (Honest Group condition, see Methods and S1 Table). The presentation order of the latter two blocks was randomized across participants.

From the initial pool of 32 participants, one was excluded from all analyses due to constant cheating and disbelief that they were predicting real people’s decisions. Additionally, three more participants were excluded from the model-based analyses because they always cheated or always were honest in the Baseline Solo trials (see Methods). A translated version of the participants’ instructions and debriefing questionnaire is available in SI.

### Behavioral effect of social influence

In the Baseline condition, participants cheated in 55.3% of the Solo trials. They cheated in 69.6% of Solo trials in the Dishonest Group condition and in 60% of the Solo trials in the Honest Group condition (Fig 2C). To test the differences between each condition, we used a mixed-effect logistic regression on whether the participants cheated or not in a given trial with the following independent variables: a categorical variable coding for the condition (1: Baseline, 2: Dishonest Group and 3: Honest Group); a binary variable coding for the order of presentation of the Dishonest Group condition (1: first and 0: second); a variable coding for the trial number within each block; a variable coding for the difference between the payoff of cheating and honesty; and, a variable coding for the absolute difference between the die value of the cheating option and the honest one. We also added demographic variables such as the participants’ sex, age, and occupation (*i.e.,* student or not). All the regressions reported in this paper used the same independent variables except when indicated otherwise.

Participants were significantly more likely to cheat in the Dishonest Group condition than in any other condition (Fig 2C, margins contrast Dishonest *>* Baseline: 0.148 ± 0.037, *z* = 4.04, *p <* 0*.*001, Bonferroni correction for 3 comparisons; margins contrast Dishonest *>* Honest: 0.101 ± 0.027, *z* = 3.72, *p* = 0*.*001, Bonferroni correction for 3 comparisons; see model 1 in S2 Table). No significant difference in cheating behavior was observed between the Honest Group condition and the Baseline (Margins Honest *>* Baseline: 0.047 ± 0.025, *z* = 1.90, *p* = 0*.*170, Bonferroni correction for 3 comparisons).

To ensure that the observed differences were attributable to the groups’ actual behavior, and not differences between their learning performance with respect to the Honest and the Dishonest group’s behavior, we first tested whether participants’ prediction accuracy exceeded chance. Participants’ accuracy was indeed significantly above the chance level of 50% in both conditions (Wilcoxon signed rank tests, *z* = 4.87 and 4.49 for Dishonest and Honest Group conditions, respectively, *p <* 0*.*001 for both groups; Fig 2D). Moreover, Fig 2E shows that participants’ predictions of others’ cheating frequencies closely aligned with the groups’ mean cheating behavior in both conditions. Consistent with this, participants predicted cheating significantly more often in the Dishonest Group condition than in the Honest Group condition (Margins Dishonest *>* Honest: 0.540 ± 0.033, *z* = 16.2, *p <* 0*.*001; see model 3 in S2 Table). However, participants were also more accurate when predicting the Dishonest group’s behavior than that of the Honest group (Margins Dishonest *>* Honest: 0.179 ± 0.022, *z* = 8.20, *p <* 0*.*001; see model 2 in S2 Table). Therefore, we tested whether differences in prediction accuracy could account for the observed difference in cheating behavior between the Dishonest and Honest Group conditions. Using the same regression structure as model 1 in S2 Table but adding participants’ average prediction accuracy in each Group condition as a covariate, we found that including accuracy did not alter the effect of group condition on cheating probability (model 1, S3 Table). A second regression that controlled for each participant’s difference in prediction accuracy between conditions yielded the same result, i.e., the difference in cheating behavior across conditions remained unchanged (model 2, S3 Table).

We next examined whether the order of exposure affected cheating behavior or prediction accuracy. We included a regressor that indicates whether participants were first exposed to the Dishonest or the Honest Group condition. In the model 1 (S2 Table), this regressor was not significant, indicating that cheating behavior was not affected by the order of presentation (Margins Dishonest Group condition first = −0.067 ± 0.100, *z* = −0.67, *p* = 0.503). Furthermore, in the model 2 (S2 Table), this regressor was negative and significant showing that prediction accuracy was higher overall when participants began with the Dishonest Group condition (Margins Dishonest Group condition first = −0.045 ± 0.018 *z* = −2.52, *p* = 0.012). This might be due to the greater difficulty to predict the others’ cheating behavior in the Honest Group condition, which may have produced a spillover effect on the participants’ ability to predict the behavior of the Dishonest Group condition.

### Computational models of social influence

Our computational approach to analyze social influence encompassed two components: a social learning component and a social influence component. The social learning component accounts for how participants predicted others’ cheating behavior and updated their own beliefs about others’ preferences. The social influence component reflects how participants cheated (or not), based on their own preferences and based upon how they were influenced by what they learned about others’ cheating behavior. To simplify the selection process between our candidate models, we used a three-step selection procedure based on a group-level random-effect Bayesian model selection [33]. We started by defining which utility function, among the fixed moral cost and the variable moral cost, best described how participants chose between the honest and the dishonest options. Subsequently, we determined the best-fitting learning model that explained participants’ predictions in blocks 2 and 3. Finally, we conducted model selection among the four candidate models that represent the different social influence mechanisms. Below, we describe each of these steps in more detail.

#### Utility model selection.

First, we used the participants’ decision to cheat (i.e., choose the dishonest option) across all three blocks. Previous work shows that honesty-based choices can be described by two utility functions. A first utility function (Fixed cost) assumes that individuals endure a fixed moral cost when they are cheating [34,35]. A second utility function (Variable cost) assumes that the cost of being dishonest is dependent on the absolute gains associated with it [25] (see Methods SI). We considered extreme cases in which participants always used just one of these two utility functions (Fixed cost only or Variable cost only) and other cases in which they used one utility function in some blocks and the other in different blocks (Mixed variants). Results of the model selection showed that the utility function with a fixed moral cost of cheating best explained participants’ cheating behavior across all blocks (protected exceedance probability (pEP) = 0.999, S1B Fig).

#### Social learning model selection.

Next, we evaluated which model best explained participants’ social learning behavior during the Predict trials in the Dishonest and Honest Group conditions. We tested various candidate models derived from the Bayesian Preference Learning (BPL) model [36]. These models assume that participants start with normally distributed priors regarding others’ preferences, which allows participants to infer the likelihood of cheating when they predict others’ choices during the Predict trials. Post-feedback, a prediction error, that compares their prediction with the actual behavior, refines the posterior distribution of others’ preferences. Within the BPL model, we considered three types of priors, each representing distinct learning biases. The first involved participants using their own preferences as priors for both conditions, a process termed self-projection (BPL-Self). The second utilized participants’ priors based on their own preferences for the initial group condition and the learned preferences from the preceding group for the subsequent group condition (BPL-Others). This captures the persistence of prior learning. Finally, the third model combined aspects of the previous two types of priors. When learning about the second group’s behavior, participants combined their own preferences with the learned preferences regarding the previous group (BPL-SelfOthers). Furthermore, we investigated whether participants inferred others’ preferences according to the utility functions previously mentioned. Specifically, participants could believe that others’ decisions aligned with the utility function used to simulate others’ behavior (Variable cost of cheating, [25]), or they might consider the utility function they used for their own decisions (Fixed cost of cheating) ([34,35]; see Methods). We found that the BPL model with self-projection (BPL-Self) and the utility function with a fixed moral cost of cheating was the best to explain our participants’ predictions (pEP: 0.986, S1C Fig).

#### Social influence model selection.

Finally, we proceeded to the selection of the model that best explained social influence in our experiment. We explored two hypotheses, each delineating social influence in distinct ways: one as a fixed phenomenon, independent of participants’ learning about others’ cheating behavior; and the other as a dynamic and learned phenomenon, interconnected with participants’ understanding of others’ conduct.

For the fixed hypothesis, we evaluated two models. The first posits that social influence arises from a shift in individuals’ preferences (Preferences Shift, PS_Fixed_) [5–7,12]. The second suggests that social influence results from a bias in the valuation process (Valuation Bias, VB_Fixed_) [3,4,18]. For the dynamic hypothesis, we examined versions of these models in which influence correlated with what participants learned of others’ cheating tendencies across trials. Thus, in the PS_Dynamic_ model, preference change was hypothesized to result from a weighted average between participants’ own preferences and the inferred preferences of others at a given time (PS_Dynamic_ model). This weighted average is governed by two distinct free parameters, γA and γD, for each parameter of the others’ utility function (γA for the parameter α and γD for the parameter δ). They are specific to each group condition, indicating the participants’ levels of conformity. For the VB_Dynamic_ model, we considered that the decision value in participants’ choice models is modulated by the probability that others would have cheated (or been honest) in the Dishonest Group (Honest Group) condition during a particular Solo trial, as calculated by the BPL Self model (VB_Dynamic_ model). A parameter, γ, adjusts the probability and represents the extent of participants’ conformity to others’ behavior. This parameter was individually estimated for each participant in each group condition.

**Fig 3:**
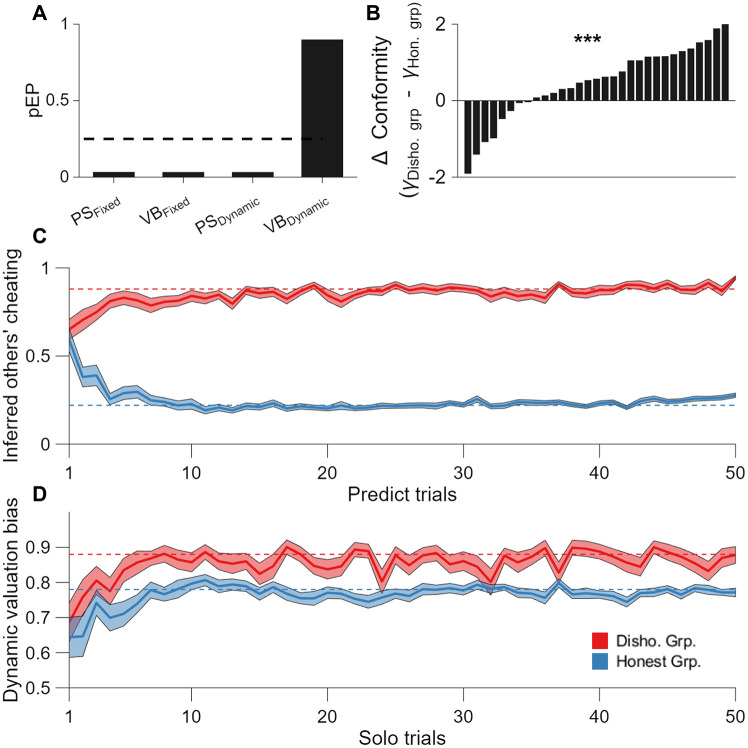
Model selection and estimation. **A**. Protected Exceedance Probability (pEP) of the different candidate models. The higher the pEP, the more likely a given model explains the group’s behavior relative to the others. PS, Preferences shift; VB, Valuation Bias. The dashed line indicates chance level. **B**. Difference between the value of the conformity parameter *γ* in the Dishonest Group condition and the conformity parameter *γ* in the Honest Group condition for each participant. The stars represent the result of a two-sided rank-sum test between the two conditions. **C.** Mean inferred probability that others cheated during Predict trials in the Dishonest Group condition (red) and the Honest Group condition (blue). Shaded areas are standard errors. The red (blue) dashed line corresponds to the mean proportion of cheating of the Dishonest (Honest) group members. **D**. Mean dynamic valuation bias over Solo trials in the Dishonest Group condition (red, probability that others would have cheated) and in the Honest Group condition (blue, probability that others would have been honest). Shaded areas are standard errors. The red (blue) dashed line corresponds to the mean proportion of cheating (honest behavior) of the Dishonest (Honest) group members. *** *p* < 0.001. The data underlying the figure can be found in the Figures folder of the OSF repository.

In total, we tested four models (see Fig 1 for a graphical summary). The Bayesian model selection showed that the VB_Dynamic_ model was the most frequent best fit across our sample (pEP = 0.899; Fig 3A, additional metrics are reported in S1A Fig and additional candidate are tested in S1D Fig). As a robustness check, we conducted a model-recovery analyses on the four models and a parameter recovery on the VB_Dynamic_ model (S2A, S2B, and S3A Figs). We also ran similar analyses on the two utility functions (Fixed and Variable cost, S2C, S2D, and S3B Figs).

We then explored the estimated parameters of the winning VB_Dynamic_ model. First, the parameter *α* (mean: 0.235 ± 0.021, Wilcoxon signed rank test, *z* = 4.86, *p* < 0.001, S4A Fig) was significantly positive, as well as the inverse temperature parameters *β* (mean: 2.821 ± 0.293, Wilcoxon signed rank test, *z* = 4.86, *p* < 0.001, S4A Fig) and *β*_Oth_ (mean: 1.100 ± 0.062, Wilcoxon signed rank test, *z* = 4.86, *p* < 0.001, S4A Fig). However, these parameters are constrained in our model to be significantly greater than zero. Conversely, *δ* is negative and significantly different from zero (mean: −0.727 ± 0.190, Wilcoxon signed rank test, *z* = −3.41, *p* = 0.001, S4A Fig). Overall, this was consistent with participants’ likelihood of cheating increasing with the relative earnings of cheating, while being limited by a fixed moral cost associated with cheating.

Second, we observed that the conformity parameter γ is significantly higher in the Dishonest Group condition than in the Honest Group condition (Two-sided rank sum test, *z* = 3.45, *p* < 0.001; Figs 3B and S4B). Additionally, the parameter *γ* is significantly different from zero in the Dishonest Group condition (mean: 0.473 ± 0.106, Wilcoxon signed rank test, *z* = 3.45, *p* < 0.001, S4B Fig) but not in the Honest Group condition (mean: −0.013 ± 0.094, Wilcoxon signed rank test, *z* = 0.02, *p* = 0.984, S4B Fig). This indicated no reliable conformity effect in the Honest Group condition while there is a clear conformity effect in the Dishonest Group condition. Indeed, higher *γ* values indicated a greater tendency to align with others’ behavior (more cheating in the Dishonest Group condition and less cheating in the Honest Group condition, see S5A Fig for a simulation). Furthermore, we examined whether an analog of *γ* could be observed in the raw data by correlating the fitted *γ* parameters with participants’ change in cheating relative to baseline (mean cheating in each Group condition minus mean cheating in the baseline). These correlations were in the expected direction, positive in the Dishonest Group condition and negative in the Honest Group condition, although the association was weak and only significant in the Honest Group condition (S5B Fig). Thus, aggregate changes in cheating provide a coarse behavioral proxy for *γ*, but should not be interpreted as a direct equivalent of the fitted conformity parameter. This is because such aggregate measures do not capture the dynamic aspect of conformity in our experiment. Finally, one could argue that participants’ conformity may be explained by their own preferences or by the accuracy of their predictions. Yet, these alternative hypotheses are not supported by additional analyses which show that *γ* is neither correlated with the participants’ own moral cost *δ*, nor with participants’ average prediction accuracy (S5C–S5E Fig). However, we cannot completely rule out these alternative explanations with the present experimental design.

**Fig 4:**
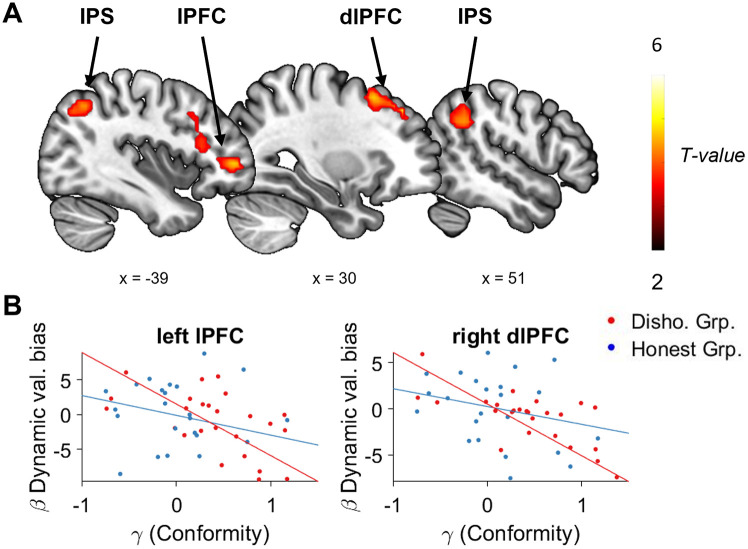
Brain regions encoding the dynamic valuation bias. **A.** Negative relationship between the BOLD signal in the left lateral prefrontal cortex (lPFC) and the dynamic valuation bias signal (i.e., the learned probability that the other would have cheated), as a function of participants’ conformity parameter *γ*, at the decision stage of the Solo trials of the Dishonest Group condition (*p* < 0.001 uncorrected and *p* < 0.05 whole-brain cluster-corrected family-wise error (FWE)). **B.** Individuals’ beta extracted from 6 mm sphere centered on peak activity in the left lPFC (right dlPFC) are negatively correlated with their conformity parameter *γ* in the Dishonest Group condition.

Third, concerning the learning of others’ preferences, we found that the predicted probability that others would cheat, estimated by participants, converged towards the average simulated probability to cheat of each group (Fig 3C). We also found that the dynamic valuation bias converged towards the mean probability to cheat (to be honest) for the Dishonest (Honest) Group condition (Fig 4C).

Formally, the learned α parameters are significantly higher for the Dishonest Group than the Honest Group (mean α, Dishonest group: 0.437, Honest Group: 0.090, two-sided rank sum test, *z* = 4.78, *p* < 0.001; S4C Fig). We also found that both learned *α* parameters are significantly positive (Wilcoxon signed rank test, *z*(Dishonest, Honest) = 4.86, *p* < 0.001). Conversely, the learned moral cost *δ* parameters are significantly lower for the Honest Group than the Dishonest Group (mean *δ*, Dishonest group: −0.089, Honest Group: −1.736; two-sided rank sum test, *z* = 6.46, *p* < 0.001; S4D Fig). Moreover, the learned moral cost *δ* parameter is significantly different from zero in the Honest Group condition (Wilcoxon signed rank test, *z* = −4.86, *p* < 0.001) but not in the Dishonest Group condition (Wilcoxon signed rank test, *z* = −1.10, *p* = 0.272). This implies that participants recognized that members of the Dishonest Group were more likely to cheat than members of the Honest Group for a given set of payoffs. However, the variance parameter of the distribution for the learned parameter *α* is significantly higher for the Honest than the Dishonest Group. Conversely, the variance parameter of the distribution for the learned parameter *δ* is significantly higher for the Dishonest Group than the Honest one (mean var. *α*, Dishonest group: 0.396, Honest Group: 0.855; mean var. *δ*, Dishonest Group: 0.531; Honest Group: 0.312; two-sided rank sum tests, *z* = −4.41, *p* < 0.001 and *z* = 3.18, *p* = 0.001, respectively). Furthermore, the difference in variance for the parameter *δ* is significantly lower than the difference for the parameter *α* (*α* diff: *M* = −0.459, *δ* diff: *M* = 0.219, two-sided rank sum test, *z* = −5.86, *p* < 0.001). This finding parallels the behavioral results which indicate that participants’ prediction accuracy is lower in the Honest Group condition than in the Dishonest Group condition.

Finally, we also examined pairwise correlations between fitted parameters (S4E Fig). This analysis revealed a negative correlation between *β* and *δ*, suggesting that participants with stronger moral costs tended to show less stochasticity in their choices. We also observed a negative correlation between *γ*_D_ and *γ*_H_, indicating that participants who conformed more strongly to the Dishonest Group tended to conform less to the Honest Group. As a final robustness check, we examined whether participants’ explicit reports of changes in moral beliefs about cheating affected the behavioral and computational results. Based on the debriefing questionnaire, 13 participants reported a change in their beliefs about the morality of cheating in the task, whereas 18 did not. Additional regression analyses showed no significant differences between these two groups in cheating behavior, prediction accuracy, or predicted cheating behavior (S4 Table). Moreover, behavior in both groups was best explained by the VB_Dynamic_ model (pEP > 0.98 for both groups), and the fitted parameter values did not differ significantly between groups (Wilcoxon rank-sum tests, *p* > 0.05 for all parameters). Thus, the main behavioral and computational results were not driven by participants who reported a change in explicit moral beliefs about cheating.

### Model-based fMRI analysis

We constructed three GLMs to identify how the brain encodes the 4 computational signals of the VB_Dynamic_ model. The first three signals concerned the decision and prediction processes (at the time of choice in the Solo trials and the time of prediction in the Predict trials): (1) the dynamic valuation bias in the Dishonest and Honest Group conditions (choices in the Solo trials); (2) the probability associated with participants’ predictions, when observing Dishonest and Honest group members (predictions in the Predict trials), (3) the relative value of chosen and unchosen options in Solo trials, regardless of conditions (i.e., all blocks averaged together). The fourth signal corresponded to the prediction error at the feedback stage of the Predict trials. Four parametric regressors of no interest were also added: participants’ response times, choice side (left or right), decisions to cheat or not at the choice stage of the Solo trials and their predictions about others’ behavior during the prediction stage of the Predict trials. All the parametric regressors were orthogonalized due to a strong correlation between the parametric regressors of interest and those of no interest. Finally, we added the participants’ degree of conformity *γ* for both the Dishonest and Honest Group conditions as a second-level covariate.

In the first GLM (GLM 1), we included only the first signal (dynamic valuation bias) as a parametric regressor during the choice phase of the Solo trials in both the Dishonest and Honest Group conditions (see Methods). The purpose was to assess whether some brain regions encoded this signal in the two conditions. This GLM revealed no significant correlation between brain activity and the dynamic valuation bias in the Solo trials of either Group condition (*p* > 0.001 uncorrected). One possible explanation for the absence of a group-level effect is that the neural encoding of the dynamic valuation bias depends on individual differences in conformity to others’ behavior. Specifically, participants who behave against the observed norm (e.g., cheating less than the Dishonest Group or more than the Honest Group) may encode this signal differently from participants whose behavior aligns with that of others. Consistent with this interpretation, when focusing on the Dishonest Group condition, a second-level regression analysis revealed that the relationship between the dynamic valuation bias and brain activity varied as a function of participants’ conformity parameter γ. In particular, activity in the left Lateral Prefrontal Cortex (lPFC), the right dorsolateral Prefrontal Cortex (rdlPFC) and bilateral Inferior Parietal Sulcus (IPS) encoded the dynamic valuation bias which was inversely proportional to the conformity parameter, *γ*, for the Dishonest Group condition (lPFC, *x*,*y*,*z* = −48,42,0; rdlPFC = 30,39,42; IPS = −33, −63,45 and 36, −66,39; Fig 4A and S5 Table; *p* < 0.05 FWE whole-brain cluster corrected). To illustrate this effect (Fig 4B), individuals’ betas were extracted from 6 mm spheres centered on peak activity in the bilateral lPFC in the Dishonest and Honest Group condition. For negative values of *γ*, participants show anti-conformity while positive *γ* values reflect conformity.

**Fig 5:**
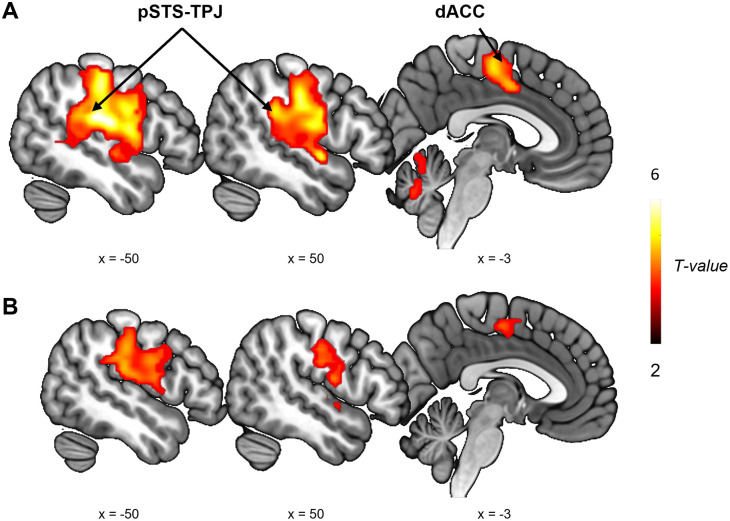
Brain regions involved in learning others’ behavior. **A.** The BOLD signal in the bilateral posterior superior temporal sulcus (pSTS), the temporo-parietal junction (TPJ) and the dorsal anterior cingulate cortex (dACC) correlates with the probability associated with participants’ predictions in the Dishonest Group condition at the prediction stage of the Predict trials (*p* < 0.001 uncorrected and *p* < 0.05 whole-brain cluster-corrected family-wise error (FWE)). **B**. Contrast between the brain activity in the Dishonest and Honest Group conditions that correlates with the probability associated with participants’ predictions at the prediction stage of the Predict trials (*p* < 0.001 uncorrected and p < 0.05 whole-brain cluster-corrected family-wise error (FWE)).

In the second GLM (GLM 2), we added the remaining three computational signals: the probability associated with participants’ predictions at the time of the prediction in the Predict trials, their prediction error at the time of the feedback in the Predict trials and the relative value of the participants’ choice at the choice stage of the Solo trials in every condition (Baseline and Groups; see Methods SI for details). This GLM revealed that the probability associated with participants’ predictions in the Dishonest Group condition is encoded in a large cluster that includes the bilateral posterior Superior Temporal Sulcus and Temporo-Parietal Junction (pSTS-TPJ) and the dorsal anterior cingulate cortex (dACC; cluster’s peak, *x*,*y*,*z* = 57,6,24 and −27,30,27; see Fig 5A and S6 Table; surviving a FWE-peak correction, see S5A Fig for Betas extracted from an independent ROI). Furthermore, a contrast between the Dishonest and Honest Group condition showed that a part of this cluster activity was significantly greater in the Dishonest Group condition than in the Honest Group condition (cluster’s peak, *x*,*y*,*z* = −18, − 18,3 and 60,6,27; see Fig 5B and S7 Table; *p* < 0.05 FWE whole-brain cluster corrected). When looking for regions encoding this computational signal in the Honest Group condition, no voxels survived the cluster-FWE correction (*p* > 0.05). Additionally, the relative value of the chosen option (${DV}_{Choosen}-{DV}_{Unchoosen})$ in the Solo trials was negatively correlated with the BOLD signal in the dorsal Anterior Cingulate Cortex (dACC, *x*,*y*,*z* = 18,27,48). However, this cluster did not survive the cluster FWE correction (*p*(FWE-corrected) = 0.203). Finally, the prediction error at the time of the feedback in the Predict trials, correlated with a large cluster encompassing the bilateral ventral striatum (*x*,*y*,*z* = 18, − 15,24; S5B Fig and S8 Table).

## Discussion

Individuals possess only a limited understanding of others’ opinions, intentions and preferences. Yet, social influence hinges on the ability to infer others’ likely actions. Our findings indicate that when we observe others’ cheating behavior, our brain monitors their inclinations toward dishonesty, not by shaping our own moral preferences, but by biasing our assessment of the value of cheating (valuation bias). Moreover, this valuation bias is dynamic. It reflects progressive learning about the moral inclinations of others. At the brain level, participants conformity modulates how the dynamic valuation bias is encoded in the left lPFC and right dLPFC when observing dishonest behavior. Moreover, others’ dishonesty level is encoded in the TPJ-pSTS when participants predict their behavior. These results provide a dynamic and mechanistic understanding of social influence in cheating, and demonstrate a previously uncharacterized interplay between social learning and social influence processes.

At the behavioral level, participants changed their cheating behavior when they were exposed to the Dishonest Group, but not the Honest Group. One possible explanation for this asymmetry is that participants learned more accurately about the Dishonest Group than the Honest Group. Consistent with this idea, prediction accuracy was indeed higher in the Dishonest condition. However, in regression analyses, when we controlled for differences in prediction accuracy, the effect of group condition on cheating behavior remained unchanged. Furthermore, the conformity parameter estimated by the VB_Dynamic_ model was significantly larger when participants observed the Dishonest Group than the Honest Group. This indicates a stronger tendency to align behavior with dishonest norms. Importantly, these conformity parameters did not correlate with participants’ mean prediction accuracy. These additional analyses show that, while learning accuracy differs between conditions, it cannot account for the asymmetric impact of social influence. Overall, these results are consistent with research showing that anti-social behaviors such as cheating are more contagious than pro-social behavior or honesty [11,31,32]. In addition, social proximity exacerbates this asymmetry [11] and can improve social learning [37]. However, in our experiment, group members were anonymous. Thus, the asymmetry of influence cannot be explained by different levels of social proximity between the participants and others.

In our experimental settings, participants’ cheating decisions were observable to the experimenters at the individual level. Previous studies have shown that such observability can influence behavior, even when participants are informed that their decisions are anonymous [35,38]. This limitation is inherent to fMRI-based, model-driven approaches, which require trial-by-trial observation of individual decisions. To mitigate potential concerns, participants were clearly instructed that their choices were anonymous and that data analysis would be conducted by an experimenter different from the one present during the scanning session, under strict anonymity (see instructions in SI). Importantly, the degree of observability was held constant across all experimental conditions. Moreover, recent evidence suggests that even higher levels of observability than those present in the current study do not substantially alter the influence of empirical social norms on dishonest behavior [39]. It is therefore unlikely that the observability of participants’ decisions can account for the differences observed between conditions in our experiment.

Two main mechanisms of social influence have been proposed previously: an adjustment in individuals’ preferences [6,12,19] or a bias in the individuals’ valuation process [3,4]. Previous work proposed that these two mechanisms are dependent on the accessibility of social information. Specifically, when individuals have full access to others’ behavior or beliefs, preference shifts may be more likely to occur [18]. In the present study, however, we find that a dynamic valuation bias provides a better account of social influence even when others’ behavior is fully accessible and accurately learned. Our model assumes that participants dynamically infer the preferences of others over time (social learning component). This internal representation subsequently biases participants’ valuation process when confronted to the opportunity to cheat (social influence component). This knowledge leads to a change in the value people attribute to the different options. Rather than implying blind imitation or a convergence of preferences, this mechanism is consistent with the view that social influence involves constructing a causal understanding of others’ behavior, which then informs individual decision-making [28,40,41].

One original aspect of our experiment is the gradual exposure of participants to others’ behavior. Past experiments have either presented others’ choices in a block of trials or did not consider the learning dynamics [4,6]. In our task, participants learned relatively quickly, as they were able to predict others’ behavior accurately after approximately 10 trials. While this rapid learning may limit the range over which learning dynamics can be observed, our dynamic model of social influence nevertheless provided a better account of participants’ behavior than fixed models, which suggests that static approaches may not fully capture the processes underlying social influence. A natural extension of this work would be to examine more volatile or uncertain social environments, which would be expected to slow learning and further differentiate dynamic from fixed mechanisms [42].

Our findings also shed light on the neurocomputational mechanisms that support social influence. When participants were exposed to a Dishonest Group, we observed differences in dynamic valuation bias-related activity in the right dlPFC and left lPFC as a function of individual conformity levels. Specifically, greater valuation bias-related activity in these regions was associated with lower conformity, which reflects increased resistance to the observed cheating social norm of the dishonest group. Notably, we did not observe a main effect of the dynamic valuation bias at the group level, which indicates that this neural signal is not uniformly expressed across individuals but rather depends on individual differences in conformity.

This neural pattern mirrors the behavioral results which show that larger valuation biases were associated with a higher likelihood to cheat. The observation that the lPFC dynamically tracks valuation bias extends previous work showing that this region encodes others’ perspectives on choice behavior, whether moral or non-moral [6,16], and plays a role in moral and ethical decision-making through processes of self-regulation, integration of moral values [14,16,24,43–45], and norm compliance [22,46]. Importantly, prior studies examined contexts in which social norms were explicitly known from the outset, rather than learned over time as in the present experiment. Here, we show, additionally, that the lPFC tracks the evolution of the social norm (i.e., the behavior of others) depending on the participants’ degree of conformity. Together, these findings suggest that the lPFC dynamically monitors the social environment and supports the integration of social information into the valuation processes that guide decision-making.

In our BPL model, based on the model developed by [36], participants start with priors about the preferences of the others, which are then used to predict their likelihood to cheat. A prediction error updates those priors and corresponds to the difference between the predicted likelihood to cheat and the other’s actual behavior. During the feedback phase, this prediction error was encoded in the bilateral ventral striatum. This region has been demonstrated to encode prediction errors related to social norms, a process required for learning about the behavior of other individuals [47,48].

When examining brain regions involved in representing beliefs about the dishonesty of group members, we found that activity in the bilateral pSTS and TPJ tracked the probability associated with participants’ predictions during the prediction phase. Notably, this effect was present in the Dishonest Group condition but not in the Honest Group condition. This pattern is consistent with the behavioral asymmetry in learning observed between conditions and aligns with previous findings implicating these regions in simulating others’ learning and representing others’ preferences [26,49]. These results suggest that the pSTS–TPJ supports the computation of signals required for learning and predicting others’ behavioral tendencies, particularly in contexts in which participants show conformity to dishonest behavior.

Importantly, we found no evidence that the pSTS–TPJ directly mediates the social influence process itself, as the dynamic valuation bias was encoded in the bilateral lPFC. In contrast, prior studies reporting pSTS–TPJ involvement in decision-making typically examined situations in which social information was directly relevant for choice, such as cooperation [50], consensus formation [26] or when revising one’s choice [51]. In the present study, participants did not require social information to decide whether to cheat; rather, their decisions could be indirectly influenced by what they learned about others’ behavior. Consistent with this interpretation, previous work on social influence has not observed pSTS–TPJ engagement when social learning was absent [4,8].

The behavior of the individuals used to generate the group decisions was simulated using a utility model that included a variable cost of cheating, whereas the BPL model that best described participants’ own decisions and learning relied on a model which included a fixed cost of cheating. In principle, such a mismatch could make learning about others’ cheating behavior more difficult, as participants would be inferring patterns generated by a model different from their own. However, our behavioral results suggest that participants were nevertheless able to form accurate beliefs. Their predictions were significantly better than chance and converged toward the true mean cheating level in both Group conditions. Several factors may explain the discrepancy between the generative model of the pilot participants and the model that best fits our main sample. First, the pilot participants completed the task in a different context (outside the MRI scanner), which may have influenced their decision strategies. Second, unobserved demographic or psychological differences between pilot and main-sample participants could also contribute to differences in model parameters. Nonetheless, our analyses indicate that participants’ ability to learn about others’ behavior was not impaired in a way that would account for the observed asymmetry in conformity.

A limitation of our study is that the parameter-recovery analyses do not perfectly support the validity of our model-fitting procedure (S3A Fig). This likely reflects the fact that our experimental design was not originally optimized for parameter identifiability across multiple complex components, namely social learning and social influence, which are estimated together in the VB_Dynamic_ model. Specifically, our parameter recovery was weak for one parameter, the others’ temperature parameter (*β*_Oth_) and limited for two parameters (*α* and *β*). However, the main parameters of interest in the VB_Dynamic_ model, i.e., the conformity parameters γH and γD, were relatively well-recovered. The limited recoverability of the temperature parameter may reflect, at least in part, the rapid learning observed in participants. Faster learning likely reduced variability in the inferred relative value of cheating across Predict trials, thereby constraining the information available to estimate parameters related to decision stochasticity. Given that the temperature parameter primarily modulates variability in prediction behavior during social learning, this reduction in variability may have contributed to its poor recovery. Thus, these model-based results should be interpreted with caution. However, they constitute a first step toward a better integration of social learning into social influence processes. Future work may build on the present framework using experimental designs tailored to improve parameter identifiability, by increasing environmental volatility or uncertainty to slow learning dynamics.

Overall, our model explains how individuals generate a formal representation of others’ preferences which then biases their valuation process. We report that the pSTS-TPJ and bilateral lPFC compute two separate signals: a social learning signal in the pSTS-TPJ and a social influence signal in the bilateral lPFC. These computational roles elucidate the dynamic link between social learning and social influence in the context of cheating behavior. The representation of others’ behavioral tendencies is encoded in the pSTS-TPJ region during learning, whereas the dynamic bias engages the left lPFC and right dlPFC. These findings have implications for developing new computational theories of social influence that could be useful in different contexts. For example, a recent fMRI study reported that cognitive processes underlying social understanding were more aligned after natural consensus-building conversation, often used for social influence [52]. Much research on social influence has focused on public compliance, setting aside the long-lasting effects of social interaction on private cognition [53,54]. In fact, consensus-building conversation not only aligns neural responses within groups (reflecting social compliance), but this alignment can generalize to novel stimuli that were not discussed [52]. This emphasizes an important role of subjective valuation of others’ learned preferences (an effect explained here by the valuation bias) in understanding social influence. More broadly, our study highlights how social learning dynamically shapes moral decision-making by biasing subjective valuation processes rather than altering stable moral preferences. Such mechanisms may constitute a general computational principle through which social environments shape individual decision-making across domains. Understanding these dynamic social influence processes may be critical for explaining how dishonest or honest norms propagate within social networks.

## Methods

### Participants

Thirty-two healthy participants were recruited *via* the Facebook recruitment page of the Institute of Cognitive Sciences. Exclusion criteria included a history of systemic or neurological disorders, psychiatric disorders, psychoactive medication or drug use, pregnancy, involvement in psychology classes, and previous participation in studies involving decision-making in general. We recruited only right-handed participants. One participant was excluded from any analysis because they cheated in all the trials and reported that they did not believe the scenario, leaving 31 participants (14 males, mean age: 22.87 years, s.d. 0.58). For the model estimation and the fMRI results three additional participants were excluded because they never cheated in the Baseline condition. Thus, these analyses are made on 28 participants.

Our sample size was estimated based on an effect size extracted from the results of Suzuki and colleagues, 2016 [6]. Based on the peak activity in the rdlPFC relating to the beliefs updating (*t*-value = 6.70, *p*-val < 0.001), we estimated a large effect size (Cohen’s *f* = 0.4). To achieve 80% power with a 5% significance level in a linear multiple regression with 20 co-regressors we calculated that we would need a minimum sample size of 28 participants.

### Experimental design

The task was based on a cheating game in which participants observed the outcome of a 6-sided die throw and had to report the result on a subsequent screen. Participants could either report the correct outcome (be honest) or could cheat by reporting a dishonest option. Each choice was associated with a payoff. To create a conflict between honesty and maximizing earnings, cheating was always the more profitable choice. Participants in our task performed a variation of this cheating game, in which we introduced two types of trials, Solo trials and Predict trials (Fig 2A and 2B).

#### Solo trials.

In these trials, participants played the cheating game described above. After a jittered inter-trial interval (ITI, 3–7 s), the outcome of the 6-sided dice roll was presented for 1 s, then participants had an unlimited time to choose which outcome to report, the left or the right one. The choice was made by pressing a button box with their right hand (either the right or the left button). The honest and dishonest choice sides were randomly determined for each trial. After the button press the screen froze for 0.5 s. The task involved no cue to confirm the decision, in order to maximize the participants’ perception of unaccountability [17].

#### Predict trials.

Predict trials commenced in a similar fashion to Solo trials because participants observed the outcome of a die roll and were presented with two options, an accurate and an inaccurate one. However, participants were told they had to predict the result that another individual reported in a previous experimental session. This other individual was described as being randomly selected, at the start of each new Predict trial, from a group of 10 participants who had completed the Solo trials in a previous experimental session. After indicating their prediction (left or right button press, depending on the predicted outcome), the predicted outcome was framed in red for a randomized duration (2–5 s). Then, participants received feedback during which the “real” outcome reported by the other was framed in green. If this frame was overlayed with the participants’ prediction, their prediction was correct, otherwise it was incorrect. A correct prediction was rewarded with €2 and €0 otherwise. The purpose of the predict trials was 2-fold. First, they allowed us to gather information about the learning process of our participants. Their predictions enabled us to estimate and select learning models that account for how they learn about the others’ cheating behavior. Second, they encouraged participants to actively learn the others’ behavior, because they were rewarded if their predictions were correct.

#### Groups simulations.

Unbeknownst to the participant, the group of 10 individuals were simulated. Specifically, we simulated two groups composed either of honest or dishonest individuals. We chose to tell to participants that they had to predict the decisions made by a group of individuals who performed the task in a previous experimental session to increase the likelihood of conformism towards the others’ behavior [55]. Furthermore, they were informed that the two groups of participants did not exhibit the same behavior. To maintain the behavior of the “group members” homogeneous within each group, we actually constructed a dataset based on the responses of two participants that performed the online pilot of the task. Specifically, we selected them based on the congruence of their behavior (no erratic behavior) and their cheating level. One cheated in 85% and the other in 20% of the Solo trials in the Baseline test. We then determined which utility functions best explained the pilot participants’ behavior, which was the variable cost of cheating utility function (see the section Utility functions for details). We then used the estimated parameters to simulate the cheating behavior of the group members in each Predict trial. One of the groups was based on the dishonest participant’s parameters and the other on the honest participant’s parameters. This procedure ensured a high level of credibility as the decisions were simulated from the true behavior of two participants. Furthermore, using a simulation allows us to add noise to the data to decrease the pace of learning by our participants. This gave us two sets of Predict trials, one in which “others” were cheating in 22% of the trials (Honest group), the other in which the “others” cheated in 88% of the trials (Dishonest group, see S1 Table for details).

#### Task sequence (Fig 2B).

The experiment was divided into three blocks and was inspired by a previous experiment on social influence affecting risky choices [6]. The first block, the Baseline, was composed of 50 Solo trials. The purpose of this block was to assess the initial preferences of the participants. The second and third blocks consisted of 50 Solo trials and 50 Predict trials that were interleaved starting with a Predict trial. The fact that participants were gradually exposed to the others’ (dis)honest behavior allowed us to test the potential link between learning about others’ behavior and the participants’ decisions to cheat. The difference between these two blocks was the cheating behavior of the group whose decisions the participants had to predict. One group was considered as dishonest (Dishonest Group condition, 88% cheating frequency) while the other was considered as honest (Honest Group condition, 22% cheating frequency). The order of presentation of the second and third blocks was randomized between participants. To prevent participants from simple imitative behavior, e.g., simply repeating the behavior from the previous Predict trials, the parameters (payoffs and dice scores) of any Predict trial were never the same as in any of the next three Solo trials.

#### Parametrization.

The dice values ranged from 1 to 6 and the payoffs ranged from €2 to €10 (€0 to €8) for the dishonest (honest) choices with €2 increments. Both the dice values and the payoffs were randomly selected with two constraints. First, we aimed to eliminate any correlation between the dice values and the payoffs. Consequently, specific dice values were not associated with fixed payoffs. Second, we aimed to have the same number of observations per value of relative earnings (difference between the payoff of the dishonest choice and the honest one). Based on the possible payoffs, 5 levels of relative reward for cheating were possible, €2, €4, €6, €8 or €10. We randomly selected 5 payoff combinations (payoffs for the honest and dishonest reports) for each level of relative earnings, leading to a set of 25 trials (each with 2 dice values and 2 payoffs). We then repeated this set twice for each block of the experiment, leading to a total of 50 trials whose order of presentation was randomized between participants.

### Procedure

The study took place at the Neuroimaging Center CERMEP (https://www.cermep.fr/cermep_en.php) and was approved by the local ethics committee (CPP Sud-Est 2, 2018−36). The study has been conducted according to the principles expressed in the Declaration of Helsinki. During the medical screening, participants provided written informed consent following the explanation of the experiments by a medical doctor. Before entering the fMRI scanner, participants were asked to read privately the instructions of the task (a translated version of the instructions is available in SI). A questionnaire was added at the end of the instructions to ensure they understood the task fully, and especially the fact that their decisions directly affected their final payment. We also made sure that participants were aware of the true anonymity of their decisions during the task. After the completion of the task, participants completed a debriefing questionnaire (a translated version of the questionnaire is available in SI). They had to state on a 5-point Likert scale (5 = “I completely believe it”, 1 = “I completely disbelieve it”) whether they believed that their decisions were kept confidential (mean = 4.42 ± 0.99), whether they believed their decisions were observed (mean = 3.39 ± 1.41) and whether they believed the other individuals were real (mean = 4.03 ± 1.02). These results confirmed that participants trusted that their decisions were confidential and that they believed that the group of others existed. To avoid a spillover effect on the cheating behavior in the task, we also asked participants, at the end of the task, whether they thought it was immoral to cheat (i.e., report the incorrect number) at the beginning of the task and at the end. The goal was to assess if the exposure to others’ dishonest behavior led to a change in their moral perception of cheating in our task. Ten participants reported, both for the beginning and end of the task, that it was never immoral to cheat, 10 reported that it was immoral at the beginning of the task but not immoral at the end, seven reported that it was immoral, both for the beginning and end of the task, three reported that they had no opinion, at the beginning of task, but thought that it was not immoral at the end of the task. One participant had no opinion both at the beginning and the end of the task. Finally, we asked them whether they had any comments or remarks about the task. We also explicitly told participants that the dishonest/honest behavior of the “other participants” were in fact generated by a computer simulation.

### Statistical analysis

#### Behavioral analysis.

Our behavioral statistical results are derived from mixed-effect logistic regressions. We always report the marginal effect rather than the odds ratio as it is easier to read and understand. The marginal effect can be interpreted as the mean discrete change of the dependent variable given a unitary change of an independent variable. In all regressions, we considered standard errors clustered at the participants’ level as well as a random effect at the participant level. When reporting pairwise comparisons of marginal effects, we always controlled for multiple comparisons using a Bonferroni correction. Other statistical tests are always two-sided nonparametric tests unless indicated otherwise.

#### Models.

Our social influence models are all based on two components: a decision model (i.e., utility function) that accounts for how participants decide to cheat or not, and a learning model, which describes how participants learned and predicted others’ cheating behavior. In this section, we first present the two utility functions considered in our models, followed by the six learning models. We then describe the four social influence models, which vary in how they integrate the utility function and learning models to explain social influence. Finally, we outline our model selection, model recovery, and parameter recovery procedures.

***Utility functions:*** Our different models are based on a core utility function derived from previous work in behavioral economics [34,35]. These models assume that agents compute a relative decision value of cheating by solving a cost-benefit arbitration between the relative gains of cheating and its moral cost (ΔDV (cheat)). Formally, the relative gains of cheating are weighted by a free parameter *α* representing the agent’s preference for money (with *α* > 0). The moral cost of cheating is a free parameter *δ* which is fixed (Fixed cost utility, see Eq. (1)). We also considered an alternative utility function in which the cost of cheating weights the payoff of cheating [25]) (Variable cost utility, see Eq. (2)).


$$\begin{array}{c} \Delta DV\;(cheat)\;=\;\alpha (\pi_{Cheat}-\pi_{Honest})\;+\;\delta \end{array}$$ (1)

$$\begin{array}{c} \Delta DV\;(cheat)\;=\;\alpha (\pi_{Cheat}-\pi_{Honest})\;+\;\delta \pi_{Cheat} \end{array}$$ (2)

***Learning models:*** To capture the computational processes underlying learning about others’ cheating behavior, we considered a family of 6 Bayesian learning models. All of them are based on the BPL model [36]. In these models, participants infer others’ preferences from others’ observed choices. Formally, others’ preferences correspond to their own parameters *α* and *δ* for their utility function with this set defined as Θ(o). This allows us to compute a relative decision value based on Eq. (1) or Eq. (2) which is then transformed to a probability using a softmax function with a temperature parameter *β*_Oth_ *(*with *β*_Oth_ > 0) that is treated as a free parameter. Participants are assumed to start with prior beliefs about the others’ parameters $p\left(\Theta^{\left(o\right)}\right)=N(\mu_{\text{0}}^{\left(o\right)},\sigma_{\text{0}}^{\left(o\right)})$ which are Gaussian with mean $\mu_{\text{0}}^{\left(o\right)}$and variance $\sigma_{\text{0}}^{\left(o\right)}$. Given the others’ choices, the participants estimate and update the set of parameters Θ(o) using the following Bayes-optimal probabilistic scheme:


$$\begin{array}{c} p(\Theta (o)|a_{t})\;\propto \;p(a_{t}|\Theta (o))p(\Theta (o)|a_{t-\text{1}}) \end{array}$$ (3)

where $p(\Theta (o)|a_{t})$ is the participant’s posterior belief about the other’s preference (here parameter *α*) at the end of trial *t* and the right part of *t*he equation represents the Bayesian belief update rule. Following [36], we used a variational-Laplace scheme to implement this model which yields $p\left(\Theta^{\left(o\right)}|a_{t}\right)\approx N(\mu_{\text{0}}^{\left(o\right)},\sigma_{\text{0}}^{\left(o\right)})$. For more details about the formal mathematical description, we refer the interested reader to the original paper [36]. As the BPL model assumes that participants start with priors about the others’ preferences, we considered 3 types of priors. In a first model, $\mu_{\alpha }$ and $\mu_{\delta }$ were equal to the value of *α* and *δ* of the participant’s own utility function, estimated in the Baseline (BPL-Self). According to this model, participants are assumed to consider the group members as individuals with preferences similar to their own (self-projection). In a second model, we set the priors as in the previous case, but only for the first group they faced (Dishonest or Honest group conditions). For the second group, $\mu_{\alpha }$ and $\mu_{\delta }$ were equal to $\mu_{\alpha }$ and $\mu_{\delta }$ learned at the last Predict trial of the first group they faced (BPL-Others). Here, participants are supposedly projecting the learned preferences of the first group onto the second one. In a third and last model, the setting was not changed for the first group, but only for the second, $\mu_{\alpha }$ and $\mu_{\delta }$ were the weighted means of the participant’s parameter values (as in the BPL-Self) and of the value learned for the previous group (BPL-SelfOthers). This weight is a free parameter ω.

Finally, we considered either one of the two utility functions to be the one used by our participants to infer the others’ preferences. In total, we tested the 3 different types of prior for each utility function (see Eqs. (1) and (2)) leaving us with 6 candidate models.

***Social influence models:*** To account for social influence our computational models are built on two potential mechanisms. The first one, the Preferences Shift (PS), assumes that participants’ preferences change when they are exposed to others’ cheating behavior [6,12]. The second one, the Valuation Bias (VB), assumes that participants’ decision values change, while their preferences remain constant, when they are exposed to others’ cheating behavior [3,4,18]. We derived formal computational models from these two mechanisms. For the Fixed Preferences Shift (PS_Fixed_) model this is formally defined as follows:


$$\begin{array}{c} \Delta DV\;(cheat{)}_{Influenced}\;=\;\alpha (B,D,H)(\pi_{Cheat}-\pi_{Honest})\;+\;\delta (B,D,H) \end{array}$$ (4)

In this equation (Eq. (4)), the participants’ preferences *α* and *δ* take different values for each condition (Baseline, Dishonest and Honest Groups: *B, D, H*).

For the Fixed Valuation Bias (VB_Fixed_) mechanism this is formally defined as follows:


$$\begin{array}{c} \Delta DV\;(cheat{)}_{Influenced}\;=\;\alpha (\pi_{Cheat}-\pi_{Honest})\;+\;\delta \;+\;\theta_{\left(D,H\right)} \end{array}$$ (5)

In this equation (Eq. (5)), the participants’ preferences are held fixed throughout the task.

A parameter *θ* represents the bias of the participants’ decision values due to the exposition to others’ cheating behavior. This parameter is different for the Dishonest Group ($\theta_{D}$) and for the Honest Group ($\theta_{H}$).

In addition to these two models, we also considered dynamic variants that imply that the preferences change or the value biases are affected by the participants’ knowledge of the others’ cheating behavior. The main idea is that, over observations of others’ behavior, our participants learn about the preferences of others’. Over the course of this learning, either the changes in our participants’ preferences or the valuation bias evolved as a result of their observations. Formally, we tested 2 different models that link the outcome of the BPL model with either the PS (Eq. (4)) or the VB model (Eq. (5)).

In the first model, we considered that the changes of preferences are the results of a weighted average of the participants’ own preferences and what they know at a given point in time about the others’ preferences. Formally, the Dynamic Preference Shift (PS_Dynamic_) model is defined as follows:


$$\begin{array}{c} \Delta DV\;(cheat{)}_{Influenced}\;=\;(\omega_{A}\alpha \;+\;\left(\text{1}\;-\;\omega_{A}\right)\alpha_{t}^{o})(\pi_{Cheat}-\pi_{Honest})+\;\left(\omega_{D}\delta \;+\;\left(\text{1}\;-\;\omega_{D}\right)\delta_{t}^{o}\right) \end{array}$$ (6)

where $\alpha_{t}^{o}$ and $\delta_{t}^{o}$ are the others’ preferences derived from the BPL model at time *t*, $\omega_{A}$ ($\omega_{D}$) being free parameters representing the magnitude of social influence. The closer to 0 these parameters are, *t*he more participants will be influenced by their perception of the others’ preferences, i.e., the more they will be susceptible to social influence (with $\text{0}{\leq \omega }_{A}(\omega_{D})\leq \text{1}$). Formally, they control the weight that participants put on their own preference parameter *α* (*δ*) and $\text{1}-\omega_{A}$ ($\text{1}-\omega_{D}$) and the weight that participants put on others’ learned preferences *α*_*t*_^*O*^ (*δ*_*t*_^*O*^). Both $\omega_{A}$ and $\omega_{D}$ which were estimated separately for the Dishonest and Honest Group conditions.

In the second model, we considered that the value bias is derived from what participants have learned about others’ preferences at a given point in time. Formally, the Dynamic Valuation Bias (VB_Dynamic_) model is defined as follows:


$$\begin{array}{c} \left\{ \begin{array}{c} @l\Delta DV\;(cheat{)}_{Influenced}\;=\;\alpha \left(\pi_{Cheat}-\pi_{Honest}\right)\;+\;\delta +\;\gamma P(Cheat{)}_{t}^{o\;}if\;Dishonest\;group\\ \Delta DV\;(cheat{)}_{Influenced}\;=\;\alpha \left(\pi_{Cheat}-\pi_{Honest}\right)\;+\;\delta -\;\gamma P(Honest{)}_{t}^{o\;}if\;Honest\;group \end{array}\right. \end{array}$$ (7)

$$\begin{array}{c} P{\left(Cheat\right)}_{t}^{o}=\frac{1}{\text{1}+e^{\Delta \text{DV}(\text{cheat}{)}_{Oth}}\;}\text{ and }P{\left(Honest\right)}_{t}^{o}=\text{1}-P{\left(Cheat\right)}_{t}^{o} \end{array}$$ (8)

where $P(Cheat{)}_{t}^{o\;}$ or ($P(Honest{)}_{t}^{O}$) is the dynamic valuation bias. It corresponds to the probability that others cheated (were honest) in the participant’s position. It is based on what participants learned about others’ preferences ($\alpha_{t}^{o}\text{ and }\delta_{t}^{o}$) at time *t* in each of the two group conditions and on the relative payoff of the given Solo trials. *γ* is a free parameter representing the exten*t* of the participants’ conformity. This last parameter is estimated separately for the Dishonest Group (*γ*_*D*_) and the Honest Group (*γ*_*H*_) conditions.

Overall, we tested 4 model candidates to account for social influence in our experiment. Participants were assumed to use the Fixed Moral cost utility function to compute their decision value as it is the utility function that best explained our participants’ behavior throughout the experiment (see S1B Fig and the Results section for details). Finally, we used a softmax function with an inverse temperature parameter (β>0) to transform the relative value of cheating into choice probability for each of the four models. A summary of all the free parameters of each social influence model is available in S9 Table.

***Model estimation and selection:*** Models were individually fitted using the Variational Bayes method with the VBA toolbox. Every prior was set to their default values, and we used the multisession option to allow cases in which free parameters were changing between conditions. Furthermore, in both the VB and PS Dynamics estimations, we disabled the update of the initial hidden state because they were based on the participants’ own parameters in the Baseline. All temperature parameters that govern choice stochasticity and social learning as well as parameter *α* were constrained to be strictly positive by estimating them in exponential form.

The Bayesian Model Selection (BMS) was performed using the VBA toolbox (Variational Bayesian Analysis) in a random effect analysis relying on the free energy as the lower bound of model evidence. We used protected Exceedance Probability measurements (pEP) to select the model used most frequently in our population of participants [33].

***Model and parameters recovery:*** To check that our models make distinct predictions on behavior, we generated 20 datasets for each of our 28 participants independently (total of 560 datasets). We randomly drew free parameters from a normal distribution centered on the mean of the estimated parameters of the participants, and with its variance being the standard deviation of the participants’ parameters. We then estimated each model on each dataset using the same procedure as for our main estimation. This procedure was applied to the 4 social influence models (PS_fixed_, VB_fixed_, PS_dynamic_, VB_dynamic_) and the two utility functions (fixed and variable moral cost). The results of these model recovery analyses are shown in S2 Fig. We found that no other model produced behavior that could be confounded with the winning model (VB_Dynamic model_; S2A and S2B Fig) and that our experimental settings allowed the two types of utility function to be correctly distinguished (S2C and S2D Fig).

To check whether our estimated parameters were identifiable, we generated 100 datasets for each of our 28 participants independently (total of 2,800 datasets) for both the VB_Dynamic_ model and the Fixed Moral Cost utility model. We drew free parameters randomly from a normal distribution centered on the mean of the estimated parameters of the participants and with its variance being the standard deviation of the participants’ parameters. We then estimated the free parameters recovered when fitting the VB_Dynamic_ model and the Fixed Moral Cost utility model on these new datasets. We performed linear regressions on each recovered parameter with the generative parameter and calculated the correlation coefficient ρ. Results (S3 Fig) indicate that most recovered parameters were strongly correlated with the generative free parameter. For the VB_Dynamic_ model (S3A Fig), only the temperature used to compute the others’ choice ($\beta_{\text{Oth}}$) showed poor recovery. This might be driven by the participants learning the behavior of the others very rapidly. Note that our results are mostly based on the conformity parameter *γ* which is well recovered. For the parameter recovery of the Fixed Moral cost utility model (S3B Fig), we found that the recovery of the moral cost *δ* and the temperature *β* were relatively weak. However, the recovery of these two parameters was good in the VB_Dynamic_ model that included the fixed cost utility model and which is the actual model we applied for our analysis.

#### fMRI acquisition.

MRI acquisitions were performed on a 3 Tesla scanner using EPI BOLD sequences and T1 sequences at high resolution. Scans were performed in a Siemens Magnetom Prisma scanner HealthCare at CERMEP Bron (single-shot EPI, TR/TE = 1,600/30, flip angle 75, multiband acquisition (accelerator factor of 2), in an ascending interleaved manner with slices interlaced 2.40 mm thickness, FOV = 210 mm. We also used the iPAT mode with an accelerator factor of 2 and the GRAPPA method reconstruction. The number of volumes acquired varied given the time the participant took to make their decisions. The first acquisition was made after stabilization of the signal (3 TR). Whole-brain high-resolution T1-weighted structural scans (0.8 × 0.8 × 0.8 mm) were acquired for each subject, co-registered with their mean EPI images and averaged across subjects to permit anatomical localization of functional activations at the group level. Field map scans were acquired to obtain magnetization values that were used to correct for field inhomogeneity.

#### fMRI preprocessing and data analysis.

Image analysis was performed using SPM12 (Wellcome Department of Imaging Neuroscience, Institute of Neurology, London, UK, fil.ion.ucl.ac.uk/spm/software/spm12/). Time-series images were registered in a 3D space to minimize any effect that could result from participant head motion. Once DICOMs were imported, functional scans were realigned to the first volume, corrected for slice timing and unwarped to correct for geometric distortions. Inhomogeneous distortions-related correction maps were created using the phase of non-EPI gradient echo images measured at two echo times (5.20 ms for the first echo and 7.66 ms for the second). Finally, in order to perform group and individual comparisons, they were co-registered with structural maps and spatially normalized into the standard Montreal Neurological Institute (MNI) atlas space using the DARTEL method with a spatial smoothing using an 8-mm full-width at half-maximum Gaussian kernel.

We ran general linear model (GLM) analyses to identify brain regions that encode the following computational signals: (1) the dynamic valuation bias in both the Dishonest and Honest Group conditions (choice stage in the Solo trials); (2 the probability associated with participants’ predictions, in the Predict trials in both the Dishonest and Honest group conditions (prediction stage in the Predict trials); (3) the relative value of participants’ chosen options in Solo trials, regardless of conditions. We also included a fourth signal, the prediction error at the feedback stage of the Predict trials. In every GLM an event was defined as a boxcar function whose duration was equal to the participant’s reaction time or to the time of display with the exception of the button press which was defined as a stick function. Events such as the stimulus stage, the choice and prediction stages for both Solo and Predict trials as well as the feedback stage in the Predict trials were always included in all of our GLMs. Head movement parameters were added as parametric regressors of no interest to account for motion-related noise. Finally, to control for diverse task parameters, we added as parametric regressors in every GLM for the choice and prediction onsets: the choice or prediction of the participant (0: No cheat, 1: Cheat), the participants’ reaction time, and which side was pressed in the choice or prediction stage of the Solo and Predict trials, respectively. All of our parametric regressors were orthogonalized due to strong correlation between the parametric regressors of interest and the model’s parameters. Based on these common features we defined 2 GLMs.

In GLM1, we added the following parametric regressor: the inferred others’ probability to cheat (be honest) in the choice stage of the Solo trials of the Dishonest (Honest) group condition (dynamic valuation bias, signal 1). In GLM2, we added the following parametric regressors: the probability associated with participants’ predictions at the prediction stage of Predict trials in both the Dishonest and Honest Group conditions (signal 2). In the choice stage of the Solo trials, we added the relative value of the participants’ choice in every condition (signal 3). Then, in the feedback stage of the Predict trials we added the prediction error from the BPL-Self model in both the Dishonest and Honest Group conditions. Finally, in both GLMs, we added the participants’ conformity parameter γD or γH as a second-level covariate, for the Dishonest and Honest Group conditions, respectively.

We computed one-sample *t*-tests with contrasts for main effect for each parametric regressor as well as the main effect per block (Baseline, Dishonest and Honest groups) when possible. Reported brain areas show a significant activity at the threshold of *p <* 0*.*05, whole brain family-wise error (FWE), corrected for multiple comparisons at the cluster level (threshold at *p <* 0*.*001 uncorrected).

## Supporting information

S1 Text Supplementary results. Instructions (translated from French).
(DOCX)

S1 Fig Utility function and social learning model selection. **A**. Estimated model frequencies (Ef) of our 4 candidate models. Black bars show the probability that each model explains the participants’ behavior independently of the other models. **B.** Protected Exceedance Probability (pEP) of the different utility functions associated with cheating, estimated over the three blocks of the experiment. The higher the pEP the more likely a given model explains the group’s behavior better than the others. The fixed cost only utility function assumes that participants are incurring a fixed moral cost when choosing to cheat, in contrast, the variable cost variant assumes that this moral cost depends on the cheating payoff. The mixed variants are all the possible combinations of these two utility functions across the three blocks of the experiment (baseline and group conditions). **C.** Protected Exceedance Probability (pEP) of the different social learning models. The higher the pEP the more likely a given model explains the group’s behavior better than the others. We tested a total of 6 models, all based on the Bayesian Preference Learning model (BPL), for which we varied the priors concerning the others’ parameters (Self, Others or SelfOthers) and whether the utility function that the participants used to learn about the others’ cheating behavior was the fixed cost or the variable cost utility function. **D.** Protected Exceedance Probability (pEP) of the social influence models, which included two variants of the Preference Shift Fixed model. The variant *α* is the same as in the original model except that the parameter *α* does not change across conditions (PS_fixed (*α*)_). Similarly, the variant δ assumes that *δ* does not change across conditions (PS_fixed (*δ*)_). The data underlying the figure can be found in the Figures folder of the OSF repository.
(TIF)

S2 Fig Model recovery analysis. We generated data with free parameters randomly selected from a normal distribution centered on the mean of all participants’ free parameters, and with its variance being the standard deviation of the participants’ individual parameters. Using this procedure, 20 datasets for each of our 28 participants were generated (total of 560 datasets). Then, we ran a model selection on these generated datasets, and repeated this procedure for each model. The rows are the generative models. The columns are the fitted model. **A and C**. The colors represent the mean probability that one model is more frequent than another (in our population of generated datasets), given a Bayesian model selection (Exceedance probability) for the four social influence models (A) and the two utility functions considered in our analysis (C). **B and D**. The colors represent the mean frequency of each model being the best model to explain the simulations. We assessed which model was the best for each simulation based on which one has the highest Exceedance probability. We followed this procedure for the four social influence models (B) and the two utility functions considered in our analysis (D). The data underlying the figure can be found in the Figures folder of the OSF repository.
(TIF)

S3 FigParameter recovery analysis.**A**. Simulated and recovered parameters of the VB_Dynamic_ model. The black line represents the linear regression linking the estimated parameters based on simulated choices. *ρ* is the correlation coefficient. The transparent points represent the 5% of simulations that are farthest from the regression line. The value of *ρ* in parentheses is the correlation coefficient computed from the remaining simulations. Only the simulated temperature parameter for the other show inconsistency. $\;\gamma_{D},\gamma_{H}$ represent the parameter γ estimated for the Dishonest and Honest Group conditions, respectively. **B.** Simulated and recovered parameters of the Fixed cost utility model. The black line represents the linear regression curve linking the estimated parameters based on simulated choices. *ρ* is the correlation coefficient. The transparent points represent the 5% of simulations with the poorest fit. The value of *ρ* in parentheses is the correlation coefficient computed from the remaining simulations. The data underlying the figure can be found in the Figures folder of the OSF repository.(TIF)

S4 Fig Parameter estimation. **A**. Violin plot of participants’ estimated VB_Dynamic_ model parameters *α*_Self_, *δ*_Self_, *β*_Self_ and *β*_Other_. Stars indicate the result of Wilcoxon signed-rank tests assessing whether parameter distributions differed from zero. **B.** Violin plots of the estimated conformity parameters iiii and *γ*_*H*_. Bottom stars indicate the result of Wilcoxon signed-rank tests assessing whether each parameter differed from zero. Top stars correspond to the *p*-values of a two-sided the result of Wilcoxon rank-sum test comparing the parameters value between Group condition (*γ*_*D*_ for the Dishonest Group and *γ*_*H*_ for the Honest Group). **C and D.** Violin plots of the learned parameters *α* (C) and *δ* (D) for both the Dishonest and Honest Group conditions. Bottom stars correspond to the *p*-values of a Wilcoxon signed rank test assessing whether each parameter differed from zero. Top stars correspond to the *p*-values of a two-sided the result of Wilcoxon rank-sum test comparing the parameters value between Group condition. **E.** Correlation matrix between fitted parameters. Stars indicate significant correlations. For the whole figure: *** *p* < 0.001, ** *p* < 0.01, * *p* < 0.05, n.s. *p* ≥ 0.05. The data underlying the figure can be found in the Figures folder of the OSF repository.
(TIF)

S5 Fig A. Mean simulated cheating as a function of *γ*. We simulated behavior while fixing all parameters to each participant’s estimated values and varying only *γ* from −1 to 1.5 (by step of 0.1). Higher *γ* values shifted simulated behavior toward the group norm: increased cheating in the Dishonest Group condition and reduced cheating in the Honest Group condition. **B.** Correlation between fitted γ parameters and the difference between the participants’ mean cheating in each Group condition and their mean cheating in the baseline. The direction of the correlations is consistent with the interpretation of *γ* as a conformity parameter, although the associations were weak. The values *ρ* are the correlation parameters obtained from a Pearson correlation (*p* = 0.105 and *p* = 0.010 for the Dishonest Group and Honest Group conditions, respectively). **C.** The participants’ moral cost parameter δ is not correlated with the participants’ conformity parameter *γ* in either the Dishonest or Honest Group conditions. The values *ρ* are the correlation parameters obtained from a Pearson correlation (*p* = 0.689 and *p* = 0.114 for the Dishonest Group and Honest Group conditions, respectively). **D.** The participants’ average prediction accuracy is not correlated with the participants’ absolute conformity parameter *γ* in either the Dishonest or Honest Group conditions. The values of *ρ* are the correlation parameters obtained from a Pearson correlation (*p* = 0.201 and *p* = 0.659 for the Dishonest and Honest Group conditions, respectively). **E.** The participants’ average prediction accuracy difference between the two Group conditions is not correlated with the difference in conformity between the two Group conditions, which indicates that asymmetries in learning accuracy cannot explain asymmetries in conformity. The value *ρ* = 0.006 is the correlation parameter obtained from a Pearson correlation between the two and it is not significant (*p* = 0.975). The data underlying the figure can be found in the Figures folder of the OSF repository.
(TIF)

S6 Fig A. Individual beta estimates extracted from a bilateral pSTS-TPJ ROI [26] in the Dishonest Group condition (y-axis) and in the Honest Group condition (x-axis). The ROI coordinates are [−55 −45 13] (left side) and [55 −45 13] (right side). **B**. Regions encoding the prediction error from the social learning model at the time of the feedback in the Predict trials (*p* < 0.001 uncorrected and *p* < 0.05 whole-brain cluster-corrected family-wise error (FWE)).
(TIF)

S1 Table Mean cheating frequency per level of relative pay for cheating for the two groups and model parameters used to simulate the groups’ behavior (Variable Moral cost utility function). (DOCX)

S2 Table Logistic random-effect regressions. *Notes*: Relative payoff for cheating: *π*_Cheat_ − *π*_Honest_, Diff. dice value: Dice value_Cheat_ − Dice value_Honest_. Standard errors clustered at the participant level are in parentheses. *** *p* < 0.001, ** *p* < 0.01, * *p* < 0.05. The data underlying the table can be found in the Tables folder of the OSF repository.
(DOCX)

S3 Table Additional logistic random-effect regressions. *Notes*: Relative payoff for cheating: *π*_Cheat_ − *π*_Honest_, Diff. dice value: Dice value_Cheat_ − Dice value_Honest_. Standard errors clustered at the participant level are in parentheses. *** *p* < 0.001, ** *p* < 0.01, * *p* < 0.05. The data underlying the table can be found in the Tables folder of the OSF repository.
(DOCX)

S4 Table Logistic random-effect regressions. Effect of a change in beliefs about the morality of cheating in the task. *Notes*: Standard errors clustered at the participant level are in parentheses. *** *p* < 0.001, ** *p* < 0.01, * *p* < 0.05. The data underlying the table can be found in the Tables folder of the OSF repository.
(DOCX)

S5 Table Brain regions encoding the Dynamic Valuation Bias in the Solo trials of the Dishonest Group condition modulated by the participants’ conformity parameter *γ.* *Notes:* cluster reported at *p <* 0.05 FWE whole brain cluster corrected (initial cluster-forming threshold of *p <* 0.001 uncorrected).
(DOCX)

S6 Table Brain regions encoding the probability of the participants’ prediction at the time of the prediction in the Predict trials of the Dishonest Group condition. *Notes:* cluster reported at *p <* 0.05 FWE whole brain cluster corrected (initial cluster-forming threshold of *p <* 0.001 uncorrected).
(DOCX)

S7 Table Brain regions encoding the probability of the participants’ prediction at the time of the prediction in the Predict trials. Contrast between the two Group conditions. *Notes:* cluster reported at *p <* 0.05 FWE whole brain cluster corrected (initial cluster-forming threshold of *p <* 0.001 uncorrected).
(DOCX)

S8 Table Brain regions encoding the prediction error at the time of the feedback in the Predict trials. *Notes:* cluster reported at *p <* 0.05 FWE whole brain cluster corrected (initial cluster-forming threshold of *p <* 0.001 uncorrected).
(DOCX)

S9 Table Summary of the free parameters of each social influence model. B, D and H mean that the given parameter is estimated separately for the Baseline (B), the Dishonest Group condition (D) and the Honest Group condition (H).
(DOCX)

## Abbreviations

BMS: Bayesian Model Selection
BPL: Bayesian Preference Learning
dACC: dorsal anterior cingulate cortex
dlPFC: dorsolateral prefrontal cortex
fMRI: functional magnetic resonance imaging
FWE: family-wise error
GLM: general linear model
lPFC: Lateral Prefrontal Cortex
IPS: Inferior Parietal Sulcus
ITI: inter-trial interval
MNI: Montreal Neurological Institute
pEP: protected exceedance probability
PS: Preferences Shift
pSTS: posterior superior temporal sulcus
TPJ: Temporo-Parietal Junction
rdlPFC: right dorsolateral Prefrontal Cortex
rTPJ: right temporo-parietal junction
VB: Valuation Bias
