# Supplementary material for: Social learning dynamically shapes moral decision-making by biasing subjective valuation
Source: PLoS Biol. 2026 Jul 10;24(7):e3003889. doi: 10.1371/journal.pbio.3003889 (PMC13379141; doi:10.1371/journal.pbio.3003889)
Supplement: S1 Text — Instructions (translated from French). (DOCX) [file pbio.3003889.s001.docx]

Instructions

*Translated from French*

Thank you for participating in this decision-making experiment. In this experiment, all your decisions are anonymous. Your choices will not be associated with your name or other identifying information. Furthermore, the person supervising the experimental sessions is not the same as the one who will process the data.

Unlike most experiments, your decisions will determine your final payout. In addition to these earnings, you'll also receive a fixed amount of 50 Euros. The other participants won't know how much you've earned. The experiment consists of a series of throws of a 6-sided die. What you do next depends on the type of trial you are doing. There are two types of trials: Solo and Prediction. We'll explain them one after the other.

**Solo trials**

In each Solo trial, you'll see the result of a 6-sided die throw for 1 second. You need to memorize the number thrown, because you'll be asked to report it on the next screen. On the report screen, two numbers will be displayed, one on the right and one on the left of the screen. If you think the number on the right is the one thrown previously on the dice then press the right button on the response pad. Conversely, if you think the number on the left is the one that was thrown, then press the left button on the response pad. Finally, Solo trials are indicated by a padlock at the top of the screen.

As previously mentioned, your payoff in each Solo period will depend on the number you report. The amount, in Euros, associated with each of the two numbers you can report is displayed below each one. This amount does not depend on the number on the die.

**Predict periods**

The start of each Predict trial is the same as the Solo trial. You will see the result of a 6-sided die throw for 1 second. However, instead of reporting the result of this throw, you have to predict what was reported by a person, randomly selected from a group of 10 people who did this as a solo trial previously. All the members of that group made the same types of decisions as you in the Solo periods. They have never been informed of the other people's decisions. In every Predict trial, a new person is selected at random from the group.

After you've seen the die roll result, you will be shown the same decision screen as the person from the group. That is, the two dice roll numbers and the amounts of money displayed on the screen. You have to guess which number that person reported, and indicate it by pressing the button that corresponds to that number. Press the Right button for the number on the right, or the Left button for the number on the left. When your answer is validated, the number you've indicated will be framed in red. Then the number actually reported by the person will be framed in green. If your guess is correct, the number you've chosen will be framed in both red and green, as in the example below.


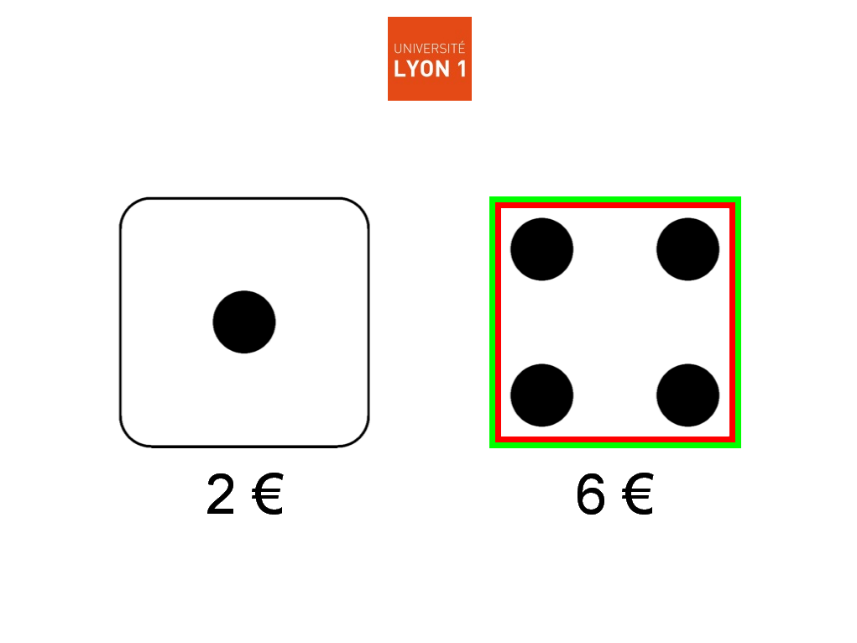


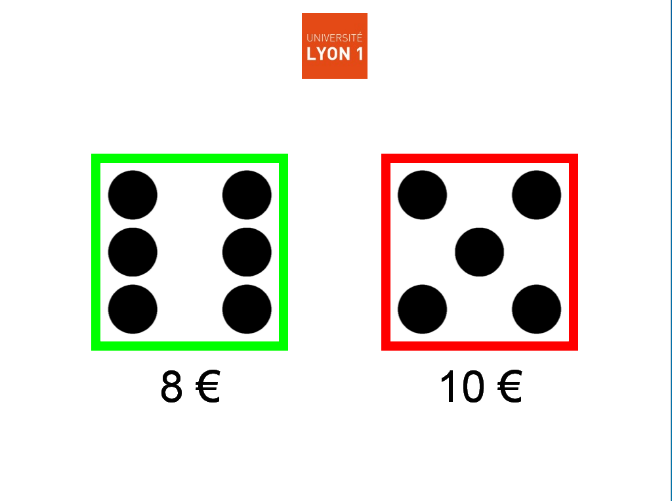
 If your prediction is incorrect, the number you chose will be framed in red and the number actually reported by the person will be in green, as in the example below.

If your guess is wrong, you will receive 0 Euros. If your prediction is correct, you will receive 2 Euros. Finally, each prediction period is indicated by the following picture:


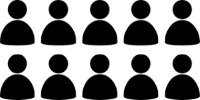


**Experiment sequence**

The experiment takes place in 3 stages:

You will start by doing a few trials to familiarize yourself with the task. The decisions you make during this training period will not be taken into account, either for the analyses or for your payment. Furthermore, the parameters for these trials are fictitious.

Then the experiment begins, consisting of three blocks:

- The first block consists solely of 50 Solo trials.
- The second block is made up of both Predict and Solo trials: 50 Predict trials and 50 Solo trials, presented in alternating order. You'll start with a Predict trial, then a Solo trial, followed by a Predict trial, and so on.
- The third and final block is organized the same as the second block.

**Group composition**

As mentioned above, the second and third blocks are similar in their organization. However, the group of 10 people from which the person whose decision you have to predict was selected is not the same for the two blocks. In the second block you will predict and observe the decisions of Group 1, and in the third block you will predict and observe the decisions of Group 2.

The people in these two groups completed the same task as you are doing in a previous online study. They only completed Block 1 of the experiment. They therefore received no information about other people's decisions.

We selected them and separated them into two groups according to their behavior. Each individual therefore behaved very similarly to the other members of their group. They performed the task online during the first Covid lockdown. They were mainly recruited via the Institute of Cognitive Sciences Facebook page and were all aged between 18 and 35. For reasons of anonymity, we can't give you any further information about the individuals in these two groups.

**Payment**

Two trials of the Solo condition and two trials of the Predict condition will be randomly selected at the end of the three blocks. For the Solo trials selected, you will be paid the amount in Euros associated with the number you reported. For the selected Predict trials, if your guess was correct, you will receive 2 Euros, otherwise you will not receive any additional reward.

Finally, you will receive 50 Euro for your participation in this experiment, regardless of your decisions. Your earnings will be paid to you by bank transfer.

**Other information**

Remember: since you don't know which trials will be selected at random, your best strategy is to make each decision as if it was one that really counts. All your decisions are final and cannot be changed.

Finally, the people organizing the experiment will never know your secret decisions. Furthermore, because your decisions are secret, your answers are not monitored. Nor will they be used for other participants' Predict periods.

**Debriefing questionnaire**

*Translated from French*

For each of the following questions, indicate your answer using the scale provided. There are no right or wrong answers. Please indicate what you really think.

1. My decisions were kept confidential.

I completely disbelieve it ☐ ☐ ☐ ☐ ☐ I completely believe it

2. My decisions were observed.

I completely disbelieve it ☐ ☐ ☐ ☐ ☐ I completely believe it

3. Were the decisions of the other participants real?

I completely disbelieve it ☐ ☐ ☐ ☐ ☐ I completely believe it

4. At the start of the task, did you think it was morally wrong to report a number other than the correct number?

☐ Yes ☐ No ☐ No opinion

6. Now, do you think it's morally wrong to report a number other than the real number?

☐ Yes ☐ No ☐ No opinion
