# Supplementary figures and images for: Social learning dynamically shapes moral decision-making by biasing subjective valuation
Source: PLoS Biol. 2026 Jul 10;24(7):e3003889. doi: 10.1371/journal.pbio.3003889 (PMC13379141; doi:10.1371/journal.pbio.3003889)

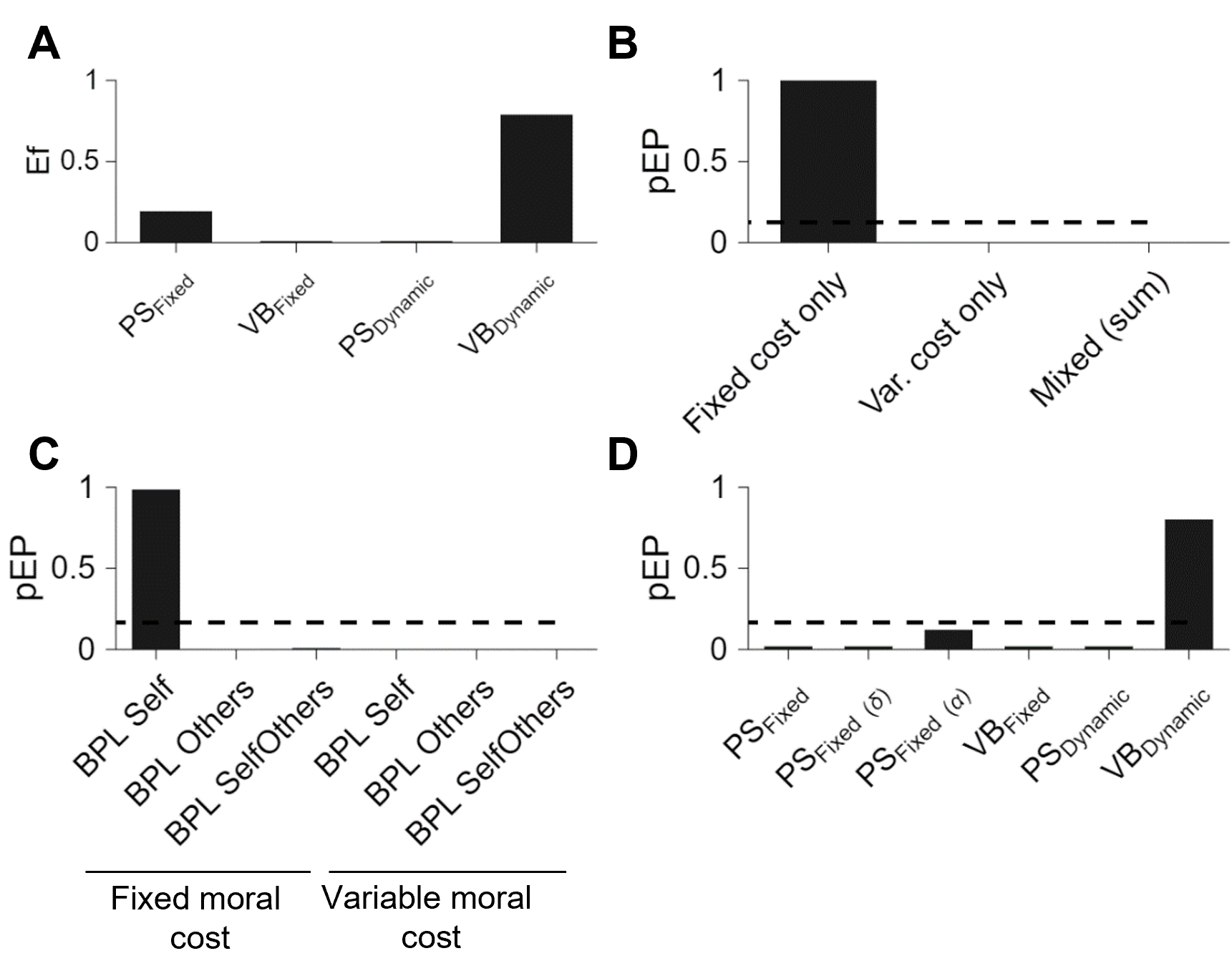

Supplement: S1 Fig — A. Estimated model frequencies (Ef) of our 4 candidate models. Black bars show the probability that each model explains the participants’ behavior independently of the other models. B. Protected Exceedance Probability (pEP) of the different utility functions associated with cheating, estimated over the three blocks of the experiment. The higher the pEP the more likely a given model explains the group’s behavior better than the others. The fixed cost only utility function assumes that participants are incurring a fixed moral cost when choosing to cheat, in contrast, the variable cost variant assumes that this moral cost depends on the cheating payoff. The mixed variants are all the possible combinations of these two utility functions across the three blocks of the experiment (baseline and group conditions). C. Protected Exceedance Probability (pEP) of the different social learning models. The higher the pEP the more likely a given model explains the group’s behavior better than the others. We tested a total of 6 models, all based on the Bayesian Preference Learning model (BPL), for which we varied the priors concerning the others’ parameters (Self, Others or SelfOthers) and whether the utility function that the participants used to learn about the others’ cheating behavior was the fixed cost or the variable cost utility function. D. Protected Exceedance Probability (pEP) of the social influence models, which included two variants of the Preference Shift Fixed model. The variant α is the same as in the original model except that the parameter α does not change across conditions (PSfixed (α)). Similarly, the variant δ assumes that δ does not change across conditions (PSfixed (δ)). The data underlying the figure can be found in the Figures folder of the OSF repository. (TIF) [file pbio.3003889.s002.tif]

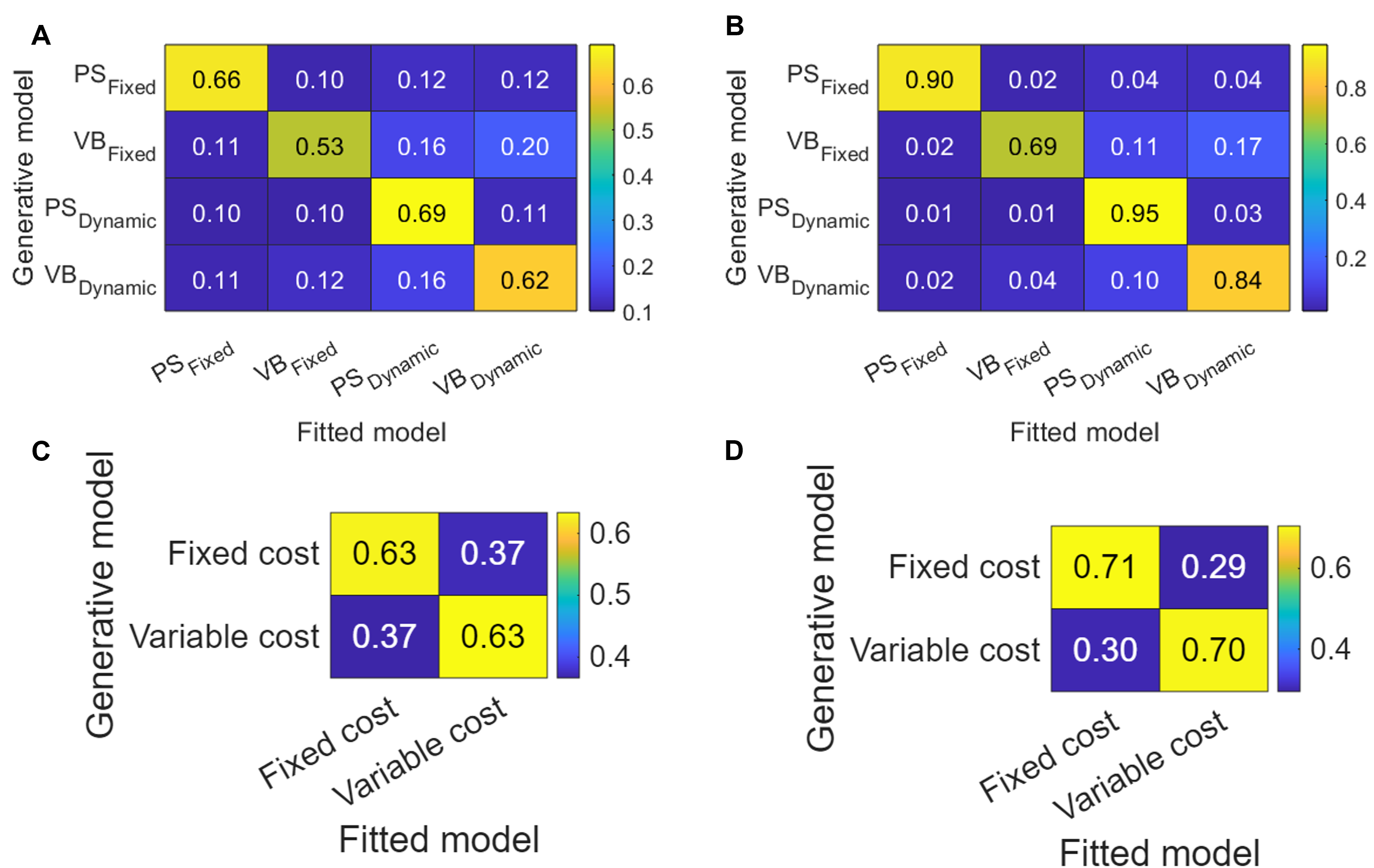

Supplement: S2 Fig — We generated data with free parameters randomly selected from a normal distribution centered on the mean of all participants’ free parameters, and with its variance being the standard deviation of the participants’ individual parameters. Using this procedure, 20 datasets for each of our 28 participants were generated (total of 560 datasets). Then, we ran a model selection on these generated datasets, and repeated this procedure for each model. The rows are the generative models. The columns are the fitted model. A and C. The colors represent the mean probability that one model is more frequent than another (in our population of generated datasets), given a Bayesian model selection (Exceedance probability) for the four social influence models (A) and the two utility functions considered in our analysis (C). B and D. The colors represent the mean frequency of each model being the best model to explain the simulations. We assessed which model was the best for each simulation based on which one has the highest Exceedance probability. We followed this procedure for the four social influence models (B) and the two utility functions considered in our analysis (D). The data underlying the figure can be found in the Figures folder of the OSF repository. (TIF) [file pbio.3003889.s003.tif]

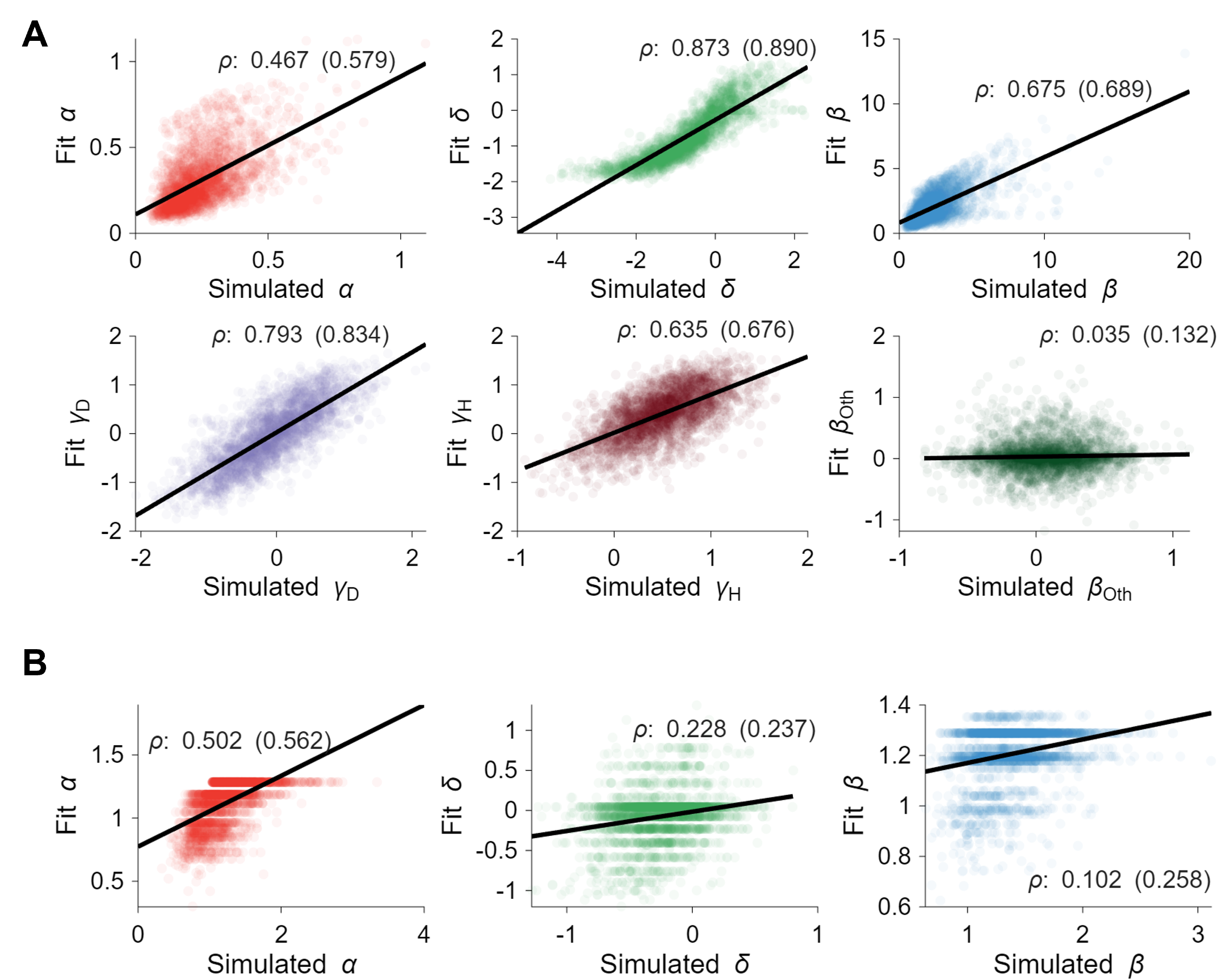

Supplement: S3 Fig — A. Simulated and recovered parameters of the VBDynamic model. The black line represents the linear regression linking the estimated parameters based on simulated choices. ρ is the correlation coefficient. The transparent points represent the 5% of simulations that are farthest from the regression line. The value of ρ in parentheses is the correlation coefficient computed from the remaining simulations. Only the simulated temperature parameter for the other show inconsistency. γD,γH represent the parameter γ estimated for the Dishonest and Honest Group conditions, respectively. B. Simulated and recovered parameters of the Fixed cost utility model. The black line represents the linear regression curve linking the estimated parameters based on simulated choices. ρ is the correlation coefficient. The transparent points represent the 5% of simulations with the poorest fit. The value of ρ in parentheses is the correlation coefficient computed from the remaining simulations. The data underlying the figure can be found in the Figures folder of the OSF repository. (TIF) [file pbio.3003889.s004.tif]

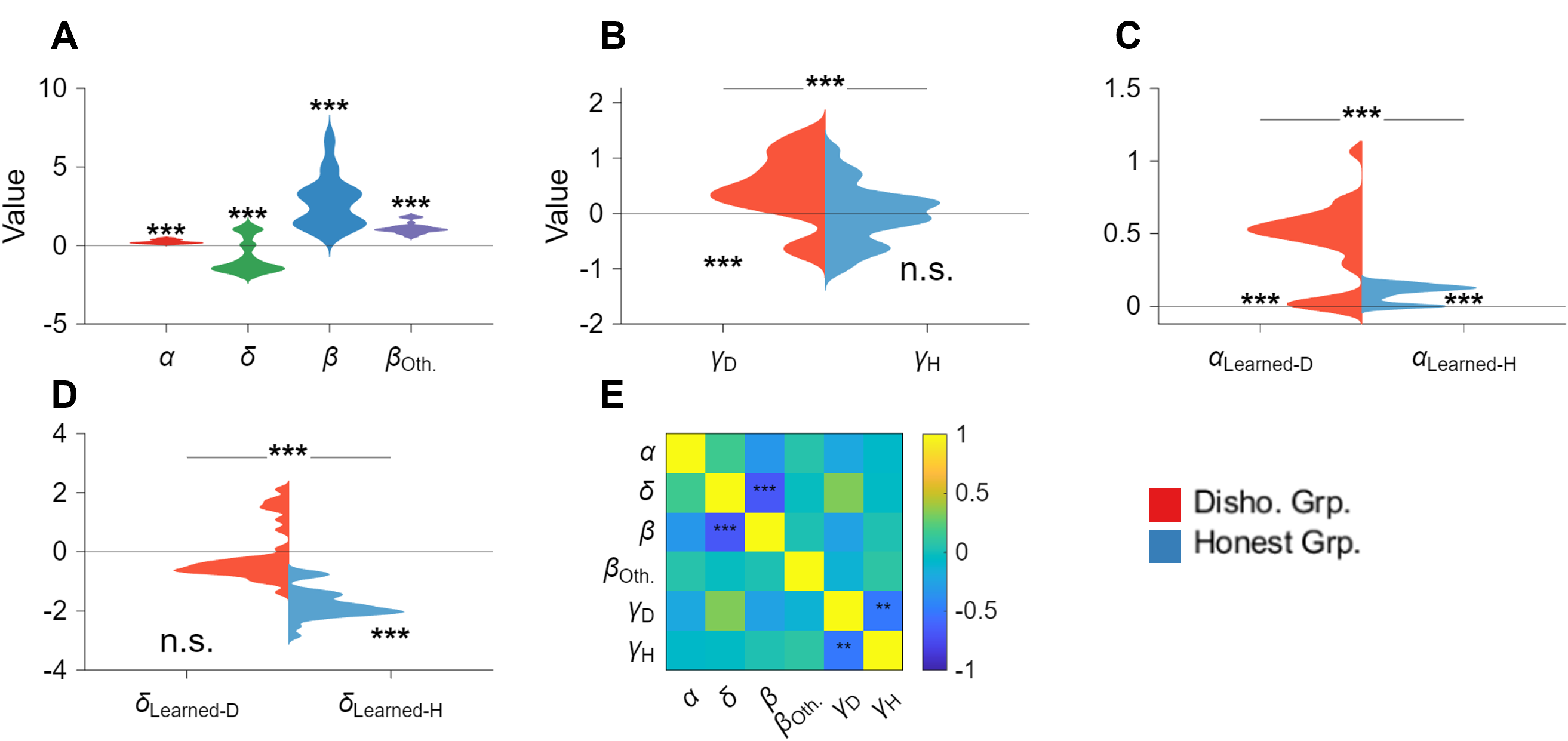

Supplement: S4 Fig — A. Violin plot of participants’ estimated VBDynamic model parameters αSelf, δSelf, βSelf and βOther. Stars indicate the result of Wilcoxon signed-rank tests assessing whether parameter distributions differed from zero. B. Violin plots of the estimated conformity parameters iiii and γH. Bottom stars indicate the result of Wilcoxon signed-rank tests assessing whether each parameter differed from zero. Top stars correspond to the p-values of a two-sided the result of Wilcoxon rank-sum test comparing the parameters value between Group condition (γD for the Dishonest Group and γH for the Honest Group). C and D. Violin plots of the learned parameters α (C) and δ (D) for both the Dishonest and Honest Group conditions. Bottom stars correspond to the p-values of a Wilcoxon signed rank test assessing whether each parameter differed from zero. Top stars correspond to the p-values of a two-sided the result of Wilcoxon rank-sum test comparing the parameters value between Group condition. E. Correlation matrix between fitted parameters. Stars indicate significant correlations. For the whole figure: *** p < 0.001, ** p < 0.01, * p < 0.05, n.s. p ≥ 0.05. The data underlying the figure can be found in the Figures folder of the OSF repository. (TIF) [file pbio.3003889.s005.tif]

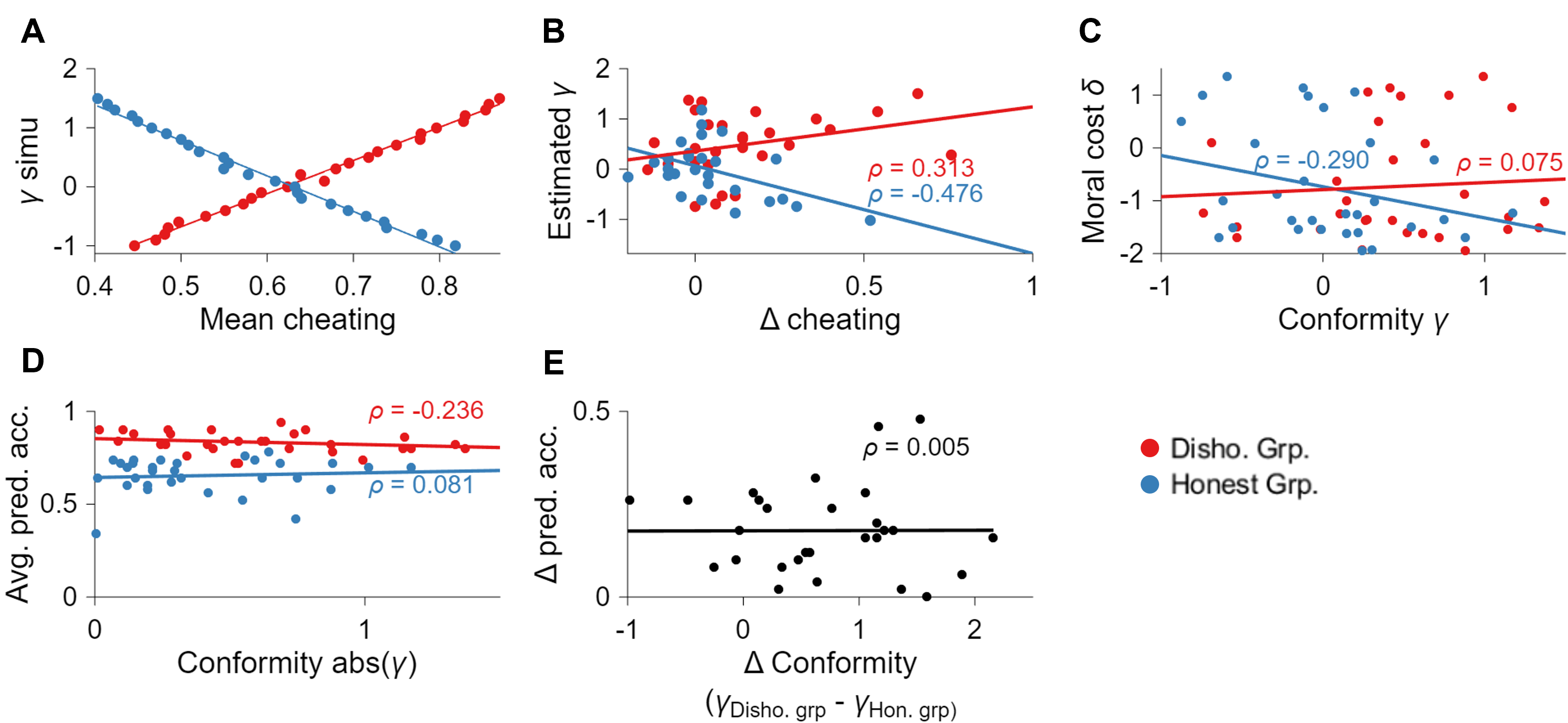

Supplement: S5 Fig — Mean simulated cheating as a function of γ. We simulated behavior while fixing all parameters to each participant’s estimated values and varying only γ from −1 to 1.5 (by step of 0.1). Higher γ values shifted simulated behavior toward the group norm: increased cheating in the Dishonest Group condition and reduced cheating in the Honest Group condition. B. Correlation between fitted γ parameters and the difference between the participants’ mean cheating in each Group condition and their mean cheating in the baseline. The direction of the correlations is consistent with the interpretation of γ as a conformity parameter, although the associations were weak. The values ρ are the correlation parameters obtained from a Pearson correlation (p = 0.105 and p = 0.010 for the Dishonest Group and Honest Group conditions, respectively). C. The participants’ moral cost parameter δ is not correlated with the participants’ conformity parameter γ in either the Dishonest or Honest Group conditions. The values ρ are the correlation parameters obtained from a Pearson correlation (p = 0.689 and p = 0.114 for the Dishonest Group and Honest Group conditions, respectively). D. The participants’ average prediction accuracy is not correlated with the participants’ absolute conformity parameter γ in either the Dishonest or Honest Group conditions. The values of ρ are the correlation parameters obtained from a Pearson correlation (p = 0.201 and p = 0.659 for the Dishonest and Honest Group conditions, respectively). E. The participants’ average prediction accuracy difference between the two Group conditions is not correlated with the difference in conformity between the two Group conditions, which indicates that asymmetries in learning accuracy cannot explain asymmetries in conformity. The value ρ = 0.006 is the correlation parameter obtained from a Pearson correlation between the two and it is not significant (p = 0.975). The data underlying the figure can be found in the Figures folder of the [file pbio.3003889.s006.tif]

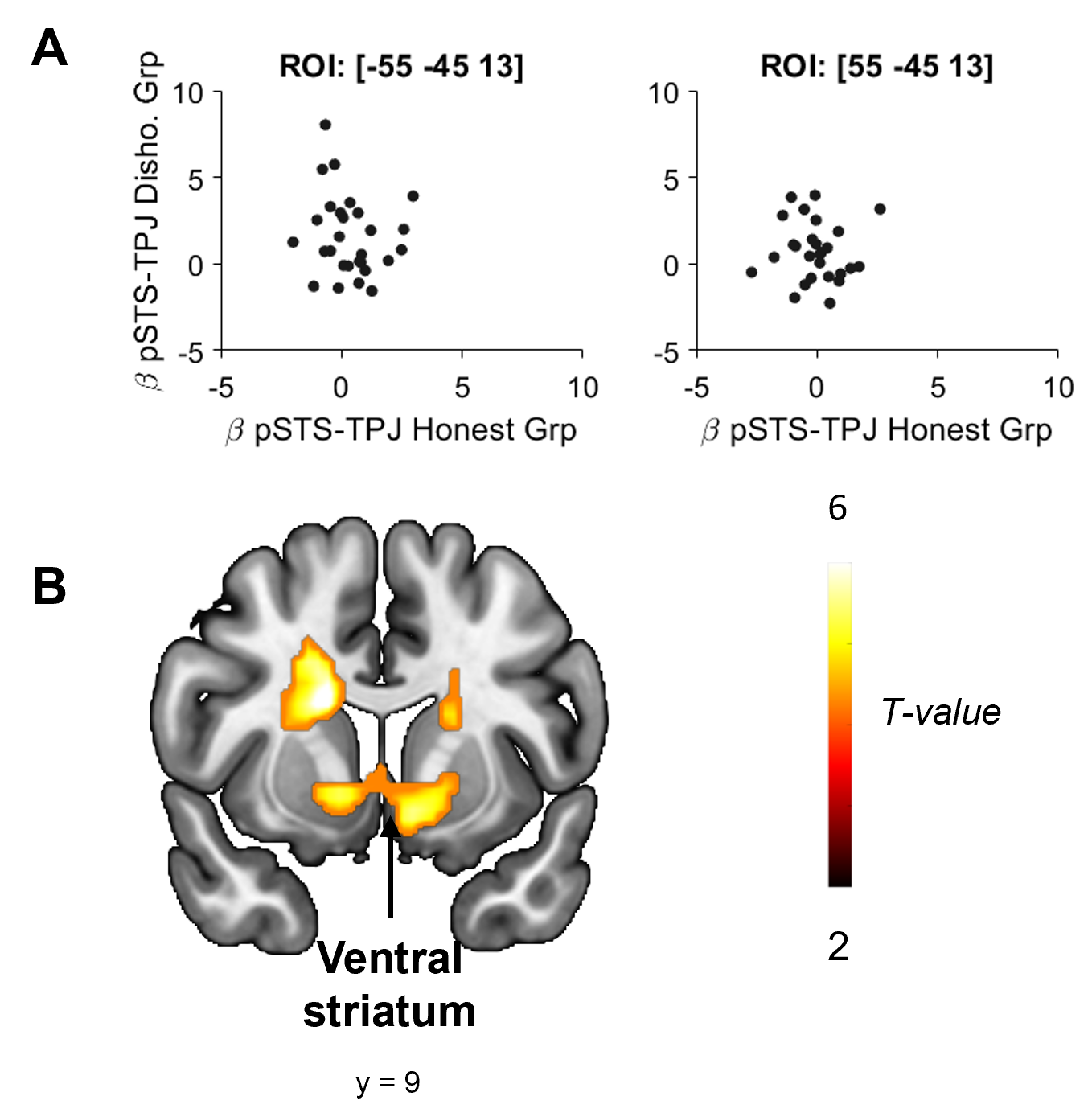

Supplement: S6 Fig — Individual beta estimates extracted from a bilateral pSTS-TPJ ROI [26] in the Dishonest Group condition (y-axis) and in the Honest Group condition (x-axis). The ROI coordinates are [−55 −45 13] (left side) and [55 −45 13] (right side). B. Regions encoding the prediction error from the social learning model at the time of the feedback in the Predict trials (p < 0.001 uncorrected and p < 0.05 whole-brain cluster-corrected family-wise error (FWE)). (TIF) [file pbio.3003889.s007.tif]
