# Supplementary material for: Social learning dynamically shapes moral decision-making by biasing subjective valuation
Source: PLoS Biol. 2026 Jul 10;24(7):e3003889. doi: 10.1371/journal.pbio.3003889 (PMC13379141; doi:10.1371/journal.pbio.3003889)
Supplement: S1 Table — (DOCX) [file pbio.3003889.s008.docx]

**Table S1**: Mean cheating frequency per level of relative pay for cheating for the two groups and model parameters used to simulate the groups’ behavior (Variable Moral cost utility function).

| **Relative pay** | €10 | €8 | €6 | €4 | €2 | Mean |
| --- | --- | --- | --- | --- | --- | --- |
| Honest group | 0.5 | 0.3 | 0 | 0 | 0.3 | 0.22 |
| Dishonest group | 1 | 1 | 1 | 0.7 | 0.7 | 0.88 |
|  |  |  |  |  |  |  |
| **Model Parameters** | *α* | *δ* | *β* | | | |
| Honest group | 0.94 | -0.94 | 2.27 | | | |
| Dishonest group | 1.22 | -0.51 | 1.58 | | | |
