# Supplementary material for: Social learning dynamically shapes moral decision-making by biasing subjective valuation
Source: PLoS Biol. 2026 Jul 10;24(7):e3003889. doi: 10.1371/journal.pbio.3003889 (PMC13379141; doi:10.1371/journal.pbio.3003889)
Supplement: S2 Table — Notes: Relative payoff for cheating: πCheat − πHonest, Diff. dice value: Dice valueCheat − Dice valueHonest. Standard errors clustered at the participant level are in parentheses. *** p < 0.001, ** p < 0.01, * p < 0.05. The data underlying the table can be found in the Tables folder of the OSF repository. (DOCX) [file pbio.3003889.s009.docx]

**Table S2**: Logistic random-effect regressions.

|  | (1) | (2) | (3) |
| --- | --- | --- | --- |
|  | Cheating | Prediction accuracy | Prediction cheating |
|  | (1: Cheat, 0: No cheat) | (1: Correct pred., 0: Incorrect pred.) | (1: Lie pred, 0: No cheating pred) |
| Disho. Grp. vs Baseline | 0.148 *** | - | - |
|  | (0.037) | - | - |
| Honest Grp. vs Baseline | 0.047 | - | - |
|  | (0.025) | - | - |
| Disho. Grp vs Hon. Grp. | 0.101 ** | 0.179 *** | 0.540 *** |
|  | (0.027) | (0.022) | (0.033) |
| Honest Grp. first | -0.067 | -0.045 ** | 0.034 |
|  | (0.100) | (0.018) | (0.035) |
| Trial number | *<* 0*.*001 | 0.002 *** | *<* 0*.*001 |
|  | (*<* 0*.*001) | (*<* 0*.*001) | (*<* 0*.*001) |
| Relative payoff cheating | 0.049 *** | 0.010 *** | 0.049 *** |
|  | (0.006) | (0.002) | (0.004) |
| Diff. dice value | -0.013 ** | *<* −0*.*001 | -0.004 |
|  | (0.004) | (0.004) | (0.004) |
| Demographics | Yes | Yes | Yes |
| Number of observations | 4650 | 3100 | 3100 |
| Number of clusters | 31 | 31 | 31 |
| *P > χ*^2^ | *<* 0*.*001 | *<* 0*.*001 | *<* 0*.*001 |

*Notes*: Relative payoff for cheating: *π_Cheat_* −*π_Honest_*, Diff. dice value: *Dice value_Cheat_* −*Dice value_Honest_*. Standard errors clustered at the participant level are in parentheses. *** p<0.001, ** p<0.01, * p<0.05. The data underlying the table can be found in the Tables folder on the OSF repository.
