# Supplementary material for: Social learning dynamically shapes moral decision-making by biasing subjective valuation
Source: PLoS Biol. 2026 Jul 10;24(7):e3003889. doi: 10.1371/journal.pbio.3003889 (PMC13379141; doi:10.1371/journal.pbio.3003889)
Supplement: S3 Table — Notes: Relative payoff for cheating: πCheat − πHonest, Diff. dice value: Dice valueCheat − Dice valueHonest. Standard errors clustered at the participant level are in parentheses. *** p < 0.001, ** p < 0.01, * p < 0.05. The data underlying the table can be found in the Tables folder of the OSF repository. (DOCX) [file pbio.3003889.s010.docx]

**Table S3**: Additional logistic random-effect regressions.

|  | (1) | (2) |
| --- | --- | --- |
|  | Cheating | |
|  | (1: Cheat, 0: No cheat) | |
| Disho. Grp. vs Baseline | 0.148 *** | 0.148 *** |
|  | (0.036) | (0.036) |
| Honest Grp. vs Baseline | 0.047 | 0.047 |
|  | (0.025) | (0.025) |
| Disho. Grp vs Hon. Grp. | 0.101 ** | 0.101 ** |
|  | (0.028) | (0.028) |
| Honest Grp. first | -0.057 | -0.086 |
|  | (0.097) | (0.088) |
| Trial number | *<* 0*.*001 | *<* 0*.*001 |
|  | (*<* 0*.*001) | (*<* 0*.*001) |
| Relative payoff cheating | 0.049 *** | 0.049 *** |
|  | (0.006) | (0.006) |
| Diff. dice value | - 0.013 ** | - 0.013 ** |
|  | (0.004) | (0.004) |
| Avg. prediction accuracy Disho group | 1.535 | - |
|  | (0.910) | - |
| Avg. prediction accuracy Honest group | -0.835 * | - |
|  | (0.335) | - |
| Avg. prediction accuracy diff | - | 1.036 ** |
|  | - | (0.379) |
| Demographics | Yes | Yes |
| Number of observations | 4650 | 4650 |
| Number of clusters | 31 | 31 |
| *P > χ*^2^ | *<* 0*.*001 | *<* 0*.*001 |

*Notes*: Relative payoff for cheating: *π_Cheat_* −*π_Honest_*, Diff. dice value: *Dice value_Cheat_* −*Dice value_Honest_*. Standard errors clustered at the participant level are in parentheses. *** p<0.001, ** p<0.01, * p<0.05. The data underlying the table can be found in the Tables folder on the OSF repository.
