# Supplementary material for: Social learning dynamically shapes moral decision-making by biasing subjective valuation
Source: PLoS Biol. 2026 Jul 10;24(7):e3003889. doi: 10.1371/journal.pbio.3003889 (PMC13379141; doi:10.1371/journal.pbio.3003889)
Supplement: S4 Table — Effect of a change in beliefs about the morality of cheating in the task. Notes: Standard errors clustered at the participant level are in parentheses. *** p < 0.001, ** p < 0.01, * p < 0.05. The data underlying the table can be found in the Tables folder of the OSF repository. (DOCX) [file pbio.3003889.s011.docx]

**Table S4**: Logistic random-effect regressions. Effect of a change in beliefs about the morality of cheating in the task.

|  | (1) | (2) | (3) | (4) | (5) |
| --- | --- | --- | --- | --- | --- |
|  | Lying | Lying | Lying | Prediction accuracy | Prediction cheating |
|  | Moral beliefs changed | Moral beliefs  did not change | Interaction |  |  |
| Disho. Grp. vs Baseline | 0.116 * | 0.174 ** | - | - | - |
|  | (0.043) | (0.057) | - | - | - |
| Honest Grp. vs Baseline | 0.060 | 0.039 | - | - | - |
|  | (0.045) | (0.028) | - | - | - |
| Disho. Grp vs Hon. Grp. | 0.056 *** | 0.135 * | - | - | - |
|  | (0.012) | (0.047) | - | - | - |
| Effect of morality beliefs in Baseline | - | - | 0.063 | - | - |
|  | - | - | (0.125) | - | - |
| Effect of morality beliefs in Disho. Grp. | - | - | 0.009 | - | - |
|  | *-* | - | (0.107) | - | - |
| Effect of morality beliefs in Hon. Grp. | *-* | - | 0.085 | - | - |
|  | - | - | (0.108) | - | - |
| Change in beliefs about morality of cheating | *-* | - | - | -0.005 | -0.029 |
|  | - | - | - | (0.018) | (0.041) |
| Other variables | Yes | Yes | Yes | Yes | Yes |
| Demographics | Yes | Yes | Yes | Yes | Yes |
| Number of observations | 1950 | 2700 | 4650 | 3100 | 3100 |
| Number of clusters | 13 | 18 | 31 | 31 | 31 |
| *P > χ*^2^ | *<* 0*.*001 | *<* 0*.*001 | *<* 0*.*001 | *<* 0*.*001 | *<* 0*.*001 |

*Notes*: Standard errors clustered at the participant level are in parentheses. *** p<0.001, ** p<0.01, * p<0.05. The data underlying the table can be found in the Tables folder on the OSF repository.
