# Supplementary material for: Social learning dynamically shapes moral decision-making by biasing subjective valuation
Source: PLoS Biol. 2026 Jul 10;24(7):e3003889. doi: 10.1371/journal.pbio.3003889 (PMC13379141; doi:10.1371/journal.pbio.3003889)
Supplement: S5 Table — Notes: cluster reported at p < 0.05 FWE whole brain cluster corrected (initial cluster-forming threshold of p < 0.001 uncorrected). (DOCX) [file pbio.3003889.s012.docx]

**Table S5**: Brain regions encoding the Dynamic Valuation Bias in the Solo trials of the Dishonest Group condition modulated by the participants’ conformity parameter *γ*.

| MNI peak cluster coordinates: | x | y | z | k-cluster | T value |
| --- | --- | --- | --- | --- | --- |
| **Negatively** |  |  |  |  |  |
| left lPFC | -48 | 42 | 0 | 179 | 4.98 |
| right dlPFC | 30 | 39 | 42 | 291 | 5.61 |
| left IPS | -33 | -63 | 45 | 138 | 5.31 |
| right IPS | 36 | -66 | 39 | 157 | 4.46 |
| **Positively**  No Brain region |  |  |  |  |  |

*Notes:* cluster reported at *p <* 0*.*05 FWE whole brain cluster corrected (initial cluster-forming threshold of *p <* 0*.*001 uncorrected).
