# Supplementary material for: Social learning dynamically shapes moral decision-making by biasing subjective valuation
Source: PLoS Biol. 2026 Jul 10;24(7):e3003889. doi: 10.1371/journal.pbio.3003889 (PMC13379141; doi:10.1371/journal.pbio.3003889)
Supplement: S6 Table — Notes: cluster reported at p < 0.05 FWE whole brain cluster corrected (initial cluster-forming threshold of p < 0.001 uncorrected). (DOCX) [file pbio.3003889.s013.docx]

**Table S6**: Brain regions encoding the probability of the participants’ prediction at the time of the prediction in the Predict trials of the Dishonest Group condition.

| MNI peak cluster coordinates: | x | y | z | k-cluster | T value |
| --- | --- | --- | --- | --- | --- |
| **Positively** |  |  |  |  |  |
| right ventral premotor | 57 | 6 | 24 | 6521 | 6.31 |
| left dlPFC | -27 | 30 | 27 | 110 | 5.34 |
| Cerebellum | 18 | -66 | -54 | 182 | 4.65 |
|  | -18 | -63 | -21 | 120 | 4.39 |
|  | -15 | -69 | -54 | 172 | 5.84 |
|  | 15 | -57 | -18 | 207 | 4.25 |
| **Negatively**  No Brain region |  |  |  |  |  |

*Notes:* cluster reported at *p <* 0*.*05 FWE whole brain cluster corrected (initial cluster-forming threshold of *p <* 0*.*001 uncorrected).
