# Supplementary material for: Social learning dynamically shapes moral decision-making by biasing subjective valuation
Source: PLoS Biol. 2026 Jul 10;24(7):e3003889. doi: 10.1371/journal.pbio.3003889 (PMC13379141; doi:10.1371/journal.pbio.3003889)
Supplement: S7 Table — Contrast between the two Group conditions. Notes: cluster reported at p < 0.05 FWE whole brain cluster corrected (initial cluster-forming threshold of p < 0.001 uncorrected). (DOCX) [file pbio.3003889.s014.docx]

**Table S7:** Brain regions encoding the probability of the participants’ prediction at the time of the prediction in the Predict trials. Contrast between the two Group conditions.

| MNI peak cluster coordinates: | x | y | z | k-cluster | T value |
| --- | --- | --- | --- | --- | --- |
| **Dishonest Group > Honest Group** |  |  |  |  |  |
| left Thalamus (left temporal lobe) | -18 | -18 | 3 | 1260 | 5.17 |
| right dlPFC | 60 | 6 | 27 | 300 | 5.14 |
| right Putamen | 24 | -18 | 9 | 129 | 4.60 |
| **Honest Group > Dishonest Group**  No Brain region |  |  |  |  |  |

*Notes:* cluster reported at *p <* 0*.*05 FWE whole brain cluster corrected (initial cluster-forming threshold of *p <* 0*.*001 uncorrected).
