# Supplementary material for: Social learning dynamically shapes moral decision-making by biasing subjective valuation
Source: PLoS Biol. 2026 Jul 10;24(7):e3003889. doi: 10.1371/journal.pbio.3003889 (PMC13379141; doi:10.1371/journal.pbio.3003889)
Supplement: S8 Table — Notes: cluster reported at p < 0.05 FWE whole brain cluster corrected (initial cluster-forming threshold of p < 0.001 uncorrected). (DOCX) [file pbio.3003889.s015.docx]

**Table S8**: Brain regions encoding the prediction error at the time of the feedback in the Predict trials.

| MNI peak cluster coordinates: | x | y | z | k-cluster | T value |
| --- | --- | --- | --- | --- | --- |
| Caudate | 18 | -15 | 24 | 3918 | 5.46 |

*Notes:* cluster reported at *p <* 0*.*05 FWE whole brain cluster corrected (initial cluster-forming threshold of *p <* 0*.*001 uncorrected).
