# Supplementary material for: Social learning dynamically shapes moral decision-making by biasing subjective valuation
Source: PLoS Biol. 2026 Jul 10;24(7):e3003889. doi: 10.1371/journal.pbio.3003889 (PMC13379141; doi:10.1371/journal.pbio.3003889)
Supplement: S9 Table — B, D and H mean that the given parameter is estimated separately for the Baseline (B), the Dishonest Group condition (D) and the Honest Group condition (H). (DOCX) [file pbio.3003889.s016.docx]

**Table S9. Summary of the free parameters of each social influence model.** B, D and H mean that the given parameter is estimated separately for the Baseline (B), the Dishonest Group condition (D) and the Honest Group condition (H).

| **Social influence model** | **Utility function** | **Learning model** |
| --- | --- | --- |
| PS_Fixed_ | $\boldsymbol{\alpha}_{\boldsymbol{(B,D,H)}}\boldsymbol{;}\boldsymbol{\delta}_{\boldsymbol{(B,D,H)}}\boldsymbol{;}\boldsymbol{\beta}_{\boldsymbol{Self}}$ | **-** |
| VB_Fixed_ | $\boldsymbol{\alpha;\delta;}\boldsymbol{\theta}_{\boldsymbol{(D,H)}}\boldsymbol{;}\boldsymbol{\beta}_{\boldsymbol{Self}}$ | **-** |
| PS_Dynamic_ | $\boldsymbol{\alpha;\delta;}\boldsymbol{\omega}_{\boldsymbol{A(D,H)}}\boldsymbol{;}\boldsymbol{\omega}_{\boldsymbol{D(D,H)}}\boldsymbol{;}\boldsymbol{\beta}_{\boldsymbol{Self}}$ | $\boldsymbol{\beta}_{\boldsymbol{Oth(D,H)}}$ |
| VB_Dynamic_ | $\boldsymbol{\alpha;\delta;}\boldsymbol{\gamma}_{\boldsymbol{(D,H)}}\boldsymbol{;}\boldsymbol{\beta}_{\boldsymbol{Self}}\boldsymbol{;}\boldsymbol{\beta}_{\boldsymbol{Predict}}$ | $\boldsymbol{\beta}_{\boldsymbol{Oth(D,H)}}$ |
|  | | |
