# Supplementary material for: Social learning dynamically shapes moral decision-making by biasing subjective valuation
Source: PLoS Biol. 2026 Jul 10;24(7):e3003889. doi: 10.1371/journal.pbio.3003889 (PMC13379141; doi:10.1371/journal.pbio.3003889)
Supplement: Attachment — Submitted filename: Rebuttal letter.pdf [file pbio.3003889.s017.pdf]

Reviewer #1: The authors investigate how social influence of either an honest or a dishonest group influence the cheating behavior of human participants and how the related computations are represented in the brain using model-informed fMRI. In a 3-step procedure they identified best-fitting variants of different computational models and linked them together to explain cue selection in Solo trials, prediction response in Predict trials, and social influence during learning. The primary fMRI finding reveals a rather specific and complex effect that links lPFC activity to a valuation bias (derived from the computational model and modeled at the 1st level), but only when observing the dishonest group (modeled as the person-specific parameter gamma (conformity) at the 2nd level.

I am conflicted about this manuscript. I really like the elegant design of the study and the questions that is tries to answer. I also think that the multi-step procedure to identify the best-fitting model of all the data that are collected (cue selections and predictions) is very clear and makes a lot of sense. I find that some of the variants that are tested in the model comparison may not be the most relevant. More importantly, however, the results from the parameter recovery analysis appeared to be flawed and cast question on the behavioral and neuroimaging findings.

Successful parameter recovery analysis insures that the fitted parameters from simulated data are close the generating parameter that were used to simulate the data. This implies that the scatter plot of fitted vs. true parameters should be grouped around the identity line. Plotting the regression line fitted = a x (true) + b does not insure this unless a ~ 1 and b ~ 0. This is only approximately the case for gammaH and gammaD parameters. But even for those the spread around the regression line (which is close to the identity line) is huge (e.g. a generating parameter of gammaD = 1 could result in a fitted parameter between -1 and 1.8. My conclusion from this recovery analysis is that variance of the true parameters is huge and therefore fitted parameters from experimental data cannot be trusted. Thus, differences between parameters (as reported in Fig S4a) are likely not reliable. And the model-informed fMRI analyses may also be compromised because they are derived from the fitted model with uncertain reliability of its parameters.

My recommendation would be to take a close look at correlations between parameters of the winning model to identify parameter that may be modeling the same aspect of the data and, if so, maybe these parameters can be reduced to a single parameter. In addition, recovery may be improved by reparameterization of the model. For instance, it is well known that the softmax temperature in its original parameterization is difficult to recover, but often can be improved by fitting a log-temperature model. Similar reparameterization may also improve recoverability of other parameters as well.

We thank the reviewer for this thoughtful and detailed comment. We agree that parameter recoverability is a critical issue for the interpretation of both the behavioral and neuroimaging results, and we took these concerns very seriously in revising the manuscript.

In response to the reviewer's suggestions, we implemented several model refinements aimed at improving parameter identifiability. First, we merged the two temperature parameters that govern learning about others' behavior into a single temperature parameter. Second, we

used this same parameter to compute the dynamic valuation bias, to replace the previously distinct $\beta_{predict}$ parameter. Together, these changes substantially reduced model complexity by collapsing three poorly recovered parameters into a single one.

Regarding reparameterization, the reviewer correctly notes that inverse temperature parameters can be difficult to recover in their standard form. In our original implementation, temperature parameters were already exponentiated to ensure positivity, hence effectively corresponding to a log-temperature parameterization. We now clarify this point explicitly in the Methods as follows (page 21): *"All temperature parameters that govern choice stochasticity and social learning as well as parameter α were constrained to be strictly positive by estimating them in exponential form."*

Our changes with respect to merging the three previous temperature parameters that govern learning about others' behavior and participants' valuation bias into one parameter led to an improved model fit for the $VB_{Dynamic}$ model (see new Fig. 3 and Fig. S1 below) and improved model recovery performance (see new Fig. S2.A–B below). Nevertheless, parameter recovery remains imperfect, particularly for the temperature parameter associated with learning about others' behavior. To provide a more nuanced and transparent assessment of this limitation, we now report full scatter plots of true versus recovered parameters and explicitly highlight the 5% of simulations with the poorest fits (see revised Fig. S3). Importantly, these outlier simulations account for most of the apparent dispersion around the regression line, which could otherwise give the misleading impression that parameter variance is uniformly large across simulations.

It is crucial to note that the parameters central to our main conclusions, i.e. the <u>conformity parameters γ</u>, are relatively well-recovered. Most of the spread around the linear regression line is because of outlier simulation (from the 5% of simulations with the poorest fits). Moreover, our behavioral conclusions do not rely on fine-grained differences between poorly recovered parameters. The asymmetry of social influence is supported by converging behavioral evidence (mixed-effects regressions) and neural results that track model-derived signals rather than raw parameter values.

Finally, we now fully acknowledge in the Discussion that imperfect parameter recovery represents a limitation of the current study (see below and pages 14-15). We emphasize that our experiment was not originally optimized for parameter identifiability across multiple complex components (learning and influence), and that future work should build on this framework with designs explicitly tailored to improve recovery. We nevertheless believe that the present study provides an important first step towards a dynamic, neurocomputational account of social influence on moral decision-making.

"A limitation of our study is that the parameter-recovery analyses do not perfectly support the validity of our model-fitting procedure (Fig. S3.A). This likely reflects the fact that our experimental design was not originally optimized for parameter identifiability across multiple complex components, namely social learning and social influence, which are estimated together in the $VB_{Dynamic}$ model. Specifically, our parameter recovery is weak for one parameter (the others' temperature parameter, β_{Oth}). However, the main parameters of interest of our $VB_{Dynamic}$ model, the two conformity parameters γ_H and γ_D, are relatively well-recovered. The limited recoverability of the temperature parameter may reflect, at least in part, the rapid learning observed in participants. Faster learning likely reduced variability in the inferred

relative value of cheating across Predict trials, thereby constraining the information available to estimate parameters related to decision stochasticity. Given that the temperature parameter primarily modulates variability in prediction behavior during social learning, this reduction in variability may have contributed to its poor recovery. Thus, these model-based results should be interpreted with caution and can be seen as a first step toward a better integration of social learning into social influence processes. Future work may build on the present framework using experimental designs tailored to improve parameter identifiability, by increasing environmental volatility or uncertainty to slow learning dynamics."

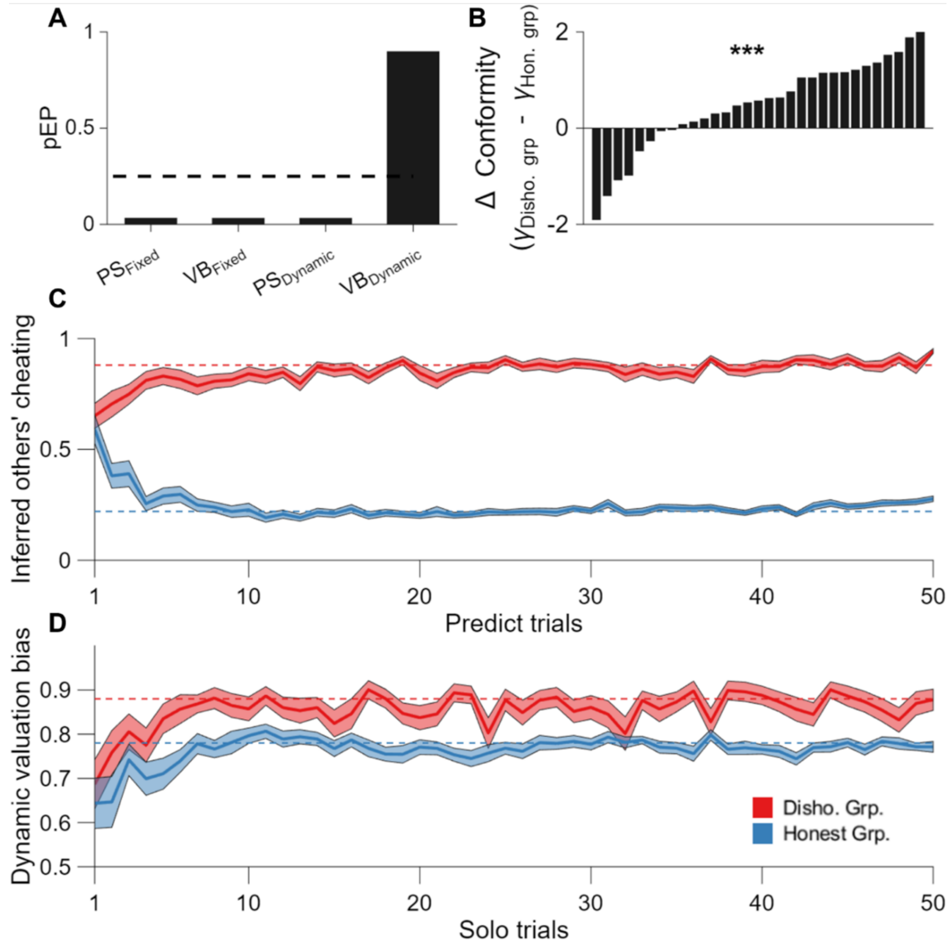

Figure 3. Model selection and estimation. A. Protected Exceedance Probability (pEP) of the different candidate models. The higher the pEP, the more likely a given model explains the group's behavior relative to the others. PS: Preferences shift; VB: Valuation Bias. The dashed line indicates chance level. **B**. Difference between the value of the conformity parameter γ in the Dishonest Group condition and the conformity parameter γ in the Honest Group condition for each participant. The stars represent the result of a two-sided rank-sum test between the two conditions. **C**. Mean inferred probability that others cheated during Predict trials in the Dishonest Group condition (red) and the Honest Group condition (blue). Shaded areas are standard errors. The red (blue) dashed line corresponds to the mean proportion of cheating of the Dishonest (Honest) group members. **D**. Mean dynamic valuation bias over Solo trials in the Dishonest Group condition (red, probability that others would have cheated) and in the Honest Group condition (blue, probability that others would have been honest). Shaded areas are standard errors. The red (blue) dashed line corresponds to the mean proportion of cheating (honest behavior) of the Dishonest (Honest) group members. *** $p < 0.001$.

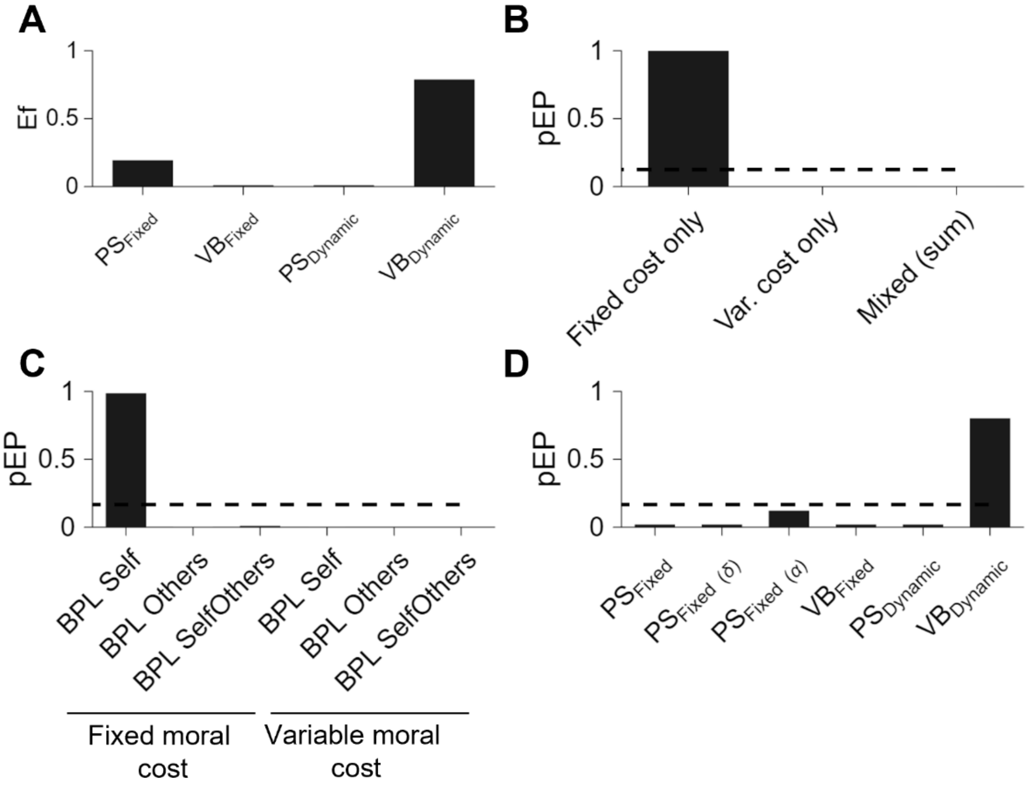

Figure S1: Utility function and social learning model selection. **A**. Estimated model frequencies (Ef) of our 4 candidate models. Black bars show the probability that each model explains the participants' behavior independently of the other models. **B.** Protected Exceedance Probability (pEP) of the different utility functions associated with cheating, estimated over the three blocks of the experiment. The higher the pEP the more likely a given model explains the group's behavior better than the others. The fixed cost only utility function assumes that participants are incurring a fixed moral cost when choosing to cheat, in contrast, the variable cost variant assumes that this moral cost depends on the cheating payoff. The mixed variants are all the possible combinations of these two utility functions across the three blocks of the experiment (baseline and group conditions). **C.** Protected Exceedance Probability (pEP) of the different social learning models. The higher the pEP the more likely a given model explains the group's behavior better than the others. We tested a total of 6 models, all based on the Bayesian Preference Learning model (BPL), for which we varied the priors concerning the others' parameters (Self, Others or SelfOthers) and whether the utility function that the participants used to learn about the others' cheating behavior was the fixed cost or the variable cost utility function. **D.** Protected Exceedance Probability (pEP) of the social influence models, which included two variants of the Preference Shift Fixed model. The variant α is the same as in the original model except that the parameter α does not change across conditions ($PS_{fixed\ (\alpha)}$). Similarly, the variant δ assumes that δ does not change across conditions ($PS_{fixed\ (\delta)}$).

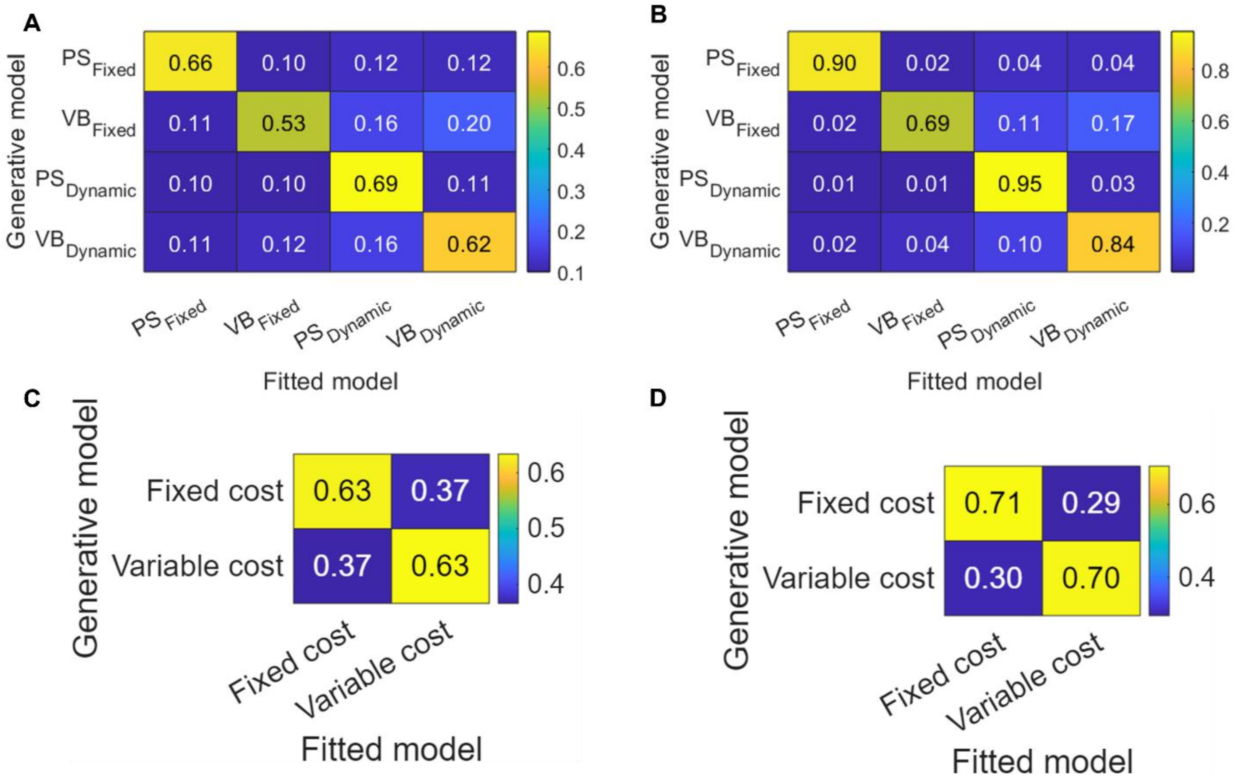

Figure S2: **Model recovery analysis.** We generated data with free parameters randomly selected from a normal distribution centered on the mean of all participants' free parameters, and with its variance being the standard deviation of the participants' individual parameters. Using this procedure, 20 datasets for each of our 28 participants were generated (total of 560 datasets). Then, we ran a model selection on these generated datasets, and repeated this procedure for each model. The rows are the generative models. The columns are the fitted model. **A and C**. The colors represent the mean probability that one model is more frequent than another (in our population of generated datasets) given a Bayesian model selection (Exceedance probability) for the four social influence models (**A**) and the two utility functions considered in our analysis (**C**). **B and D**. The colors represent the mean frequency of each model being the best model to explain the simulations. We assessed which model was the best for each simulation based on which one has the highest Exceedance probability. We followed this procedure for the four social influence models (**B**) and the two utility functions considered in our analysis (**D**).

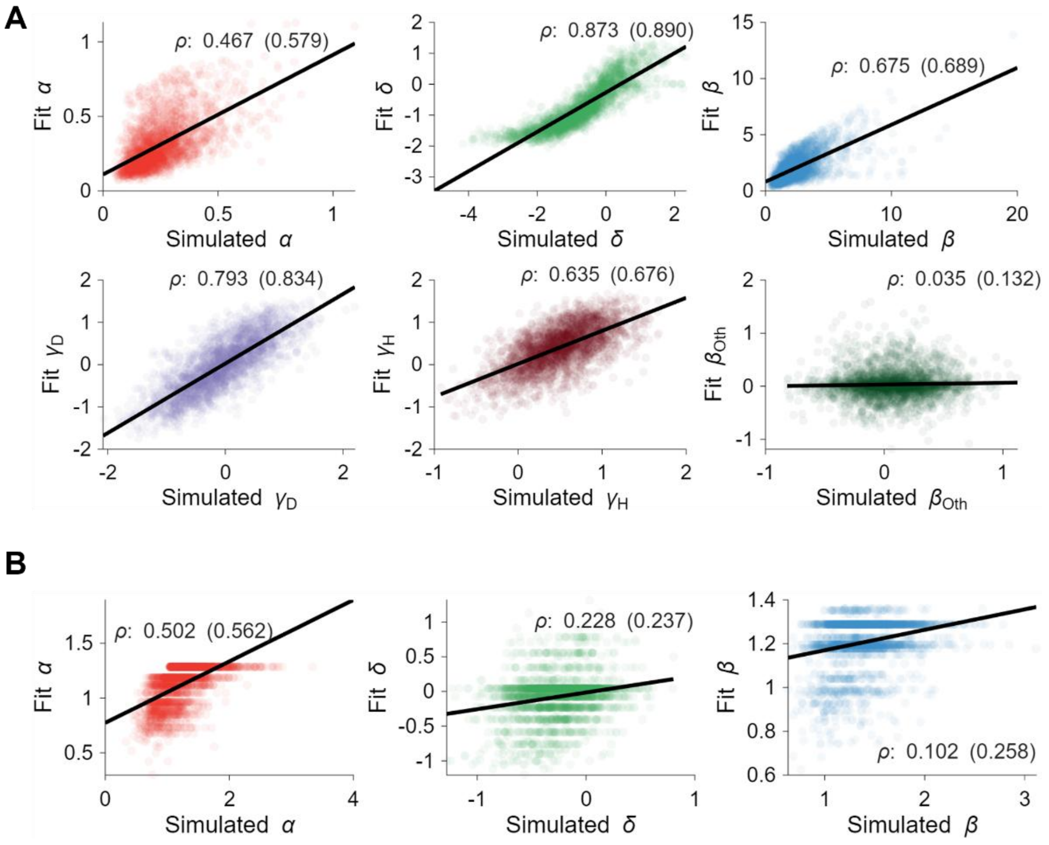

Figure S3: Parameter recovery analysis. A. Simulated and recovered parameters of the $VB_{Dynamic}$ model. The black line represents the linear regression linking the estimated parameters based on simulated choices. ρ is the correlation coefficient. The transparent points represent the 5% of simulations with the poorest fit. The value of ρ in parentheses is the correlation coefficient computed from the remaining simulations. Only the simulated temperature parameter for the other show inconsistency. γ_D, γ_H represent the parameter γ estimated for the Dishonest and Honest Group conditions, respectively. **B**. Simulated and recovered parameters of the Fixed cost utility model. The black line represents the linear regression curve linking the estimated parameters based on simulated choices. ρ is the correlation coefficient. The transparent points represent the 5% of simulations with the poorest fit. The value of ρ in parentheses is the correlation coefficient computed from the remaining simulations. Every correlation represented in this figure is highly significant with $p<0.001$

My second concern is the sparse of the model-informed fMRI analyses. The paper presents a relatively complicated analysis that combined the effects of first-level model-derived variables with second-level person parameters. This comes "out of the blue" without a specific and justified hypothesis of this effect anywhere in the manuscript nor with presenting the findings of the underlying main effects. For me this raises some red flags: is the general basic effect (lPFC coding the dynamic valuation bias) either non-existent and only revealed if one separates the data by the conformity parameter or it is too unspecific and comprises too many brain regions? Anyways, I would like to see a bit more detailed neuroimaging findings that tell the whole story, not just some very specific interaction.

We thank the reviewer for this important comment and agree that the presentation of the model-informed fMRI analyses was initially too sparse. We have therefore substantially revised the neuroimaging Results and Methods to clarify the full pattern of findings, including null results. We have also improved our introduction to better introduce the logic behind our fMRI analysis. The details of these changes are described below and the new figures are included at the end of this answers.

First, we have added prior evidence that emphasizes the importance of inter-individual variability in conformity and moral (honesty-related) preferences to potentially elucidate how the brain encodes dynamic social influence (see below, page 3).

"In addition to these hypotheses, we also considered that inter-individual differences may shape how social information influences cheating behavior. Substantial variability exists in individuals' tendency to conform to others and in their intrinsic honesty, and prior work shows that such inter-individual differences systematically affect how social information is integrated into decision-making (6, 31) and also influence how moral trade-offs are resolved (16, 23). We therefore hypothesized that the dynamic social influence mechanisms captured by our computational models would vary across individuals as a function of their propensity to conform, and that their individual parameters would be linked to their brain activity during decision making."

Second, we now report the main effects of the dynamic valuation bias regressor, independently from the conformity parameter. Importantly, these analyses revealed no significant main effect of the dynamic valuation bias in either Group condition, which we now explicitly describe in the Results (see below, page 10). This null result is now fully documented to clarify that the brain regions reported to encode the dynamic valuation bias are not masking a broader or unspecific activation pattern. We also have updated our GLM, as now GLM 1 only includes one regressor of interest, the dynamic valuation bias signal. It allows us to have a better picture of how this computational signal is encoded in the brain. We also justified our analyses regarding the use of a second level covariate such as the conformity parameter, as we faced a null result. We now found that, not only the left LPFC but also the right dlPFC and the bilateral IPS encode the dynamic valuation bias, modulated by the participants' conformity parameter in the Dishonest Group condition.

"In the first GLM (GLM 1), we included only the first signal (dynamic valuation bias) as a parametric regressor during the choice phase of the Solo trials in both the Dishonest and Honest Group conditions (see Methods). The purpose was to assess whether some brain regions encoded this signal in the two conditions. This GLM revealed no significant correlation between brain activity and the dynamic valuation bias in the Solo trials of either Group condition ($p >$ 0.001 uncorrected). One possible explanation for the absence of a group-level effect is that the neural encoding of the dynamic valuation bias depends on individual differences in conformity to others' behavior. Specifically, participants who behave against the observed norm (e.g., cheating less than the Dishonest Group or more than the Honest Group) may encode this signal differently from participants whose behavior aligns with that of others. Consistent with this

interpretation, when focusing on the Dishonest Group condition, a second-level regression analysis revealed that the relationship between the dynamic valuation bias and brain activity varied as a function of participants' conformity parameter γ. In particular, activity in the left Lateral Prefrontal Cortex (lPFC), the right dorsolateral Prefrontal Cortex (rdlPFC) and bilateral Inferior Parietal Sulcus (IPS) encoded the dynamic valuation bias which was inversely proportional to the conformity parameter, γ, for the Dishonest Group condition (lPFC, x,y,z =-48,42,0; rdlPFC = 30,39,42; IPS = -33,-63,45 and 36,-66,39; Fig. 4A and Table S6; p< 0.05 FWE whole-brain cluster corrected). To illustrate this effect (Fig. 4B), individuals' betas were extracted from 6 mm spheres centered on peak activity in the bilateral lPFC in the Dishonest and Honest Group condition. For negative values of γ, participants show anti-conformity while positive γ values reflect conformity."

Third, we have updated all of our fMRI results as we have made substantial changes to our social influence model. The results are qualitatively the same, except for the relative value of the participants' choice which appears to be encoded in the dACC but failed to pass the whole brain correction (see below, page 10-11).

"In the second GLM (GLM 2), we added the remaining three computational signals: the probability associated with participants' predictions at the time of the prediction in the Predict trials, their prediction error at the time of the feedback in the Predict trials and the relative value of the participants' choice at the choice stage of the Solo trials in every condition (Baseline and Groups; see Methods SI for details). This GLM revealed that the probability associated with participants' predictions in the Dishonest Group condition is encoded in a large cluster that includes the bilateral posterior Superior Temporal Sulcus and Temporo-Parietal Junction (pSTS-TPJ) and the dorsal anterior cingulate cortex (dACC; cluster's peak, x,y,z = 57,6,24 and -27,30,27; see Fig. 5.A and Table S7; surviving a FWE-peak correction, see Fig. S5.A for Betas extracted from an independent ROI). Furthermore, a contrast between the Dishonest and Honest Group condition showed that a part of this cluster activity was significantly greater in the Dishonest Group condition than in the Honest Group condition (cluster's peak, x,y,z = -18,-18,3 and 60,6,27; see Fig. 5.B and Table S8; p<0.05 FWE whole-brain cluster corrected). When looking for regions encoding this computational signal in the Honest Group condition, no voxels survived the cluster-FWE correction (p > 0.05). Additionally, the relative value of the chosen option ($DV_{Choosen} - DV_{Unchoosen}$) in the Solo trials was negatively correlated with the BOLD signal in the dorsal Anterior Cingulate Cortex (dACC, x,y,z = 18,27,48). However, this cluster did not survive the cluster FWE correction (p(FWE-corrected) = 0.203). Finally, the prediction error at the time of the feedback in the Predict trials, correlated with a large cluster encompassing the bilateral ventral striatum (x,y,z = 18,-15,24; Fig. S5.B and Table S9)."

Finally, we have updated the paragraph that describes the construction of the GLM in the Results section as well as in the Methods section, and updated the paragraph in the Discussion that pertains to the brain regions encoding the dynamic valuation bias.

Results section (page 10):

"We constructed three GLMs to identify how the brain encodes the 4 computational signals of the $VB_{Dynamic}$ model. The first three signals concerned the decision and prediction processes (at the time of choice in the Solo trials and the time of prediction in the Predict trials): (1) the dynamic valuation bias in the Dishonest and Honest Group conditions (choices in the Solo trials); (2) the probability associated with participants' predictions, when observing Dishonest and Honest group members (predictions in the Predict trials), (3) the relative value of chosen and unchosen options in Solo trials, regardless of conditions (i.e., all blocks averaged together). The fourth signal corresponded to the prediction error at the feedback stage of the Predict trials. Four parametric regressors of no interest were also added: participants' response times, choice side (left or right), decisions to cheat or not at the choice stage of the Solo trials and their predictions about others' behavior during the prediction stage of the Predict trials. All the parametric regressors were orthogonalized due to a strong correlation between the parametric regressors of interest and those of no interest."

Methods section (page 22):

"We ran general linear model (GLM) analyses to identify brain regions that encode the following computational signals: (1) the dynamic valuation bias in both the Dishonest and Honest Group conditions (choice stage in the Solo trials); (2) the probability associated with participants' predictions, in the Predict trials in both the Dishonest and Honest group conditions (prediction stage in the Predict trials); (3) the relative value of participants' chosen options in Solo trials, regardless of conditions. We also included a fourth signal, the prediction error at the feedback stage of the Predict trials. In every GLM an event was defined as a boxcar function whose duration was equal to the participant's reaction time or to the time of display with the exception of the button press which was defined as a stick function. Events such as the stimulus stage, the choice and prediction stages for both Solo and Predict trials as well as the feedback stage in the Predict trials were always included in all of our GLMs. Head movement parameters were added as parametric regressors of no interest to account for motion related noise. Finally, to control for diverse task parameters, we added as parametric regressors in every GLM for the choice and prediction onsets: the choice or prediction of the participant (0: No cheat, 1: Cheat), the participants' reaction time, and which side was pressed in the choice or prediction stage of the Solo and Predict trials, respectively. All of our parametric regressors were orthogonalized due to strong correlation between the parametric regressors of interest and the model's parameters. Based on these common features we defined 2 GLMs.

In GLM1, we added the following parametric regressor: the inferred others' probability to cheat (be honest) in the choice stage of the Solo trials of the Dishonest (Honest) group condition (dynamic valuation bias, signal 1). In GLM2, we added the following parametric regressors: the probability associated with participants' predictions at the prediction stage of Predict trials in both the Dishonest and Honest Group conditions (signal 2). In the choice stage of the Solo trials, we added the relative value of the participants' choice in every condition (signal 3). Then, in the feedback stage of the Predict trials we added the prediction error from the BPL-Self model in both the Dishonest and Honest Group conditions. Finally, in both GLMs, we added the participants' conformity parameter γ_D or γ_H as a second-level covariate, for the Dishonest and Honest Group conditions respectively."

We computed one sample t-tests with contrasts for main effect for each parametric regressor as well as the main effect per block (Baseline, Dishonest and Honest groups) when possible. Reported brain areas show a significant activity at the threshold of $p < 0.05$, whole brain family-wise error (FWE), corrected for multiple comparisons at the cluster level (threshold at $p < 0.001$ uncorrected).

Discussion (pages 13-14):

"Our findings also shed light on the neurocomputational mechanisms that support social influence. When participants were exposed to a Dishonest Group, we observed differences in dynamic valuation bias–related activity in the right dlPFC and left lPFC as a function of individual conformity levels. Specifically, greater valuation bias–related activity in these regions was associated with lower conformity, which reflects increased resistance to the observed cheating norm. Notably, we did not observe a main effect of the dynamic valuation bias at the group level, which indicates that this neural signal is not uniformly expressed across individuals but rather depends on individual differences in conformity.

This neural pattern mirrors the behavioral results which show that larger valuation biases were associated with a higher likelihood to cheat. The observation that the lPFC dynamically tracks valuation bias extends previous work showing that this region encodes others' perspectives on choice behavior, whether moral or non-moral (6, 16), and plays a role in moral and ethical decision-making through processes of self-regulation, integration of moral values (14, 16, 24, 45–47), and norm compliance (22, 48). Importantly, prior studies examined contexts in which social norms were explicitly known from the outset, rather than learned over time as in the present experiment. Here, we show additionally that the lPFC tracks the evolution of the social norm (i.e., the behavior of others) depending on the participants' degree of conformity. Together, these findings suggest that the lPFC dynamically monitors the social environment and supports the integration of social information into the valuation processes that guide decision-making."

Together, these additions ensure that the neuroimaging results now present the full story, thereby addressing the reviewer's concern about selectivity and interpretability of the reported effects.

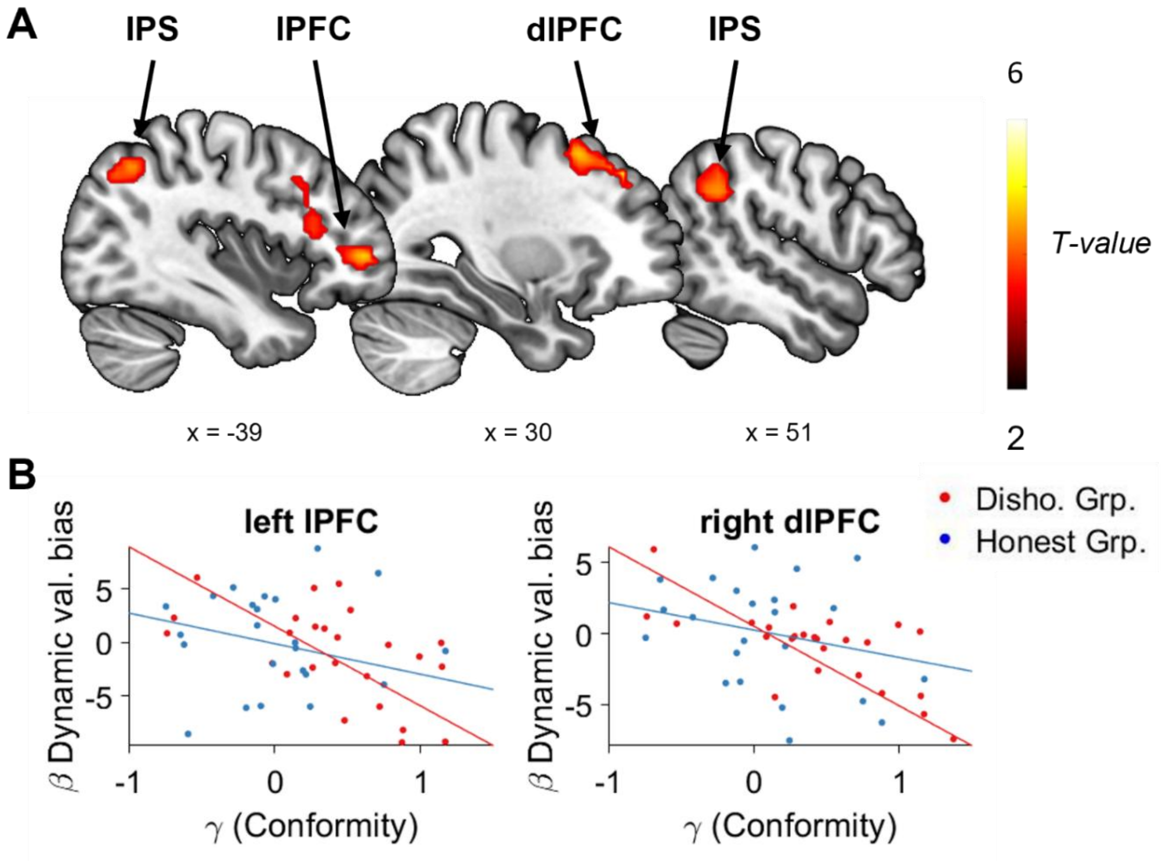

Figure 4. Brain regions encoding the dynamic valuation bias. **A.** Negative relationship between the BOLD signal in the left lateral prefrontal cortex (lPFC) and the dynamic valuation bias signal (i.e., the learned probability that the other would have cheated), as a function of participants' conformity parameter γ, at the decision stage of the Solo trials of the Dishonest Group condition ($p < 0.001$ uncorrected and $p < 0.05$ whole-brain cluster-corrected family-wise error (FWE)). **B.** Individuals' beta extracted from 6mm sphere centered on peak activity in the left lPFC (right dlPFC) are negatively correlated with their conformity parameter γ in the Dishonest Group condition.

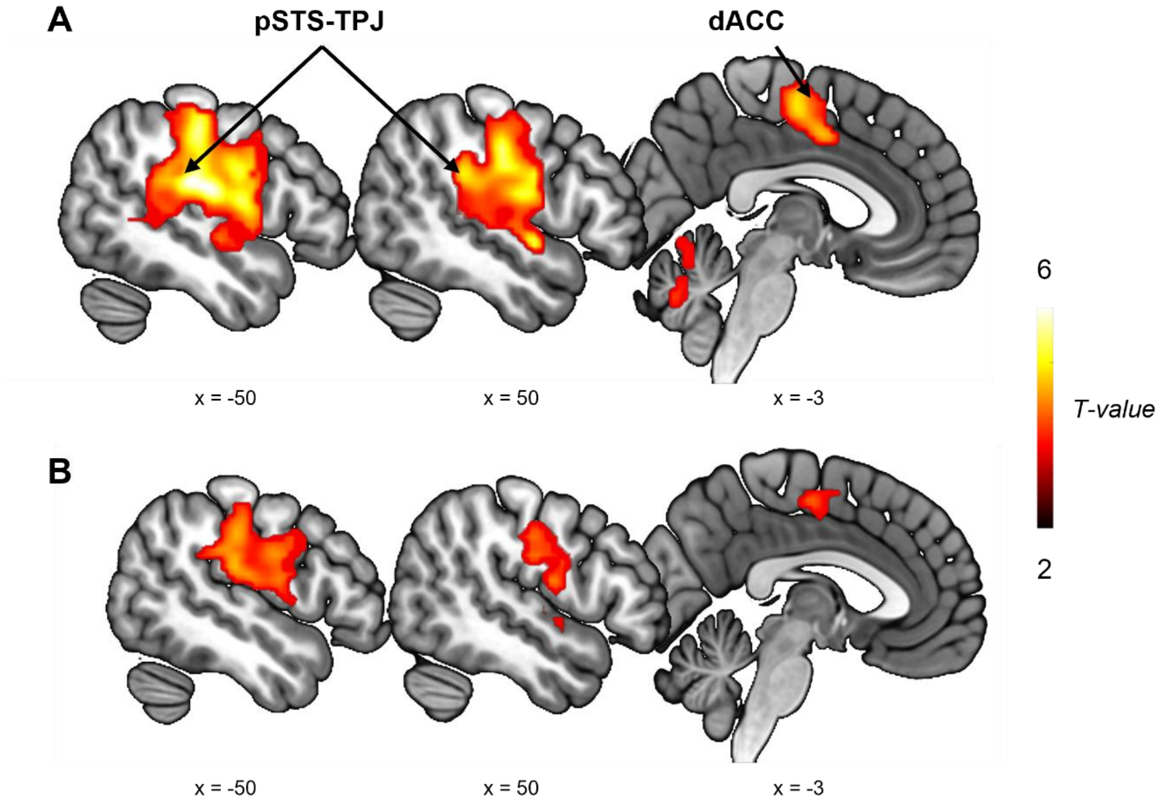

Figure 5. Brain regions involved in learning others' behavior. **A.** The BOLD signal in the bilateral posterior superior temporal sulcus (pSTS), the temporo-parietal junction (TPJ) and the dorsal anterior cingulate cortex (dACC) correlates with the probability associated with participants' predictions in the Dishonest Group condition at the prediction stage of the Predict trials ($p < 0.001$ uncorrected and $p < 0.05$ whole-brain cluster-corrected family-wise error (FWE)). **B**. Contrast between the brain activity in the Dishonest and Honest Group conditions that correlates with the probability associated with participants' predictions at the prediction stage of the Predict trials ($p < 0.001$ uncorrected and $p < 0.05$ whole-brain cluster-corrected family-wise error (FWE)).

Reviewer #2: This study tests the role of social influence in cheating behavior. It extends previous work on social influence by 1. introducing a task and model which capture how others' choices dynamically change as they observe the choices of others and 2. examining how observing others can influence cheating. Further, they show that lPFC tracks the probability of others cheating when the others are dishonest (but not honest) and that the extent to which lPFC tracks it predicts their conformity behavior. One particularly interesting aspect to me was the pitting of a preference shift model against a value bias model. In this study, the authors show that learning about others' cheating behavior shifted individuals' value for cheating rather than changing their internal preferences.

The analyses here are rigorous and thorough. However, I have a few comments, concerns, and suggestions. I ultimately will likely recommend this paper for publication as I think it adds an important and exciting piece to the literature but would like to see these major and minor issues addressed in some capacity beforehand. (The two suggestions are just a couple thoughts that I had that you are totally free to ignore).

Major issues

The two individuals whose behavior was modeled and used to create the decisions for the study participants seemed to utilize a different decision-making model (variable cost of cheating) than the winning model for the task participants. I think you should discuss the potential implications of this for participant's models of others (e.g., the BPL-self model).

We thank you for raising this point. The reviewer is perfectly correct that the behavior of the two pilot participants used to generate the groups' choices was modeled with a utility function that included a variable cost of cheating, whereas the model that best captured task participants' own decisions, and learning, included a fixed cost of cheating, as stated by the reviewer. We agree that this mismatch has potential implications for how participants form models of others' behavior, and we have now added a paragraph in the Discussion to address this issue. The new paragraph reads as follows (page 14):
"The behavior of the individuals used to generate the group decisions was simulated using a utility model that included a variable cost of cheating, whereas the BPL model that best described participants' own decisions and learning relied on a model which included a fixed cost of cheating. In principle, such a mismatch could make learning about others' cheating behavior more difficult, as participants would be inferring patterns generated by a model different from their own. However, our behavioral results suggest that participants were nevertheless able to form accurate beliefs. Their predictions were significantly better than chance and converged toward the true mean cheating level in both Group conditions. Several factors may explain the discrepancy between the generative model of the pilot participants and the model that best fits our main sample. First, the pilot participants completed the task in a different context (outside the MRI scanner), which may have influenced their decision strategies. Second, unobserved demographic or psychological differences between pilot and main-sample participants could also contribute to differences in model parameters.

Nonetheless, our analyses indicate that participants' ability to learn about others' behavior was not impaired in a way that would account for the observed asymmetry in conformity."

Along similar lines, one aspect of the experiment that seems like it might be a bit unbelievable for the participant is for a random sample from a group of 10 suddenly and significantly shifting their dishonesty rates (i.e., the transition from the 1st to the 2nd prediction block). I recognize that the participants indicated they believed they were viewing the choices of real people but I wonder if there is some other measure you could use to 'validate' the 2nd prediction block. Perhaps including first vs second block in a logistic regression on prediction accuracy or cheating? Or showing social influence parameters/behavior in the first vs second block to show they are roughly consistent?

We agree that, without clarification, it may appear surprising that the dishonesty rate of the group would suddenly shift between the two group conditions. However, participants were explicitly informed that the two blocks corresponded to two different groups of individuals, each defined by distinct behavioral tendencies. The instructions stated:

"The people in these two groups completed the same task as you are doing in a previous online study. They only completed Block 1 of the experiment. They therefore received no information about other people's decisions.

We selected them and separated them into two groups according to their behavior. Each individual therefore behaved very similarly to the other members of their group. They performed the task online during the first Covid lockdown. They were mainly recruited via the Institute of Cognitive Sciences Facebook page and were all aged between 18 and 35. For reasons of anonymity, we can't give you any further information about the individuals in these two groups."

Thus, participants were not led to believe that the two groups were composed of individuals with similar behavior. They knew that each in each block they would be making predictions about two different groups of people who exhibited potentially different cheating behavior.

To address your second point, we note that our logistic regressions already included an order variable ("Honest Group First"; Table S2). This variable indicates whether participants encountered the Honest group (= 1) or the Dishonest group first (= 0). We found that this order variable did significantly influence cheating behavior (Model 1) and its presence in our regression model ensures that our main behavioral effect (asymmetry of contagion) is robust to an effect of the order of presentation. However, we note that the order did significantly influence prediction accuracy. Participants were less accurate when they began with the Honest group. This aligns with our broader finding that the Honest group's behavior was more difficult to predict, rather than suggesting that disbelief or reduced attention occurred in the second block.

Importantly, the inclusion of this order variable did not change the main behavioral effect: participants showed increased cheating in the Dishonest Group condition relative to baseline, whereas no significant change was observed in the Honest Group condition.

One of the central claims of the paper is that participants conformed more to the dishonest vs the honest group (and showed neural activity related to the tracking of conformity behavior and others' P(cheat)). However, I believe this is confounded with the greater learning accuracy in the honest vs dishonest group. There are a few issues below related to this. This is an important point and if you are convinced that this is the case and not related to greater learning accuracy, I am certainly open to being convinced. Otherwise, I think it should be addressed in the discussion that an alternative possibility to less conformity to honest others is because their behavior wasn't learned as well (is there any literature demonstrating asymmetric learning between honest/dishonest others or more generally positive/negative behaviors?)

We thank the reviewer for raising this important point. To address it, we revised the analyses previously presented in Table S3 and replaced them with new regression models.

In the revised Table S3, model 1 is a logistic mixed-effects regression predicting participants' cheating behavior. This model replicates the specification used in model 1 of Table S2, with the addition of two covariates that capture participants' average prediction accuracy in the Dishonest and Honest Group conditions (Avg. prediction accuracy Disho/Honest group). The purpose of this analysis was to test whether our main behavioral finding (*i.e.,* the asymmetry of contagion between the Dishonest and Honest Group conditions) was robust to differences in prediction accuracy across conditions. Importantly, the results were unchanged relative to model 1 in Table S2. This indicates that controlling for prediction accuracy within each condition does not alter the effect of Group condition on cheating behavior.

However, this analysis does not directly address whether differences in prediction accuracy between the Dishonest and Honest Group conditions could explain the stronger conformity observed in the Dishonest Group. To test this possibility, we conducted a second regression analysis (model 2, Table S3), identical to model 1 except that it included participants' average difference in prediction accuracy between the two Group conditions as an additional control variable (Avg. prediction accuracy diff). Once again, the results mirrored those in Table S2, with no change in the estimated effect of Group condition on cheating behavior.

Together, these analyses demonstrate that although prediction accuracy differs between the Dishonest and Honest Group conditions, this difference does not explain the absence of behavioral change in the Honest Group condition.

These new analyses are now referenced in the text (page 6). The relevant text and the new Table S3 are included below.

"*To ensure that the observed differences were attributable to the groups' actual behavior, and not differences between their learning performance with respect to the Honest and the Dishonest group's behavior, we first tested whether participants' prediction accuracy exceeded chance. Participants' accuracy was indeed significantly above the chance level of 50% in both conditions (Wilcoxon signed rank tests, z = 4.87 and 4.49 for Dishonest and Honest Group conditions, respectively, p < 0.001 for both groups; Fig. 2.D). Moreover, Fig. 2.E shows that participants' predictions of others' cheating frequencies closely aligned with the groups' mean cheating behavior in both conditions. Consistent with this, participants predicted cheating significantly more often in the Dishonest Group condition than in the Honest Group condition (Margins Dishonest > Honest: 0.540 ± 0.033, z = 16.2, p < 0.001; see model 3 in Table S2). However, participants were also more accurate when predicting the Dishonest group's behavior than that of the Honest group (Margins Dishonest > Honest: 0.179 ± 0.022, z = 8.20, p < 0.001; see model 2 in Table S2). Therefore, we tested whether differences in prediction accuracy could account for the observed difference in cheating behavior between the Dishonest and Honest Group conditions. Using the same regression structure as model 1 in Table S2 but adding participants' average prediction accuracy in each Group condition as a covariate, we found that including accuracy did not alter the effect of group condition on cheating probability (model 1, Table S3). A second regression that controlled for each participant's difference in prediction accuracy between conditions yielded the same result i.e., the difference in cheating behavior across conditions remained unchanged (model 2, Table S3).*"

Table S3: Additional logistic random-effect regressions.

	(1)	(2)
	Cheating (1: Cheat, 0: No cheat)	
Disho. Grp. vs Baseline	0.148 ***	0.148 ***
	(0.036)	(0.036)
Honest Grp. vs Baseline	0.047	0.047
	(0.025)	(0.025)
Disho. Grp vs Hon. Grp.	0.101 **	0.101 **
	(0.028)	(0.028)
Honest Grp. first	-0.057	-0.086
	(0.097)	(0.088)
Trial number	< 0.001	< 0.001
	(< 0.001)	(< 0.001)
Relative payoff cheating	0.049 ***	0.049 ***
	(0.006)	(0.006)
Diff. dice value	- 0.013 **	- 0.013 **
	(0.004)	(0.004)
Avg. prediction accuracy Disho group	1.535	-
	(0.910)	-

Avg. prediction accuracy Honest group	-0.835 *	-
	(0.335)	-
Avg. prediction accuracy diff	-	1.036 **
	-	(0.379)
Demographics	Yes	Yes
Number of observations	4650	4650
Number of clusters	31	31
$P > \chi^2$	< 0.001	< 0.001

Notes: Relative payoff for cheating: $\pi_{Cheat} - \pi_{Honest}$, Diff. dice value: *Dice value*$_{Cheat}$ −*Dice value*$_{Honest}$. Standard errors clustered at the participant level are in parentheses. *** p<0.001, ** p<0.01, * p<0.05.

1. l164: you state that participants learned equally well in both conditions and use tests from chance to support this claim. The proper test to say that learning is similar between groups is directly comparing them. Indeed, you perform this test and show that the dishonest group's behavior is better predicted than the honest group.

We agree that the initial formulation was misleading: "*To ensure that these differences were driven by the groups' behavior, we tested whether participants learned equally well in both conditions.*". We now have reformulated the sentence as (page 6): "*To ensure that the observed differences were attributable to the groups' actual behavior, and not differences between their learning performance with respect to the Honest and the Dishonest group's behavior, we first tested whether participants' prediction accuracy exceeded chance. Participants' accuracy was indeed significantly above the chance level of 50% in both conditions (Wilcoxon signed rank tests, z = 4.87 and 4.49 for Dishonest and Honest Group conditions, respectively, p < 0.001 for both groups; Fig. 2.D).*"

2. One of your arguments as to why more conformity to dishonest isn't related to this learning difference is that the participants discriminated the levels of dishonesty between the groups (l352). I wasn't sure what result this was referring to. I don't think there is anything in the paper or supplementary information that has convinced me that participants had equally accurate knowledge/prediction ability between the dishonest and honest groups (though, I am open to other arguments if I missed data or am interpreting it differently than you).

We thank the reviewer for pointing out that our original phrasing lacked clarity. The results we intended to reference are presented in model 3 of Table S2, which show that participants predicted cheating significantly more often in the Dishonest Group condition than in the Honest Group condition. This pattern indicates that participants did distinguish between the two groups and correctly inferred that one group engaged in more cheating than the other.

Our argument is therefore limited to the point that participants recognized the relative difference in cheating between groups, which is a necessary prerequisite for conformity effects to emerge. We did not mean to imply that participants were equally accurate across the two conditions, indeed, as we now clarify in the revision, accuracy differed between groups.

We now have rephrased the line presenting the mentioned results (page 6): "*Moreover, Fig. 2.E shows that participants' predictions of others' cheating frequencies closely aligned with the groups' mean cheating behavior in both conditions. Consistent with this, participants predicted cheating significantly more often in the Dishonest Group condition than in the Honest Group condition (Margins Dishonest > Honest: 0.540 ± 0.033, z = 16.2, p < 0.001; see model 3 in Table S2).*"

Furthermore, we also rephrased the paragraph you referred to (pages 12-13): "*At the behavioral level, participants changed their cheating behavior when they were exposed to the Dishonest Group, but not the Honest Group. One possible explanation for this asymmetry is that participants learned more accurately about the Dishonest Group than the Honest Group. Consistent with this idea, prediction accuracy was indeed higher in the Dishonest condition. However, in regression analyses, when we controlled for differences in prediction accuracy, the effect of group condition on cheating behavior remained unchanged. Furthermore, the conformity parameter estimated by the $VB_{Dynamic}$ model was significantly larger when participants observed the Dishonest Group than the Honest Group. This indicates a stronger tendency to align behavior with dishonest norms. Importantly, these conformity parameters did not correlate with participants' mean prediction accuracy. These additional analyses show that while learning accuracy differs between conditions, it cannot account for the asymmetric impact of social influence.*"

3. The strongest argument that the learning didn't affect conformity is Figure S4E. However, the variability in the prediction accuracy seems low making it difficult to find any sort of relationship.

Thank you for highlighting this concern. To address the potential issue raised by the limited variability in mean prediction accuracy in Fig. S4E, we conducted an additional analysis in which we correlated, at the participant level, the difference in the estimated conformity parameter between the Dishonest and Honest Group conditions with the difference in participants' average prediction accuracy between the two conditions (Fig. S4F).

This analysis provides a more direct test of whether asymmetries in learning accuracy could explain asymmetries in conformity. We found no significant relationship between these two variables (p=0.975), indicating that participants who showed larger differences in prediction accuracy did not show correspondingly larger differences in conformity.

A mention of this additional analysis is added page 8:

"*Finally, one could argue that participants' conformity may be explained by their own preferences or by the accuracy of their predictions. Yet, these alternative hypotheses are not*

supported by additional analyses which show that γ is neither correlated with the participants' own moral cost δ, nor with participants' average prediction accuracy (Fig. S4. D-F). However, we cannot completely rule out these alternative explanations with the present experimental design."

You can find a reproduction of the Figure S4 below:

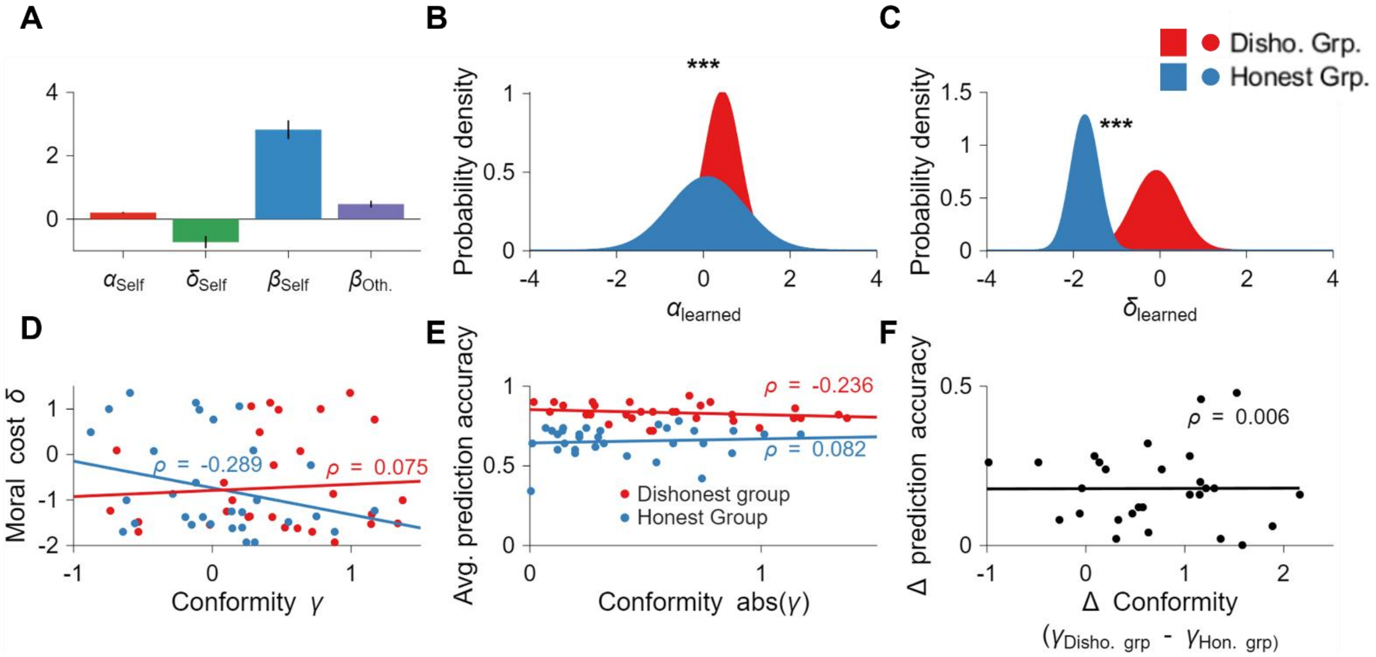

Figure S4: Model selection and estimation. A. Mean values of the participants' $VB_{Dynamic}$ model parameters (left side) and the learned temperature for the Dishonest Group and Honest Group condition (right side). Bars correspond to standard errors. **B-C.** Probability density function of the learned parameters α (**B**) and δ (**C**) for both the Dishonest and Honest Group conditions. Stars correspond to the p-values of a two-sided rank-sum test comparing the mode of distribution between the two conditions. **D**. The participants' moral cost parameter δ is not correlated with the participants' conformity parameter γ in either the Dishonest or Honest Group conditions. The values ρ are the correlation parameters obtained from a Pearson correlation (p=0.689 and p=0.114 for the Dishonest Group and Honest Group conditions, respectively). **E.** The participants' average prediction accuracy is not correlated with the participants' absolute conformity parameter γ in either the Dishonest or Honest Group conditions. The values ρ are the correlation parameters obtained from a Pearson correlation (p=0.201 and p=0.659 for the Dishonest and Honest Group conditions, respectively). *** $p < 0.001$. **F**. The participants' average prediction accuracy difference between the two Group conditions is not correlated with the difference in conformity between the two Group conditions, which indicates that asymmetries in learning accuracy cannot explain asymmetries in conformity. The value ρ = 0.006 is the correlation parameter obtained from a Pearson correlation between the two and it is not significant (p=0.975).

Is there a theoretical maximum for prediction accuracy given that the model of the others' was probabilistic? I.e., someone who has a perfect understanding of the others' model will still predict wrong sometimes because the simulated participant won't be perfectly consistent with their own model. How does this compare between the honest and dishonest group? (If different, this could also be related to the learning asymmetry)

Thank you for raising this important point. In our task, the theoretical maximum prediction accuracy is indeed constrained by the stochasticity of the simulated groups' behavior. This stochasticity is determined by the inverse temperature parameter (β) of the model which generates the groups' choices. Higher β values produce more deterministic behavior and therefore a higher attainable prediction accuracy for participants. Lower β values produce more stochastic behavior and lower the ceiling on prediction accuracy.

As reported in Table S1, the inverse temperature parameter used to simulate the Honest Group behavior was larger than that used for the Dishonest Group. This implies that, if anything, the Honest Group's behavior should have been easier to predict and associated with a higher theoretical maximum prediction accuracy. This pattern therefore predicts the opposite of what one would expect if the learning asymmetry were driven by differences in the predictability of the groups' behavior.

In other words, because the Dishonest Group exhibited lower inverse temperature values (i.e., more stochastic behavior), participants should theoretically have had more difficulty predicting the Dishonest Group compared to the Honest Group. Yet participants were actually more accurate in the Dishonest Group condition.

Minor issues

In the instructions, you were clear to participants that on each trial a new person would be drawn from the group, but this was not clear in the main text of the paper. Please clarify that (probably in the experimental design section of the results).

Thank you for pointing this out. We have now clarified this point. Specifically, we added the following sentences:

Page 4: *"Participants were informed that, on each trial, an individual was randomly selected from a pool of 10 anonymous people who had previously completed the task."*

And page 16: *"This other individual was described as being randomly selected, at the start of each new Predict trial, from a group of 10 participants who had completed the Solo trials in a previous experimental session."*

Figure S4D and E report one set of ρ values in the figure and a second set in the legend. What I think happened is that the values in the legend should be p values and not ρ values.

Thanks for noticing this point, we changed the symbol from ρ to p.

Under the interpretation that conformity < 0 represents recognizing the choices others' make and going against them (i.e., anti-conformity) and that figure S4 is testing if greater understanding of others' behavior is more reflected in conformity behavior, I think figure S4E should use the absolute value of the conformity parameter to compare against

prediction accuracy. If the results change, this may require a different interpretation reflected in the main text.

We have made the change and the results are the same (no significant correlations between the absolute value of conformity and the average prediction accuracy in both Group conditions, see Figure S4.E reproduced above).

Throughout the paper you refer to P(cheat) of others' as "probability of the participant's prediction." This language is a bit awkward and unclear. Consider describing this in a different way for clarity.

We changed the "probability of the participants' prediction" to the "probability associated with participants' predictions" as it refers to the model's probability associated with the prediction made by the participants (That the other was either honest, or cheated).

I'm wondering why you didn't hypothesize that dmPFC/dACC would encode social information/social influence (e.g., Klucharev 2009; 2011)

Indeed, this literature relates to our research question. However, our experiment involves a different behavior (i.e., cheating behavior related to activity in the dlPFC) and previous papers we referred to (Suzuki et al., 2016; Chung et al., 2015) did not report an involvement of the dmPFC in the influence/contagion process. This is why, it sounds more careful, from our point of view, to hypothesize the involvement of dlPFC rather than dmPFC/dACC.

Did you mean to use reference 19 instead of 18 (l54)?

Thank you for noticing this mistake. We have made the change.

I don't particularly have an issue with the temperature parameters not recovering well, but I didn't quite understand the statement "the failure to accurately recover the temperature parameters might be the result of the rapid learning of our participants" (l433) was a bit confusing. Please clarify this.

The idea underlying this statement is that participants appeared to converge rapidly toward the correct mean level of cheating in both Group conditions. This suggests that learning about others' behavior was relatively fast. As a consequence, participants may have quickly become confident in their estimates of others' relative value of cheating, leaving limited scope for the inverse temperature parameter to influence choices. This rapid convergence could therefore reduce the identifiability of the temperature parameter and may help explain the poor recovery of the inverse temperature for others that we observed in our experiment.

We now have rephrased the paragraph referring to this point (pages 14-15):

"*The limited recoverability of the temperature parameter may reflect, at least in part, the rapid learning observed in participants. Faster learning likely reduced variability in the inferred relative value of cheating across Predict trials, thereby constraining the information available to estimate parameters related to decision stochasticity. Given that the temperature parameter primarily modulates variability in prediction behavior during social learning, this reduction in variability may have contributed to its poor recovery.*"

Did you employ smoothing in your fMRI analysis? Please specify either way.

Yes, we did. We have updated the fMRI preprocessing and data analysis section in our Methods (page 22)

"Finally, in order to perform group and individual comparisons, they were co-registered with structural maps and spatially normalized into the standard Montreal Neurological Institute (MNI) atlas space using the DARTEL method with a spatial smoothing using an 8-mm full width at half maximum Gaussian kernel."

Suggestions

I wonder if it would enhance readability to separate the beginning paragraph(s) in behavioral effects of social influence and computational models of social influence into separate sections addressing individual behavior first (e.g., the selection of the fixed vs variable moral cost of cheating in the computational model section before addressing the social influence part).

We now have added subsections (*Utility model selection*, *Social learning model selection* and *Social influence model selection*) in the "Computational models of social influence" section. We hope that this helps improve readability.

It seems to me that Figs 4D and S7 are of equal 'importance' to the paper. To me they both show other 'typical' value-related signaling seen in these types of tasks. Thus, I would suggest including them in the same place.

We now have merged all of our fMRI results in two figures in the main text. However, to keep the number of figures limited we chose not to include the prediction error results in the main text.
