# Supplementary material for: Social learning dynamically shapes moral decision-making by biasing subjective valuation
Source: PLoS Biol. 2026 Jul 10;24(7):e3003889. doi: 10.1371/journal.pbio.3003889 (PMC13379141; doi:10.1371/journal.pbio.3003889)
Supplement: Attachment — Submitted filename: Rebuttal.docx [file pbio.3003889.s019.docx]

REVIEWS:


**Reviewer #2: The authors have adequately addressed my concerns. I just have one final comment regarding their second response which is that I strongly suggest the authors add to the introduction and/or methods to indicate that they instructed participants that the others they were playing with were from different groups sorted by their behavior.**

We thank the reviewer for this suggestion. We have now clarified in the Methods section that participants were informed that the two groups (dishonest and honest groups) differed in their behavior. Specifically, we added the following sentence (page 19):

“*Furthermore, they were informed that the two groups of participants (i.e. honest vs dishonest) did not exhibit the same behavior.”*

**Reviewer #3: I really like the design and the main behavioural results of this paper, yet I feel like there is still some work to be done on improving the model.**

**The authors say that the neural results rely on model-derived signals and not on the parameters, but the signals are a direct consequence of which parameter values are used to simulate the model. While the recovery for gamma-dishonest is good, and of delta very good, other 4 parameters are not recovered sufficiently well. This is judged by the correlation coefficients; I am not sure what the authors meant with the poorest fit criteria for the scatterplot - likelihood of the simulated data under the fitted parameters? If the 95% of other dots have less spread around the identity line, why not showing them as well? Was the significance of the correlations assessed parametrically? If so, even small correlations can become significant by just running more simulations.**

We agree with the reviewer that parameter recovery is an important aspect of model validation and that imperfect recovery can affect the interpretation of model-based results. We also agree that the model-derived signals used in the fMRI analyses depend on the estimated model parameters. We have therefore revised the manuscript to clarify the recovery quality of all parameters more explicitly and to emphasize this limitation.

Page 17: “*Specifically, our parameter recovery was weak for one parameter, the others’ temperature parameter (β_Oth_) and limited for two parameters, α and* $\beta$*. However, the main parameters of interest in the VB_Dynamic_ model, the conformity parameters γ_H_ and γ_D_, were relatively well-recovered.”*

Regarding the parameter recovery plots, we chose to display all simulations for transparency. Because we performed a large number of simulations (n=2800), the scatter plot may appear visually dispersed. However, part of this dispersion is driven by a small proportion of extreme simulations. To clarify this point, we revised the figure legend as follows:

Legend Figure S3: “*The transparent points represent the 5% of simulations that are farthest from the regression line.”*

Finally, we have removed references to the statistical significance of the recovery correlations, as we agree with the reviewer that significance testing is not informative in this context, as it was indeed assessed parametrically, and is strongly influenced by the number of simulations.

**Figure S3: Parameter recovery analysis.** **A**. Simulated and recovered parameters of the VB_Dynamic_ model. The black line represents the linear regression linking the estimated parameters based on simulated choices. ρ is the correlation coefficient. The transparent points represent the 5% of simulations that are farthest from the regression line. The value of ρ in parentheses is the correlation coefficient computed from the remaining simulations. Only the simulated temperature parameter for the other show inconsistency.$\gamma_{D},\gamma_{H}$ represent the parameter $\gamma$ estimated for the Dishonest and Honest Group conditions, respectively. **B.** Simulated and recovered parameters of the Fixed cost utility model. The black line represents the linear regression curve linking the estimated parameters based on simulated choices. ρ is the correlation coefficient. The transparent points represent the 5% of simulations with the poorest fit. The value of ρ in parentheses is the correlation coefficient computed from the remaining simulations.

**What could be an analog of gamma in the raw behavioural data? If you were to fix all other parameters and simulate behaviour with gammas spanning the range from -1 to 1.5, what difference in the simulated data would you expect? Do you see the same variability in the raw data? This would make the results in Figure 4B more credible.**

We thank the reviewer for this helpful suggestion. To provide a more intuitive behavioral interpretation of the conformity parameter γ, we first simulated behavior while varying γ from −1 to 1.5 and fixing all other parameters to each participant’s estimated values. This analysis showed that γ has a clear behavioral interpretation within the model: increasing γ shifts simulated choices toward the behavior of the observed group. Specifically, higher γ values increased simulated cheating in the Dishonest Group condition and reduced simulated cheating in the Honest Group condition, corresponding to greater conformity to the group-specific norm (Figure S5.A, below). We now include a reference to this figure page 9 as follows:

*“This indicated no reliable conformity effect in the Honest Group condition there is a strong conformity effect in the Dishonest Group condition. Indeed, higher γ values indicated a greater tendency to align with others’ behavior (more cheating in the Dishonest Group condition and less cheating in the Honest Group condition, see Fig. S5.A for a simulation).”*

We then tested whether an analog of γ could be observed in the empirical data. We computed the difference between participants’ mean cheating in each Group condition and their mean cheating at baseline, and correlated this difference with the estimated γ parameters. The correlations were in the expected direction, positive in the Dishonest Group condition and negative in the Honest Group condition, but were modest and only significant in the Honest Group condition (see Fig S5.B below). Thus, this behavioral difference provides only a coarse proxy for γ rather than a direct equivalent of the fitted conformity parameter. This may be due to the dynamic aspect of conformity in our experiment, as participants learn over time the true level of dishonesty of the others. We now refer to this result as follows (page 9):

*“**Furthermore, we examined whether an analog of γ could be observed in the raw data by correlating the fitted γ parameters with participants’ change in cheating relative to baseline (mean cheating in each Group condition minus mean cheating in the baseline). These correlations were in the expected direction, positive in the Dishonest Group condition and negative in the Honest Group condition, although the association was weak and only significant in the Honest Group condition (Fig. S5.B). Thus, aggregate changes in cheating provide a coarse behavioral proxy for γ, but should not be interpreted as a direct equivalent of the fitted conformity parameter. This is because such aggregate measures do not capture the dynamic aspect of conformity in our experiment.”*

**Figure S5.** **A.** Mean simulated cheating as a function of γ. We simulated behavior while fixing all parameters to each participant’s estimated values and varying only γ from −1 to 1.5 (by step of 0.1). Higher γ values shifted simulated behavior toward the group norm: increased cheating in the Dishonest Group condition and reduced cheating in the Honest Group condition. **B.** Correlation between fitted γ parameters and the difference between the participants’ mean cheating in each Group condition and their mean cheating in the baseline. The direction of the correlations is consistent with the interpretation of γ as a conformity parameter, although the associations were weak. The values ρ are the correlation parameters obtained from a Pearson correlation (p=0.105 and p=0.010 for the Dishonest Group and Honest Group conditions, respectively). **C.** The participants’ moral cost parameter δ is not correlated with the participants’ conformity parameter γ in either the Dishonest or Honest Group conditions. The values ρ are the correlation parameters obtained from a Pearson correlation (p=0.689 and p=0.114 for the Dishonest Group and Honest Group conditions, respectively). **D.** The participants’ average prediction accuracy is not correlated with the participants’ absolute conformity parameter γ in either the Dishonest or Honest Group conditions. The values of ρ are the correlation parameters obtained from a Pearson correlation (p=0.201 and p=0.659 for the Dishonest and Honest Group conditions, respectively). **E.** The participants’ average prediction accuracy difference between the two Group conditions is not correlated with the difference in conformity between the two Group conditions, which indicates that asymmetries in learning accuracy cannot explain asymmetries in conformity. The value ρ = 0.006 is the correlation parameter obtained from a Pearson correlation between the two and it is not significant (p=0.975).

**My intuition is that the model should be further simplified. For example, what is the effect of beta_other on simulated behaviour? Does removing (or constraining) this parameter improve the recovery of the other ones?**

We thank the reviewer for this suggestion. We agree that removing β_Oth_ is a natural change for simplification, especially given its weak recovery in the parameter-recovery analysis. To assess the role of this parameter, we simulated participants’ cheating behavior using the estimated parameters while varying β_Oth_ across three values. This analysis showed that β_Oth_ had only a marginal impact on simulated cheating behavior (Figure R1A).

We therefore also tested whether removing β_Oth_ improved model performance and identifiability. However, the model-recovery analysis showed that the VB_Dynamic_ model without β_Oth_ was less distinguishable from the VB_Fixed_ model: the probability that the VB_Dynamic_ model explained data generated by the VB_Fixed_ model was relatively high (Figure R1B). Thus, although β_Oth_ has only a limited effect on simulated cheating behavior, removing it reduced the ability of the model-recovery procedure to distinguish between the fixed and dynamic valuation-bias models. We therefore retained β_Oth_ in the model.

**Figure R1. A.** Mean simulated cheating for three values of β_Oth_. We used each participant’s estimated parameters and simulated cheating behavior 300 times for each group condition and each β_Oth_ value, resulting in 8,400 simulations per β_Oth_ value and condition. **B**. Model-recovery analysis for the variant of the VB_Dynamic_ model without β_Oth_. We followed the same procedure as for the original model-recovery analysis reported in the manuscript. The colors represent the mean probability that one model is more frequent than another (in our population of generated datasets), given a Bayesian model selection (Exceedance probability) for the four social influence models

**It could also help to check the dependence of recovery quality on the values of other parameters (see Wilson and Collins '19).**

We thank the reviewer for this suggestion. Following Wilson and Collins (2019), we performed two complementary analyses to assess whether parameter recovery was affected by trade-offs between parameters or by specific regions of the parameter space.

First, we examined whether the recovery procedure introduced correlations between recovered parameters, which can indicate parameter trade-offs when simulated parameters are not themselves correlated. This analysis revealed correlations between the recovered α and δ parameters, and between the recovered β and δ parameters, suggesting partial trade-offs between these parameters (Figure R2). In contrast, the recovered γ parameters were not strongly correlated with the other recovered parameters, nor was β_Oth_.

Second, we directly tested whether recovery quality depended on the values of the generative parameters. For each recovered parameter, we computed the absolute recovery error, defined as the absolute difference between the recovered and simulated values. We then regressed this recovery error on the simulated values of all parameters (Figure R3). This analysis showed that recovery error was not uniformly distributed across parameter space. The recovery error of α, β and βOth depended on their own simulated values: higher simulated values were associated with larger recovery errors. This is in line with the plots shown in Figure S3. The only clear dependence of one parameter’s recovery error on another parameter’s value concerned δ, for which recovery error increased with higher simulated α values. Finally, the γ parameters showed some dependence on other simulated parameters, but these effects were not concentrated in a way that indicated a clear failure of γ recovery in a specific region of parameter space.

Thus, although the model shows some parameter dependencies, the conformity parameters γD and γH remain among the more reliably recovered parameters and do not appear to be strongly driven by a specific failure of recovery in one region of parameter space.

Importantly, these additional analyses did not identify a clear or interpretable modification that would improve the model. The observed dependencies were not concentrated around a single problematic parameter interaction that could be resolved by removing or constraining one parameter. Moreover, as shown in response to the previous comment, removing β_Oth_ reduced model recovery by making the VB_Dynamic_ model less distinguishable from the VB_Fixe_d model. We therefore do not think these analyses provide a sufficient basis for reworking the model structure at this stage. Instead, we believe that the most transparent approach is to retain the original model, and explicitly acknowledge the limits of parameter identifiability as we have done in the discussion.

**Figure R2.** Correlation between recovered parameters following the parameter recovery. The coefficient of the correlation is displayed in each cell.

**Figure R3.** **Dependence of recovery quality on simulated parameter values.** For each parameter, recovery error was defined as the absolute difference between the recovered and simulated values. We then regressed the recovery error of each parameter on the simulated values of all model parameters. Values displayed in each cell correspond to regression coefficients. Larger coefficients indicate that recovery quality for a given parameter depends more strongly on the value of the corresponding simulated parameter. Stars indicate significance levels: *** p < 0.001, ** p < 0.01, * p < 0.05.

**It would also help to report the fitted values of all parameters, plotting the values of individual participants and the correlations between parameters. The values of the parameters should be commented on, e.g., alpha_learned is on average 0 for the honest group, which means that the probability of cheating does not depend on reward outcomes.**

We agree with the reviewer. In the revised manuscript, we now report the fitted values of all parameters of the VB_Dynamic_ model in a new Figure S4. Specifically, we show the distributions of individual participants’ fitted parameters using violin plots (Figure S4.A-D), together with tests assessing whether each parameter differs from zero. Furthermore, we have added a statement that the parameter α, β and β_Oth_ were constrained to be positive in our model. We have thoroughly updated the results section accordingly and we now comment the values of each parameter as follows (pages 9-10):

*“**We then explored the estimated parameters of the winning VB_Dynamic_ model. First, the parameter α (mean: 0.235 ± 0.021, Wilcoxon signed rank test, z = 4.86, p < 0.001, Fig. S4.A) was significantly positive, as well as the inverse temperature parameters β (mean: 2.821 ± 0.293, Wilcoxon signed rank test, z = 4.86, p < 0.001, Fig. S4.A) and β_Oth_ (mean: 1.100 ± 0.062, Wilcoxon signed rank test, z = 4.86, p < 0.001, Fig. S4.A). However, these parameters are constrained in our model to be significantly greater than zero. Conversely, δ is negative and significantly different from zero (mean: -0.727 ± 0.190, Wilcoxon signed rank test, z = -3.41, p = 0.001, Fig. S4.A). Overall, this was consistent with participants’ likelihood of cheating increasing with the relative earnings of cheating, while being limited by a fixed moral cost associated with cheating.*

*Second, we observed that the conformity parameter γ is significantly higher in the Dishonest Group condition than in the Honest Group condition (Two-sided rank sum test, z = 3.45, p<0.001; Fig. 3.B and Fig. S4.B).* *Additionally, the parameter γ is significantly different from zero in the Dishonest Group condition (mean: 0.473 ± 0.106, Wilcoxon signed rank test, z = 3.45, p < 0.001, Fig. S4.B) but not in the Honest Group condition (mean: -0.013 ± 0.094, Wilcoxon signed rank test, z = 0.02, p = 0.984, Fig. S4.B). This indicated no reliable conformity effect in the Honest Group condition while there is a clear conformity effect in the Dishonest Group condition. Indeed, higher γ values indicated a greater tendency to align with others’ behavior (more cheating in the Dishonest Group condition and less cheating in the Honest Group condition, see Fig. S5.A for a simulation). Furthermore, we examined whether an analog of γ could be observed in the raw data by correlating the fitted γ parameters with participants’ change in cheating relative to baseline (mean cheating in each Group condition minus mean cheating in the baseline).* *These correlations were in the expected direction, positive in the Dishonest Group condition and negative in the Honest Group condition, although the association was weak and only significant in the Honest Group condition (Fig. S5.B). Thus, aggregate changes in cheating provide a coarse behavioral proxy for γ, but should not be interpreted as a direct equivalent of the fitted conformity parameter. This is because such aggregate measures do not capture the dynamic aspect of conformity in our experiment. Finally, one could argue that participants’ conformity may be explained by their own preferences or by the accuracy of their predictions. Yet, these alternative hypotheses are not supported by additional analyses which show that γ is neither correlated with the participants’ own moral cost δ, nor with participants’ average prediction accuracy (Fig. S5.C-E). However, we cannot completely rule out these alternative explanations with the present experimental design.*

*Third, concerning the learning of others’ preferences, we found that the predicted probability that others would cheat, estimated by participants, converged towards the average simulated probability to cheat of each group (Fig. 3.C). We also found that the dynamic valuation bias converged towards the mean probability to cheat (to be honest) for the Dishonest (Honest) Group condition (Fig. 4.C).*

*Formally, the learned α parameters are significantly higher for the Dishonest Group than the Honest Group (mean α, Dishonest group: 0.437, Honest Group: 0.090, two-sided rank sum test, z = 4.78, p < 0.001; Fig. S4.C). We also found that both learned α parameters are significantly positive (Wilcoxon signed rank test, z(Dishonest, Honest) = 4.86, p < 0.001). Conversely, the learned moral cost δ parameters are significantly lower for the Honest Group than the Dishonest Group (mean δ, Dishonest group: -0.089, Honest Group: -1.736; two-sided rank sum test, z = 6.46, p < 0.001; Fig. S4.D). Moreover, the learned moral cost δ parameter is significantly different from zero in the Honest Group condition (Wilcoxon signed rank test, z = -4.86, p < 0.001) but not in the Dishonest Group condition (Wilcoxon signed rank test, z = -1.10, p = 0.272). This implies that participants recognized that members of the Dishonest Group were more likely to cheat than members of the Honest Group for a given set of payoffs. However, the variance parameter of the distribution for the learned parameter α is significantly higher for the Honest than the Dishonest Group. Conversely, the variance parameter of the distribution for the learned parameter δ is significantly higher for the Dishonest Group than the Honest one (mean var. α, Dishonest group: 0.396, Honest Group: 0.855; mean var. δ, Dishonest Group: 0.531; Honest Group: 0.312; two-sided rank sum tests, z = -4.41, p < 0.001 and z = 3.18, p = 0.001, respectively). Furthermore, the difference in variance for the parameter δ is significantly lower than the difference for the parameter α (α diff: M = -0.459, δ diff: M = 0.219, two-sided rank sum test, z = -5.86, p < 0.001). This finding parallels the behavioral results which indicate that participants’ prediction accuracy is lower in the Honest Group condition than in the Dishonest Group condition. »*

We have also added a sentence describing the constraint on α, β and β_Oth_ in the Methods part as follows:

Page 20: *“Formally, the relative gains of cheating are weighted by a free parameter α representing the agent’s preference for money (with α>0).”*

Page 21: *“This allows us to compute a relative decision value based on Eq. 1 or Eq. 2 which is then transformed to a probability using a softmax function with a temperature parameter β_Oth_ (with β_Oth_ > 0) that is treated as a free parameter.”*

Page 23: *“**Finally, we used a softmax function with an inverse temperature parameter (β>0) to transform the relative value of cheating into choice probability for each of the four models.”*

Finally, we also added a correlation matrix showing pairwise correlations between all fitted parameters (Figure S4.E). We found a significant negative correlation between parameters β and δ, as well as between γ_D_ and *γ_H_.* This last correlation suggests that participants who conformed more strongly to one group tended to conform less strongly to the other. We now report this new result as follows on page 9:

*“**Finally, we also examined pairwise correlations between fitted parameters (Fig. S4.E). This analysis revealed a negative correlation between β and δ, suggesting that participants with stronger moral costs tended to show less stochasticity in their choices. We also observed a negative correlation between γD and γH, indicating that participants who conformed more strongly to the Dishonest Group tended to conform less to the Honest Group.”*

**Figure S4. Parameter estimation. A**. Violin plot of participants’ estimated VB_Dynamic_ model parameters α_Self_, δ_Self_, β_Self_ and β_Other_. Stars indicate the result of Wilcoxon signed-rank tests assessing whether parameter distributions differed from zero. **B.** Violin plots of the estimated conformity parameters γ_D_ and γ_H_. Bottom stars indicate the result of Wilcoxon signed-rank tests assessing whether each parameter differed from zero. Top stars correspond to the p-values of a two-sided the result of Wilcoxon rank-sum test comparing the parameters value between Group condition (γ_D_ for the Dishonest Group and γ_H_ for the Honest Group). **C-D.** Violin plots of the learned parameters α (C) and δ (D) for both the Dishonest and Honest Group conditions. Bottom stars correspond to the p-values of a Wilcoxon signed rank test assessing whether each parameter differed from zero. Top stars correspond to the p-values of a two-sided the result of Wilcoxon rank-sum test comparing the parameters value between Group condition. **E.** Correlation matrix between fitted parameters. Stars indicate significant correlations. For the whole figure: *** p<0.001, ** p<0.01, * p<0.05, n.s. p ≥ 0.05.
